# Impacts of the 2008 Great Recession on dietary intake: a systematic review and meta-analysis

**DOI:** 10.1186/s12966-021-01125-8

**Published:** 2021-04-29

**Authors:** Rosemary H. Jenkins, Eszter P. Vamos, David Taylor-Robinson, Christopher Millett, Anthony A. Laverty

**Affiliations:** 1 Public Health Policy Evaluation Unit, Department of Primary Care and Public Health, School of Public Health, Imperial College London, Charing Cross Campus; The Reynolds Building, St Dunstan’s Road, London, W6 8RP UK; 2 Department of Public Health, Policy and Systems, Institute of Population Health, University of Liverpool, Waterhouse Building Block B, 2nd Floor, Liverpool, L69 3BX UK

**Keywords:** Nutrition, Economic recession, Dietary intake, Economy, Food

## Abstract

**Background:**

The 2008 Great Recession significantly impacted economies and individuals globally, with potential impacts on food systems and dietary intake. We systematically reviewed evidence on the impact of the Great Recession on individuals’ dietary intake globally and whether disadvantaged individuals were disproportionately affected.

**Methods:**

We searched seven databases and relevant grey literature through June 2020. Longitudinal quantitative studies with the 2008 recession as the exposure and any measure of dietary intake (energy intake, dietary quality, and food/macronutrient consumption) as the outcome were eligible for inclusion. Eligibility was independently assessed by two reviewers. The Newcastle Ottawa Scale was used for quality and risk of bias assessment. We undertook a random effects meta-analysis for changes in energy intake. Harvest plots were used to display and summarise study results for other outcomes. The study was registered with PROSPERO (CRD42019135864).

**Results:**

Forty-one studies including 2.6 million people met our inclusion criteria and were heterogenous in both methods and results. Ten studies reported energy intake, 11 dietary quality, 34 food intake, and 13 macronutrient consumption. The Great Recession was associated with a mean reduction of 103.0 cal per adult equivalent per day (95% Confidence Interval: − 132.1, − 73.9) in high-income countries (5 studies) and an increase of 105.5 cal per adult per day (95% Confidence Interval: 72.8, 138.2) in middle-income countries (2 studies) following random effects meta-analysis. We found reductions in fruit and vegetable intake. We also found reductions in intake of fast food, sugary products, and soft drinks. Impacts on macronutrients and dietary quality were inconclusive, though suggestive of a decrease in dietary quality. The Great Recession had greater impacts on dietary intake for disadvantaged individuals.

**Conclusions:**

The 2008 recession was associated with diverse impacts on diets. Calorie intake decreased in high income countries but increased in middle income countries. Fruit and vegetable consumption reduced, especially for more disadvantaged individuals, which may negatively affect health. Fast food, sugary products, and soft drink consumption also decreased which may confer health benefits. Implementing effective policies to mitigate adverse nutritional changes and encourage positive changes during the COVID-19 pandemic and other major economic shocks should be prioritised.

**Supplementary Information:**

The online version contains supplementary material available at 10.1186/s12966-021-01125-8.

## Background

The 2008 Great Recession had a severe impact on the global economy. Gross Domestic Products (GDP) decreased and unemployment increased in many countries, impacting industries, communities, and individuals [1]. The recession had a global impact although impacts varied with regard to their severity and how early or late they were, with European countries affected earlier and with bigger impacts, while Asian countries were affected less [2]. The Great Recession had wide-ranging impacts on health including poorer self-rated health and increased cardiovascular and respiratory disease [3–5]. A review of the impacts of the Great Recession on children also suggested increases in infant and child mortality in some countries and in perceived health and health-related quality of life [6]. The recession may have greater impacted people of lower socio-economic position (SEP), widening inequalities [5, 6].

There are various ways in which the Great Recession may have affected the food environment. Food prices generally increased over the recession due to inflation and food companies changing their market strategies to increase price per quantity of foods and package content [7, 8]. Price-off promotions on products – particularly on processed foods - also increased during the recession [9, 10]. These changes happened alongside households experiencing a reduction in resources [11]. This may have decreased food expenditure and the affordability of healthy food items, especially in low SEP groups [12–14]. For example, a study in Chicago compared low income areas to more affluent areas and found that access to healthy food worsened in low income areas [15]. There is conflicting evidence regarding impacts on overweight and obesity but available data are suggestive of a potential increase, particularly for low SEP individuals [16, 17].

Evidence from previous recessions suggests that economic shocks may have differential impacts on dietary intake. The 1997 Asian economic crisis likely impacted dietary intake, with decreased energy intake and changes in food consumption although findings appear inconsistent [18, 19]. The 1994 Mexican crisis appears to have negatively affected dietary intake, however, changes in food consumption varied between rural and urban areas [20]. Compared to these previous recessions, the Great Recession is notable for its duration and international reach, and severe impacts on unemployment, GDP, and public budgets [1]. It was also characterised by a slow recovery and, in Europe, a sovereign debt crisis leading to austerity measures for many countries. This occurred against a backdrop of increasing ubiquity of ultra-processed food, which means that impacts on dietary intake may have been larger compared to previous recessions due to the lower cost of these products [21]. Therefore, we hypothesise that the Great Recession had a substantive impact on dietary intake which justifies a focused and systematic examination. We aimed to systematically review the evidence on impacts of the Great Recession on children’s and adults’ dietary intakes and whether impacts were greater among low SEP groups. Given that previous evidence suggests the possibility of positive and negative impacts on diets and health, we have considered both as a potential impact of the Great Recession. As the Great Recession represents one of the largest economic shocks prior to the emergence of COVID-19, our study provides valuable insights to inform policy action to protect population health during the current pandemic.

## Methods

### Search strategy and selection criteria

We undertook a systematic review following a protocol registered on the International Prospective Register of Systematic Reviews (CRD42019135864) [22]. Inclusion criteria were as follows:

1. Population: individuals affected by the Great Recession. We had no restrictions on the setting of studies.
2. Exposure: the 2008 Great Recession, including macroeconomic indicators of the recession such as the unemployment rate.
3. Comparison: the same population before the recession, or different populations affected to different extents.
4. Outcome: any measure of dietary intake. We included energy intake, nutritional quality of diet (we included all indices retrieved in the literature such as the Healthy Eating Index (HEI) and Dietary Diversity Score), individual food item intake and macronutrient intake.
5. Only longitudinal primary quantitative research studies were included.

Studies were excluded if:

1. They were qualitative, descriptive, or cross-sectional studies undertaken at a single timepoint.
2. They were conference abstracts.
3. They were not in English. The English language restriction was applied as a component of the search and was also evaluated during eligibility screening.
4. They concerned alcohol consumption.

Search terms included “economic recession”, “Great Recession”, and “economic downturn” and “food intake”, “nutrition”, “food expenditure” and “macronutrient” (full details can be found in Additional File 1). The search strategy was developed in consultation with a research librarian. Sources included:

1. Electronic databases: MEDLINE; Embase; PsycINFO; Health Management Information Consortium (HMIC) (accessed through Ovid); Business Source Ultimate; CINAHL (both accessed through EBSCO); and Web of Science.
2. Grey Literature databases: WHOLit, OpenGrey Europe, and a manual search of sources including relevant third sector bodies.
3. Hand searching citation lists to identify additional relevant papers.

We undertook the search on the 23rd June 2020. References were imported into Endnote and screened in accordance with PRISMA guidelines [23]. Two authors (RJ & AL) independently screened the title and abstract of studies identified. Full texts of studies potentially eligible for inclusion were retrieved by the two reviewers and screened independently, with disagreements resolved by discussion.

### Data analysis

Data were extracted into a data extraction form on Microsoft Excel by RJ including study author, year, and title; funding and ethics; study design; setting; exposure assessment; data collection time points; participants’ age, gender, and other characteristics, for example being parents or in a specific age group; sample size; data collection method; outcome assessment methods; statistical methods; covariates; and key findings including differing impacts for low SEP individuals. AL also independently extracted 10% of papers and differences were resolved via discussion.

We used the Newcastle Ottawa Scale (NOS) to assess study quality and risk of bias using the following criteria: selection (representativeness of the sample, sample size, and exposure measurement), comparability (controlling for relevant confounders – one star if they stratified or adjusted for socio-economic measures and one star if they adjusted for other potential confounders such as age and sex), and outcome (outcome measurement and statistical test) [24–26]. While the Newcastle Ottawa Scale has no established thresholds, in line with previous studies we considered a score of less than five to indicate poor quality, five or six medium quality, and seven or eight high quality [27]. RJ assessed each study and AL independently conducted a quality assessment for a 10% subset of studies and resolved any differences via discussion.

We assessed outcome measures, exposures, and populations, and structured our review by outcome. We conducted a meta-analysis although this was only possible for studies reporting change in daily energy intake due to the heterogeneity of other outcomes. We contacted authors where data were not available, and for studies which reported stratified results (e.g. by sex), we combined these into an overall weighted estimate which we used in our meta-analysis. We converted measures to calories per adult per day and calculated mean change in calories per adult per day using a random effects model, a method for meta-analyses which allows for differences in the effect between studies [28]. We used a Forest Plot to present the findings of this analysis. Each study is represented on the y axis, and number of calories intake increased or decreased by on the x axis. Study weights and I^2^ estimates of heterogeneity are also presented in the Forest Plot. We explored country income group (high vs. middle) as a potential source of heterogeneity and present stratified analyses. We undertook a subgroup analysis of three studies with samples weighted to the US population with the same baseline and follow-up time periods to assess and address study heterogeneity, the results of which are presented in Additional File 2.

For each of the other outcomes, we used harvest plots to display and summarise study results [29]. Each study is reported as a single bar in each harvest plot, with the height of the bar indicating low, medium, or high quality according to the Newcastle Ottawa Scale. The effect direction in terms of increase, decrease, no change or mixed results, was indicated via the x axis. To assess overall direction for individual studies reporting different results within the same food group, we aggregated effects into the overall direction as we only reported each study once in each harvest plot. If subcategories (e.g. bread and pasta for sources of carbohydrates for one study) reported both increases and decreases, we reported the category as having mixed effects. If the subcategories reported no change for one or more subcategories, alongside either an increase or decrease in another subcategory, we reported that as an overall increase or decrease respectively. We also examined separately whether findings differed for low SEP individuals using socio-economic indicators from each study, such as education, income, or social class. Analyses were conducted using Stata 15.

## Results

**Fig. 1:**
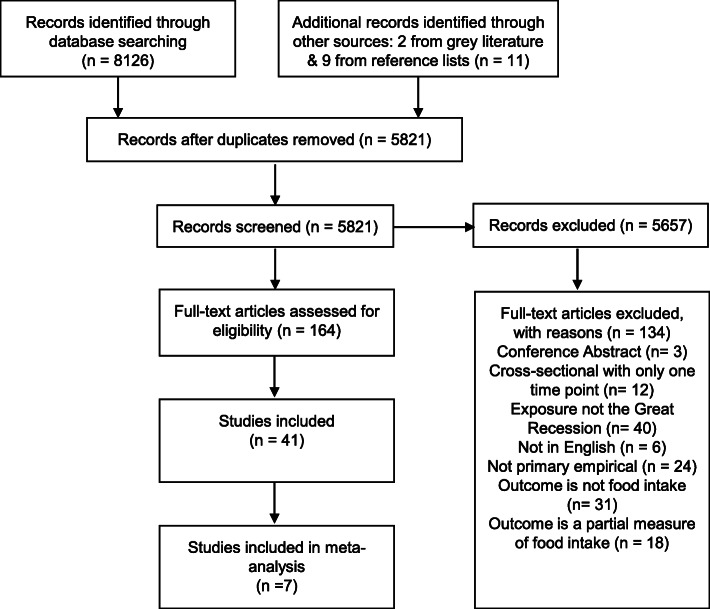
Study Selection

We identified 8126 studies, of which 2305 were duplicates, and screened all non-duplicate studies by title and abstract (see Fig. 1). We screened the full text of 164 studies. Of these, we excluded three conference abstracts, 12 cross-sectional studies, 40 studies not concerning the Great Recession, six studies not in English, 24 studies not reporting primary empirical data, 31 studies where the outcome was related to nutrition but not dietary intake, and 18 studies with partial measures of dietary intake ie. general expenditure on food at home. Thirty studies were included after full text screening, plus two from grey literature and nine from reference lists to include 41 studies overall (Fig. 1) [30–70]. Seven studies were included in our meta-analysis of daily energy intakes. Table 1 presents characteristics of the 41 included studies.

**Table 1:** Characteristics of studies included in review

Author & Year	Location	N	Exposure	Study Design and data collection years	Outcome Category	Statistical Methods	Quality^a^
Alves, 2019 [69]	Portugal	43,273	Commencement of Great Recession	Serial Cross-sectional Study: 2005–2006, 2014	Food intake	Logistic regression with dummy time variables	Medium
Antelo, 2017 [68]	Spain	28,695	Unemployment rates	Serial Cross-sectional Study: 2006, 2013	Food intake	Propensity Score Matching, Gaussian kernel methods, difference-in-difference	High
Asgeirsdottir, 2014 [67]	Iceland	9807	Commencement of Great Recession	Cohort Study: 2007, 2009	Food intake	Fixed effects regression with dummy time variables	High
Asgeirsdottir, 2016 [66]	Iceland	3238	Commencement of Great Recession	Cohort Study: 2007, 2009, 2012	Food intake	Fixed effects regression with dummy time variables	High
Bartoll, 2015 [65]	Spain	47,156	Commencement of Great Recession	Serial Cross-sectional Study: 2001, 2003–2004, 2006–2007, 2011–2012	Food intake	Before-after model (linear probability regression model) with dummy time variables	High
Bonaccio, 2014 [64]	Italy	21,001	Commencement of Great Recession	Serial Cross-sectional Study: 2005–2006, 2007–2010	Energy intake, dietary quality, food intake, macronutrients	Binomial (Poisson) regression with dummy time variables	High
Brinkman, 2010 [63]	Haiti, Nepal, Niger	5493	Changes in food prices	Serial Cross-sectional Study: 2006, 2007	Dietary quality	Ordinary Least Squares Regression with changes in food prices	Low
Colman, 2018 [61]	USA	7100	Unemployment	Cohort Study: 2008, 2010, 2012, 2014	Food intake	Fixed effects regression	High
Çırakli, 2019 [62]	Turkey	Not stated	Commencement of Great Recession	Serial Cross-sectional Study: 1994, 2001, 2008	Food intake	ARDL bounds testing and cointegration analysis	Low
Dave, 2012 [60]	USA	1,353,612	Unemployment rates	Serial Cross-sectional Study: 1990–2009	Dietary quality and food intake	Reduced form cross-equation estimates (fixed effects) of average effect of state unemployment	High
Di Pietro, 2018 [59]	Italy	189,631	Unemployment rates	Serial Cross-sectional Study: 2005–2012	Food intake	Reduced form demand function (linear probability models estimated with OLS)	High
Díaz-Méndez, 2019 [58]	Spain	50,485	Commencement of Great Recession	Serial Cross-sectional Study: 2005, 2011	Food intake	Logistic regression with dummy time variables	Medium
Duquenne, 2014 [57]	Greece	932	Change over time	Serial Cross-sectional Study: Years not stated	Food intake	Exploratory factor analysis and hierarchical cluster analysis	Medium
Filippidis, 2014 [55]	Greece	3503	Commencement of Great Recession	Serial Cross-sectional Study: 2006, 2008, 2011	Food intake	Binary logistic regression with time polynomials	Medium
Filippidis, 2017 [56]	Greece	5504	Commencement of Great Recession	Serial Cross-sectional Study: 2006, 2008, 2010, 2011, 2015	Food intake	Interrupted Time Series analysis	Medium
Florkowski, 2012 [54]	Poland	Not stated	Commencement of Great Recession	Serial Cross-sectional Study: 2004, 2005, 2006, 2007, 2008	Food intake and macronutrients	Households average yearly expenditure	Low
Foscolou, 2017 [53]	20 Mediterranean islands	2749	Commencement of Great Recession	Serial Cross-sectional Study: 2005–2008, 2009–2015	Dietary quality	Independent samples t-test	Low
García-Mayor, 2020 [70]	Spain	72,574	Commencement of Great Recession	Serial Cross-sectional Study: 2006, 2012, 2017	Food intake	Multivariate logistic regression with dummy time variables	High
Griffith, 2016a [51]	UK	14,694	Commencement of Great Recession	Cohort Study 2005–2007, 2010–12	Energy intake, dietary quality, Food intake	Modelling price per calorie including time-varying factors	High
Griffith, 2016b [52]	UK	Not stated	Commencement of Great Recession	Serial Cross-sectional Study: 1980–2007, 2007–2013	Energy intake, dietary quality, Food intake	Backcasting including household characteristics and seasonal variation and price changes	Medium
Griffith, 2013 [50]	UK	15,850	Commencement of Great Recession	Cohort Study 2005–2007, 2008–2009, 2010–2012.	Energy intake, dietary quality, Food intake, macronutrients	Regression with dummy time variables	High
Hasan, 2019 [49]	Bangladesh	11,722	Commencement of Great Recession	Serial Cross-sectional Study: 2005, 2010	Energy intake, dietary quality, food intake	Difference-in-difference framework and Ordinary Least Squares models	High
Iannotti, 2011 [48]	Guatemala, Honduras, Nicaragua, Panama, Haiti, Ecuador, Peru	71,198	Actual vs. expected price changes	Serial Cross-sectional Study: 2006,2008	Energy intake	Quadratic Almost Ideal Demand System and Kernal Density Estimates	Low
Jofre-Bonet, 2016 [47]	UK	91,045	Unemployment rates, Great Recession commencement	Serial Cross-sectional Study: 2001–2013	Food intake	Non-linear estimation methods (Tobit and probit), reporting Average Marginal Effects	High
Kim, 2019 [46]	USA	1359	Neighbourhood indicators	Serial Cross-sectional Study: 2000–2013	Food intake	Bivariate analyses and logistic regression	High
Kotelnikova, 2017 [45]	Russia	17,645	Commencement of Great Recession	Cohort Study: 1995, 1998, 2009, 2014	Food intake	Median changes and percentage change	Medium
Kuhns, 2014 [44]	USA	100,000	Commencement of Great Recession	Cohort Study: 2004–2010	Dietary quality	Fixed effects regression	High
Marcotte-Chenard, 2019 [43]	USA	38,541	Commencement of Great Recession	Serial Cross-sectional Study: 1999–2006, 2007–2008.	Energy intake, macronutrients	Factorial ANOVAs comparing intervals	Medium
Martin-Prevel, 2012 [42]	Burkina Faso	6019	Commencement of Great Recession	Serial Cross-sectional Study: 2007, 2008	Food intake	General linear mixed model	High
Mattei, 2017 [41]	Italy	Not stated	Commencement of Great Recession	Serial Cross-sectional Study: 2000–2007, 2008–2015	Food intake	Linear regression with dummy time variables	Low
Mohseni-Cheraglou, 2016 [40]	Global (ecological)	Not stated	Currency devaluation or banking distress	Serial Cross-sectional, ecological: 1981–2007	Energy intake, macronutrients	Calculating changes in growth rates	Low
Ng, 2014 [39]	USA	81,509	Commencement of Great Recession	Serial Cross-sectional Study: 2003–2004, 2005–2006, 2007–2008, 2009–2010	Energy intake, food intake;	2-sample t tests	High
Norte, 2019 [38]	Spain	49,216	Commencement of Great Recession	Serial Cross-sectional Study: 2006–2007, 2011–2012	Dietary quality	Logistic regression with dummy time variables	Medium
Nour, 2019 [37]	Canada	281,421	Commencement of Great Recession	Serial Cross-sectional Study: 2007–2008, 2008–2009, 2009–2011, 2011–2013	Food intake	Logistic regression with dummy time variables	High
Rajmil, 2013 [36]	Spain	3982	Commencement of Great Recession	Serial Cross-sectional Study: 2006. 2010–2012	Food intake	Multiple linear regression with dummy time variables	High
Regidor, 2019 [35]	Spain	Not stated	GDP	Serial Cross-sectional Study: 2004–2007, 2008–2010, 2011–2013, 2014–2016	Food intake	Segmented linear regression models	Low
Shabnam, 2016 [34]	Pakistan	30,054	Commencement of Great Recession	Serial Cross-sectional Study: 2005–2006, 2010–2011	Energy intake, Food intake, macronutrients	Quantile regression on demand equation with dummy time variables	High
Smed, 2017 [33]	Denmark	3440	Consumer Confidence Index	Serial Cross-sectional Study: 2008–2012	Energy intake, dietary quality, Food intake, macronutrients	Fixed methods econometric methods	High
Todd, 2014 [32]	USA	9839	Commencement of Great Recession	Serial Cross-sectional Study: 2005–2006, 2007–2008, 2009–2010	Dietary quality, Food intake, macronutrients	Multivariate linear regression models with Ordinary Least Squares	Medium
Todd, 2017 [31]	USA	17,326	Commencement of Great Recession	Serial Cross-sectional Study: 2005–2006, 2007–2008, 2009–2010, 2013–2014	Dietary quality, Food intake, macronutrients	Multivariate linear regression models with Ordinary Least Squares	High
Yang, 2019 [30]	USA	Not stated	Commencement of Great Recession	Serial Cross-sectional Study: 1998–2016	Food intake	Bai and Perron test, Time-Varying AIDS and Iterated Seemingly Unrelated Regression	Medium

^a^Assessed using the Newcastle Ottawa Scale

Studies with a total of 2.6 million people from 25 countries were included: 12 high income, nine middle income, and four low income [71]. Studies were heterogeneous regarding exposures, methods, and locations. Outcomes were also heterogeneous, but broadly fell into four categories:

1. energy intake (10 studies),
2. dietary quality (11 studies),
3. food intake (34 studies),
4. macronutrients (13 studies).

### Study quality and characteristics

The majority of studies were of high or medium quality: twenty-two studies (54%) were high quality, eleven (27%) medium quality, and eight (19%) low quality. The low quality studies tended not to have representative samples, clearly stated sample size, appropriate statistical tests nor adjustment for confounders. Only four studies investigated the impact of the Great Recession on children’s dietary intake [36, 39, 50, 51]. Most studies used individual-level data from pre-existing, nationally representative surveys, except for one study which used ecological data on calorie and protein intake per capita and currency movements [40]. Thirty-one studies were serial cross-sectional [30–32, 34–39, 41–43, 46–49, 52–56, 58–60, 62–65, 68–70] and eight were cohort studies [33, 44, 45, 50, 51, 61, 66, 67]. Baseline data were generally collected between 2005 and 2006 and follow-up data between 2007 and 2010, though overall the studies’ data collection years ranged from 1981 to 2017. Thirteen studies (29%) used macroeconomic measures as the exposure such as unemployment rates, Consumer Confidence Index, and neighbourhood characteristics [33, 35, 40, 45–48, 59–63, 68]. Twenty-nine studies (71%) used commencement of the Great Recession as the exposure – this was the most common exposure measure for all four outcome categories. Most used regression methods with dummy time variables [31, 32, 34, 36–38, 41, 44, 50, 55, 58, 64–67, 69, 70]. Other methods included Difference-in Difference, t-tests, ANOVAs, and time-varying Almost Ideal Demand System and Bai Perron tests [30, 42, 43, 49, 51–53, 57, 58]. Most studies adjusted for covariates including age, sex, education and socio-economic status – this was taken into account through the study quality assessment and we examined adjusted results where possible. An additional file gives more detail on the studies (see Additional File 3). Key findings are summarised in Table 2.

**Table 2:** Summary of Study Findings by Outcome

Author & Year	Exposure	Main results (only statistically significant findings described; see Additional File 2 for full details)				
		Energy Intake	Dietary Quality	Food Intake	Macronutrient intake	Socio-economic differences and impacts on children
Alves, 2019 [69]	Commencement of Great Recession	n/a	n/a	Soup, fish, fruits and vegetables significantly decreased; legumes significantly increased.	n/a	Legumes increased only among the low and medium educated; soup intake decreased only among the least educated, fish decreased only amongst those with medium education.
Antelo, 2017 [68]	Unemployment rates	n/a	n/a	Unemployment was associated with a decrease in expenditure on bread, cereals, rice and pasta; meat; fish; milk, cheese and eggs; fruits; vegetables, pulses, potatoes and other root crops; and sugar, jam, honey, chocolate, sweets and ice cream.	n/a	n/a
Asgeirsdottir, 2014 [67]	Commencement of Great Recession	n/a	n/a	Daily sugared soft drink, daily sweets, weekly fast food, daily fruit and daily vegetables decreased	n/a	n/a
Asgeirsdottir, 2016 [66]	Commencement of Great Recession	n/a	n/a	Daily sugared soft drink, daily sweets, weekly fast food, daily fruit and decreased.	n/a	n/a
Bartoll, 2015 [65]	Commencement of Great Recession	n/a	n/a	Reduction in fruits, vegetables, meats, and cold meats.	n/a	Vegetable consumption decreased only for women without a qualification, fruit decreased most for the unemployed and those with lowest education. Meat consumption decreased the most among men and women with the lowest education.
Bonaccio, 2014 [64]	Commencement of Great Recession	Calorie intake decreased.	Adherence to Mediterranean Diet and antioxidant score decreased.	Animal proteins and fats increased, vegetarian proteins and fats decreased.	Carbohydrate intake and fibre intake decreased. Protein, fats, and saturated fats increased.	Mediterranean Diet adherence highest in those with higher wealth score and education.
Brinkman, 2010 [63]	Changes in food prices	n/a	Diet quality and diversity decreased in all three countries.	n/a	n/a	n/a
Colman, 2018 [61]	Unemployment	n/a	n/a	Becoming nonemployed and unemployed was associated with decreased consumption of fast food.	n/a	n/a
Çırakli 2019 [62]	Commencement of Great Recession	n/a	n/a	Annual per capita vegetable and fruit consumption increased.	Sugar consumption increased.	n/a
Dave, 2012 [60]	Unemployment rates	n/a	Higher state unemployment was associated with decreased dietary quality.	Higher state unemployment was associated with decreased consumption of fruits, fruit juice, carrots and green salad and vegetables, as well as significantly increased snacks.	n/a	Lower education was associated with lower consumption of fruits, fruit juice, carrots, green salad and vegetables. Lower education was associated with higher consumption of snacks, hamburgers, hot dogs, French fries, fried chicken and doughnuts.
Di Pietro, 2018 [59]	Unemployment rates	n/a	n/a	Higher unemployment rate was associated with decreased probability of consuming at least 5 portions of fruit and vegetables per day and increased probability of eating snacks high in salt every day.	n/a	n/a
Díaz-Méndez 2019 [58]	Commencement of Great Recession	n/a	n/a	Fruit, vegetables, meat, fish, and sweets consumption decreased.	n/a	Lower social class and education level, and higher unemployment, associated with lower fruit consumption. Unemployment associated with lower fish consumption.
Duquenne, 2014 [57]	Change over time	n/a	n/a	Recession had limited impact on consumption of pasta, potatoes, olive oil, rice, bread, vegetables, milk, and fruits (component 1). There was a bigger impact on beef, sheep and goat, pork, cold cuts, chicken, fish, sweets, cheese and feta consumption, with more than 60% of households changing their behaviour (component 2).	n/a	Generally those more affected by Great Recession had a lower income and greater decrease in monthly income, and higher unemployment.
Filippidis, 2014 [55]	Commencement of Great Recession	n/a	n/a	Significant decrease in consuming five portions of fruits and vegetables per day.		Decrease in consuming 5 portions of fruits & vegetables greater in those of lower socio-economic status.
Filippidis, 2017 [56]	Commencement of Great Recession	n/a	n/a	No significant change in low fruit and vegetable consumption (two or less portions).	n/a	n/a
Florkowski, 2012 [54]	Commencement of Great Recession	n/a	n/a	Expenditure share from pasta, bread, seafood, offal, barley, pork, chicken, milk, farmers’ cheese, hard cheese, eggs, margarine, vegetable oil, animal fats, citrus and apples increased. Expenditure share from freshwater fish, potatoes and sugar decreased.	Sugar consumption decreased.	Generally those below average income spent less on the different food types. Observed changes were the same but with a lower start and end level of consumption.
Foscolou, 2017 [53]	Commencement of Great Recession	n/a	Adherence to Mediterranean Diet decreased after 2009.	n/a	n/a	n/a
García-Mayor, 2020 [70]	Commencement of Great Recession	n/a	n/a	Daily fruit, vegetables, pastries and sweets, and sugar-sweetened beverages decreased.	n/a	Lower fruit and vegetable intake for those of lower social class, also a greater decrease in pastries and sweets of those of lower SEP.
Griffith, 2016a [51]	Commencement of Great Recession	Calories purchased and energy density decreased.	Healthy Eating (HEI) score increased.	Share of calories from fruit, grains, poultry and fish, prepared sweets and desserts, and confectionary increased; vegetables, red meat and nuts, fats and oils, eating out and fast food and drinks decreased.	Carbohydrates, sugar, fibre and saturated fats increased and protein and salt decreased.	Middle income individuals decreased their calories purchased the most. Working low income individuals improved their HEI score the most. Households with children reduced expenditure and calories the most; households with pre-school children increased their HEI score the most.
Griffith, 2016b [52]	Commencement of Great Recession	n/a	n/a	Fruit and vegetables, dairy, meat, fish, eating out and fast food, soft drink and confectionary consumption decreased.	n/a	n/a
Griffith, 2013 [50]	Commencement of Great Recession	Calorie density increased.	Dietary quality decreased over the recession.	Decrease in calorie share from fruit and vegetables.	Saturated fat, sugar, and protein consumption increased.	Calorie density increased most in single parents and families of two adults with young children. HEI was lowest for single pensioners. Households with children increased protein consumption the most but decreased calories from vegetables the most.
Hasan, 2019 [49]	Commencement of Great Recession	Calorie intake per day increased over the recession.	Household Dietary Diversity Score and number of food groups consumed increased while Food Consumption Score decreased.	Consumed rice and calories from non-rice grain, pulses, high value and low value pulses, fruits, proteins, low value fish and other items increased, while calorie intake from high value fish decreased.	No change in calorie intake from protein.	Higher education was associated with higher Household Dietary Diversity Score and Food Consumption Score; lower calories from rice and grain and higher calories from other grains and protein.
Iannotti, 2011 [48]	Actual vs. expected price changes	Calories decreased in general.	n/a	n/a	n/a	Considerable differences in by wealth score; lower wealth associated with fewer calories consumed.
Jofre-Bonet, 2016 [47]	Unemployment rates, commencement of Great Recession	n/a	n/a	Vegetable consumption increased and fruit consumption decreased.	n/a	n/a
Kim, 2019 [46]	Local indicators	n/a	n/a	Decrease in median household income was associated with decreased fruit and vegetable availability in the home.	n/a	Higher socio-economic status associated with higher availability of fruit and vegetables in the home.
Kotelnikova, 2017 [45]	Commencement of Great Recession	n/a	n/a	Food expenditure in the previous week on bread, cereals, and canned food; fresh vegetables; fresh meat and fish; milk and dairy products; and berries and other fresh fruits decreased.	n/a	n/a
Kuhns, 2014 [44]	Commencement of Great Recession	n/a	USDA score for dietary quality increased.	n/a	n/a	n/a
Marcotte-Chenard, 2019 [43]	Commencement of Great Recession	Calories decreased for men and women.	n/a	n/a	Protein, carbohydrate, sodium and sugar intake decreased in men and women. Fats significantly decreased in women only.	n/a
Martin-Prevel, 2012 [42]	Commencement of Great Recession	n/a	n/a	Tubers/roots, green leafy vegetables, eggs and vitamin A (VA)-rich oil (red palm oil) increased. VA -rich vegetables and tubers, other vegetables, VA-rich fruits, other fruits, offal, meat, fish, legumes/ nuts/ seeds, milk/ dairy products and oils/fats decreased.	Sugar consumption decreased.	n/a
Mattei, 2017 [41]	Commencement of Great Recession	n/a	n/a	No significant impact on foods assessed	n/a	n/a
Mohseni-Cheraglou, 2016 [40]	Currency devaluation or banking distress	Growth rates for calorie intake per day decrease during economic crises with or without recessions.	n/a	n/a	Growth rates for protein intake per day decrease during economic crises with or without recessions.	n/a
Ng, 2014 [39]	Commencement of Great Recession	Mean calories consumed per day decreased in adults and children.	n/a	Increase in unemployment rate associated with increased calories from consumer packaged goods and beverages.	n/a	**n/a**
Norte, 2019 [38]	Commencement of Great Recession	n/a	Odds of poor diet increased for the less affluent.	n/a	n/a	Increased odds of poor diet higher for those in unskilled work or with lower education.
Nour, 2019 [37]	Commencement of Great Recession	n/a	n/a	Recession not significantly associated with fruit and vegetable consumption.	n/a	n/a
Rajmil, 2013 [36]	Commencement of Great Recession	n/a	n/a	Junk food consumption decreased for families with maternal primary education level.	n/a	Junk food consumption decreased more for families with lower maternal primary education level.
Regidor, 2019 [35]	GDP	n/a	n/a	Fruit and vegetable consumption increased.	n/a	n/a
Shabnam, 2016 [34]	Commencement of Great Recession	n/a	n/a	Vegetable, wheat and wheat flour, rice, milk and milk products, legumes, fats and oils and sugar increased. Fruit consumption decreased.	Price elasticity for carbohydrates, fats, and proteins decreased.	Greater impact on low income families.
Smed, 2017 [33]	Consumer Confidence Index (CCI)	n/a	n/a	Canned and processed fish, fresh fish, fresh fruit, poultry, processed meat, sliced meat, fats, cheese, dairy and sugar products significantly increase with increased CCI (so decreased during recession). Pork and snacks significant decrease with increased CCI (so increased during recession).	Total fat, saturated fats, and protein increase with increased CCI. Added sugar and carbohydrates decreased with increased CCI.	n/a
Todd, 2014 [32]	Commencement of Great Recession	n/a	More likely to rate dietary quality as excellent or very good in 2009–2010 compared to 2007–2008.	No significant change in total snacks consumed but did find a significant decrease in snacks eaten away from home. Decrease in calories from fast food.	Percentage calories from fat and saturated fat, fibre intake, and cholesterol intake decreased.	n/a
Todd, 2017 [31]	Commencement of Great Recession	n/a	n/a	No significant change in total snacks consumed but did find a significant decrease in snacks eaten away from home. Decrease in calories from fast food.	Saturated fat, fibre, and cholesterol intake decreased.	n/a
Yang, 2019 [30]	Commencement of Great Recession	n/a	n/a	Decreases in beef and pork expenditure, with income differences. Increase in eggs and no change in dried beans.	n/a	For lower incomes households, pork expenditure decreased over time while fish, seafood, and dairy expenditure increased over time. For higher income households, beef expenditure decreased while eggs and dairy products increased over time. For middle income households, bean expenditure increased over time.

### Energy intake

Ten studies assessed changes in energy intake, generally located in high and middle income countries [31–33, 39, 40, 43, 48, 49, 51, 64]. Seven used daily calorie intake as the outcome [32, 39, 43, 48, 49, 51, 64], while one used monthly intake in kJ [33] and one examined changes in growth rates using non-individual data [40]. For one study based in seven different countries in Latin America, only data from Guatemala were available from the authors [48]. We were unable to obtain standard deviations for a UK study so this was omitted from the meta-analysis, as was a Danish study assessing how Consumer Confidence Index affects energy intake [33, 51]. Seven studies were included in our random effects meta-analysis [31, 32, 39, 43, 48, 49, 64] – four of these studies were high quality [31, 39, 49, 64]. All had representative samples, adequate sample-size, adequate ascertainment of exposure, adequate ascertainment of outcome, and samples were comparable with regard to analysis and controlling for confounders. However, not all scored highly for appropriateness and description of statistical test. Two were medium quality [32, 43] and one low quality due to no reference to the representativeness of the sample, inadequate ascertainment of the exposure, and inadequate reporting of the statistical test [48]. All studies were serial cross-sectional and the time periods in which baseline and follow-up data-collection occurred ranged from 1999 to 2006 and 2007 to 2010 respectively.

**Fig. 2:**
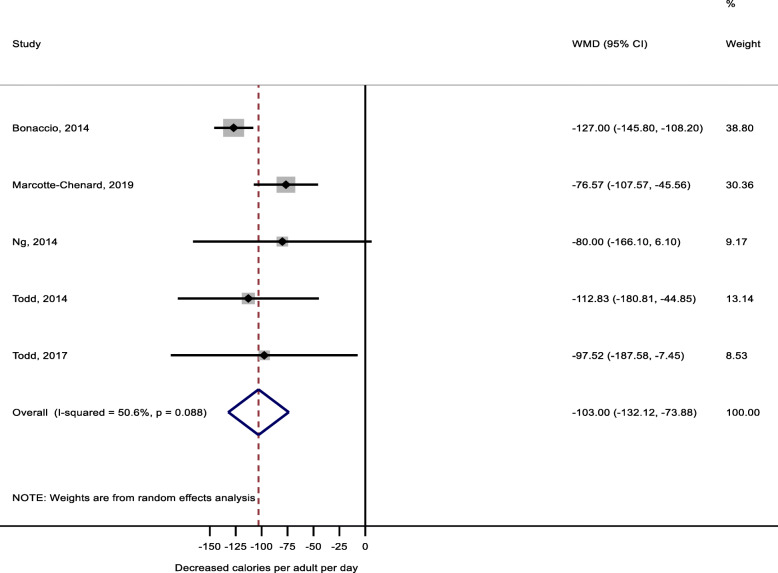
Forest Plot for change in total energy intake per adult per day in calories in high income countries

**Fig. 3:**
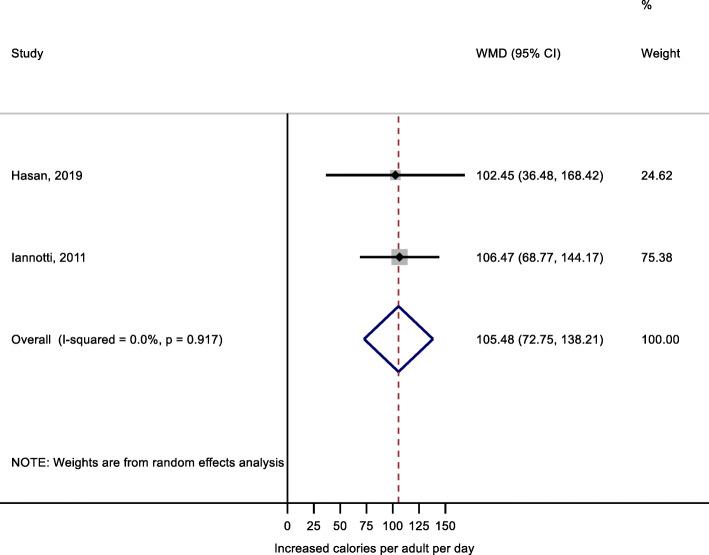
Forest Plot for change in total energy intake per adult per day in calories in middle income countries

We found that overall, energy intake decreased by 39.9 cal per adult equivalent per day (95% Confidence Interval: − 119.9, 40.2) over the Great Recession. The I^2^ statistic for heterogeneity was 95.9%, suggesting considerable heterogeneity. When only high-income countries (USA and Italy) [31, 32, 39, 43, 64] were included in the meta-analysis (three of which are high quality and two medium), energy intake decreased by 103.0 cal per adult equivalent per day (95% Confidence Interval: − 132.1, − 73.9) with a lower I^2^ statistic of 50.6% (Fig. 2). When the meta-analysis was run for middle-income countries (Guatemala and Pakistan, the former low quality and the latter high quality [48, 49]), energy intake increased by 105.5 cal per adult per day (95% confidence Interval: 72.8, 138.2), with an I^2^ statistic of 0.0%, indicating very low heterogeneity (Fig. 3).

The decrease in energy intake in high-income countries was supported by studies not included in the meta-analysis. A Danish study also reported that decreasing Consumer Confidence Index as a proxy for the recession was associated with lower monthly energy intake [33]. Similarly, a UK study reported a decrease of 26 cal per day [51]. The only study investigating children’s daily energy intake suggested that children experienced larger decreases than adults in the USA (210 cal per capita per day) [39]. Two studies observed a decrease in food expenditure alongside the decrease in calories [33, 51]. Decreases in calories were also accompanied by decreases in consumption of several different food groups and macronutrients, although the types of foods decreasing were not consistent (see below for further details) [33, 43, 51, 64].

### Dietary quality

**Fig. 4:**
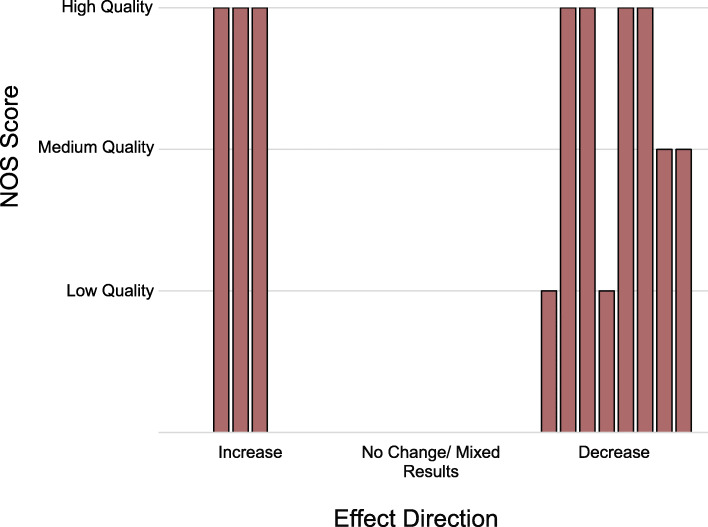
Harvest plot for studies assessing dietary quality. Each bar represents a single study, with the height of the bar representing study quality via the Newcastle Ottawa Scale. The x axis indicates effect direction

Eleven studies examined the impact of the recession on dietary quality using indices of dietary quality and diversity [32, 38, 42, 44, 49–51, 53, 60, 63, 64]. These studies were generally located in high and low income countries. Outcomes were heterogeneous, including Dietary Diversity Score [42, 49], Food Consumption Score [49, 63], and HEI [50, 51]. Eight studies (four of which were of high quality) reported negative impacts [32, 38, 42, 50, 53, 60, 63, 64] and three (all high quality) reported positive impacts [44, 49, 51] on dietary indices (Fig. 4). Overall, findings suggest that there may have been a decrease in dietary quality over the Great Recession, although given the three high quality studies reporting an increase in dietary quality, this cannot be ruled out. There was little consistency across measures, for example Food Consumption Score decreased in Haiti but did not significantly change in Bangladesh [49, 63]. There was also little consistency within countries. For example, in the UK, one study found that HEI improved by 1.5% over the recession, however, this increase masked a shift away from vegetables, grains, milk, and meat which was offset by a lower calorie share of saturated fat and lower salt consumption [51]. An earlier paper using a different dataset over the same time period found a similar decrease in saturated fat intake and fruit and vegetable consumption, but a 1% decrease in HEI [50]. Antioxidant consumption score decreased alongside a decrease in Mediterranean Diet score between 2005 and 2006 and 2007–2010 in Italy, suggesting a decrease in dietary quality [64].

### Food intake

Thirty-four studies reported on food intake, generally located in high and middle income countries [30–37, 39, 41, 42, 45–47, 49–52, 54–62, 64–70]. The most common outcome was consumption of a food group or an amount of food as a binary outcome per day/ week [37, 41, 55, 56, 58, 59, 65, 69, 70]. Other commonly used outcome measures included expenditure or frequency of consumption in a specified time period [33, 36, 45, 47, 52, 54, 60, 61, 66–68] or share of calories or calorie intake from food groups [31, 32, 39, 49, 51, 52]. Overall, consumption of fruits and vegetables, meat and fish, fast food, sugary products, and soft drinks decreased during the recession, with egg and legume consumption increasing and sources of carbohydrate consumption unchanged. We found mixed results regarding intake of dairy, oils and fats, and snacks. Results were generally consistent by study quality. Harvest plots for all outcomes can be found in Additional File 4.

**Fig. 5:**
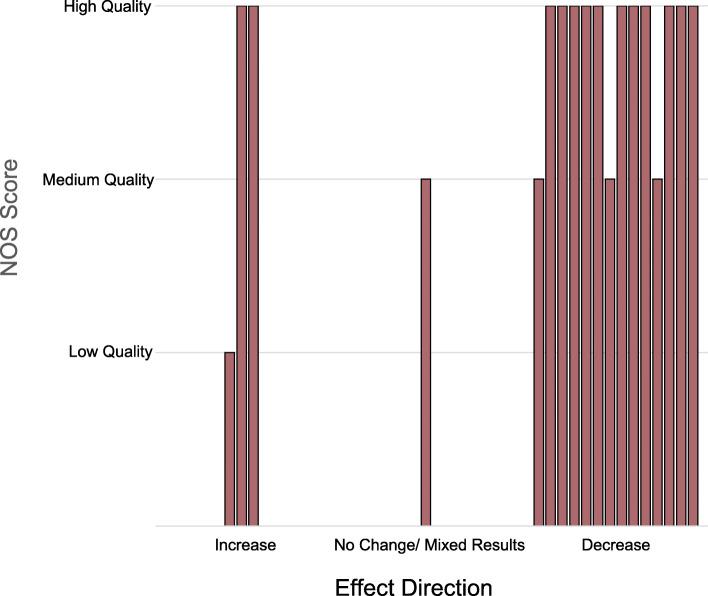
Harvest plot for studies assessing fruit intake. Each bar represents a single study, with the height of the bar representing study quality via the Newcastle Ottawa Scale. The x axis indicates effect direction

**Fig. 6:**
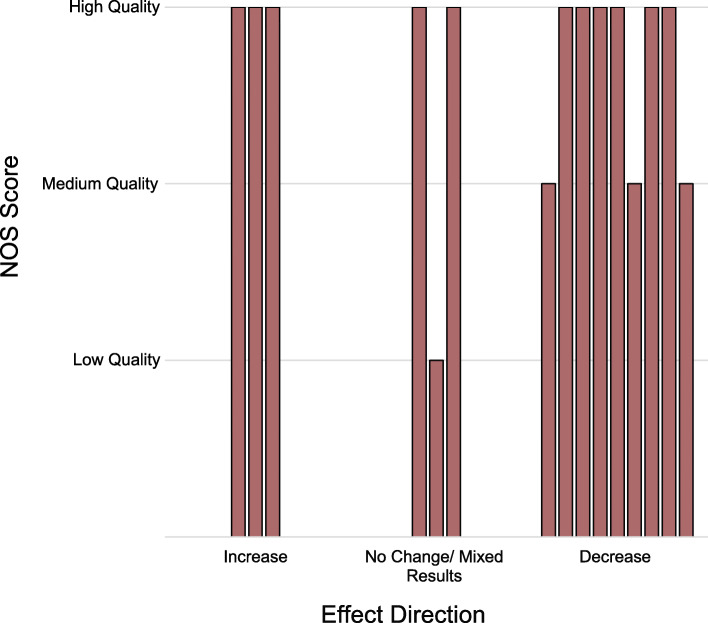
Harvest plot for studies assessing vegetable intake. Each bar represents a single study, with the height of the bar representing study quality via the Newcastle Ottawa Scale. The x axis indicates effect direction

Eight studies examined fruit and vegetable consumption combined, four of which found decreases in fruit and vegetable consumption [50, 52, 55, 59] and two found no significant impact [37, 56]. Two studies found increases in fruit and vegetable consumption, although both were of low quality [35, 62]. Fourteen of the eighteen studies on fruit intake alone found that this decreased over the Great Recession (Fig. 5) [33, 34, 42, 45–47, 58, 60, 65–70]. One reported little impact [57] and three reported increases in fruit intake [49, 51, 54]. All studies examining whether individuals consumed fruit daily reported decreases in fruit consumption [42, 47, 58, 65–67, 69, 70]. Fifteen studies investigated the impact of the Great Recession on vegetable intake (Fig. 6). Nine reported decreases in vegetable intake [45, 46, 51, 58, 60, 65, 67–69] and three reported no significant impacts [33, 41, 42]. A Spanish study observed a decrease in daily vegetable consumption that was only significant in women without an educational qualification [65]. One UK study found a 7.8% decrease in share of calories from vegetables [51] while another UK study found a small increase in portions of vegetables eaten per day in the UK [47]. A study in Spain found that the odds of eating vegetables daily increased between 2006 and 2012 [70]. In Pakistan, expenditure on vegetables increased slightly, but less than wheat and rice expenditure [34].

Five of eleven studies on sources of carbohydrates reported no change [33, 42, 45, 57, 69] and four reported differing directions of associations suggestive of within-category substitutions [49, 51, 52, 54] (Additional File 4 A1). An overall decrease [68] and increase [34] in expenditure on sources of carbohydrates was seen in one study each. Eleven studies examined dairy consumption (Additional File 4 A2) with mixed results [33, 34, 41, 42, 45, 51, 52, 54, 57, 68]. Five studies reported overall decreases [33, 42, 45, 57, 68] and two reported increases [34, 54], while one reported no change [41] and two reported mixed results [51, 52]. Patterns were inconsistent across income groups in the US [30]. Nine studies examined consumption of fats and oils (Additional File 4 A3) [33, 34, 42, 51, 52, 54, 57, 64, 68]. Only one study found that oil consumption decreased [51]; monthly purchases of fats decreased in Denmark, but not oils [33]. Expenditure on fats and oils increased in Pakistan and Poland [34, 54]. The remaining five studies found mixed or null results [42, 52, 57, 64, 68].

Sixteen studies investigated intake of sources of proteins [30, 33, 34, 41, 42, 45, 49, 51, 52, 54, 57, 58, 64, 65, 68, 69]. While most studies examined consumption of protein sources separately, share of calories from poultry and fish increased while calories from red meat and nuts decreased in the UK [51]. Expenditure on meat, poultry, and fish increased in Pakistan [34] but decreased in Russia [45]. Animal proteins per day increased but vegetarian proteins decreased in Italy [64]. Twelve studies examined meat consumption (Additional File 4 A4). Seven reported decreases (with consistent results in Spain and the UK) [42, 45, 51, 52, 58, 65, 68], one reported an increase [54], two reported no change [41, 69] and two reported mixed results [30, 33]. Eleven studies reported on fish consumption (Additional File 4 A5). Six reported decreases over the recession [33, 42, 52, 58, 68, 69], one reported an increase [30], two reported no association [41, 65] and two reported differential effects including a decrease in fish but an increase in seafood in Poland [49, 54]. Potential substitution of high cost fish for low cost fish was reported in Bangladesh [49]. Three of five studies reporting on egg consumption reported increases (Additional File 4 A6) [30, 33, 42, 52, 54]. Consumption of beans, legumes, and pulses significantly increased in five of the six studies it was investigated in [34, 42, 49, 65, 69], with one US study reporting no change [30].

Eight studies examined the impact of the recession on fast food (Additional File 4 A7) - seven reported a decrease in consumption [31, 32, 36, 52, 61, 66, 67] and one reported no change [60]. Food at restaurants, cafés, bars, bistros, fast food outlets, and takeaways decreased in the UK, but share of calories from prepared savoury foods and ready meals increased [52]. There was an increase in calories from Consumer Packaged Goods (especially for households with children) in the USA [39]. Consumer Confidence Index was not associated with processed food consumption in Denmark [33]. Six studies reported on snack consumption (Additional File 4 A8). Three found increases in snack consumption [33, 59, 60], two found mixed results suggestive of decreases [31, 32], and one found no significant change [61]. Higher area-level unemployment rate was associated with an increase in snack consumption in the USA and Italy [59, 60]. There was no significant change in total snacks consumed but a significant decrease in snacks eaten away from home in the USA [31, 32].

Twelve studies examined the Great Recession’s impact on consumption of sugary products such as desserts and confectionary (Additional File 4 A9); eight reported decreases [33, 52, 57, 58, 66–68, 70], two reported increases [51, 69], and two reported no significant change [60, 65]. Calories from confectionary, soft drinks, sugary products, and preserves consumed in and out of the home decreased in one UK study [52]. However, another UK study by the same authors found that share of calories from prepared sweets increased [51]. Eight high quality studies examined the impact of the Great Recession on non-alcoholic beverage consumption (Additional File 4 A10), primarily concerning soft drinks and fruit juice [33, 39, 51, 60, 61, 66, 67, 70]. All reported decreases in consumption of beverages, although for two this decrease was not significant.

### Macronutrients

Thirteen studies – from high, middle, and low income countries – assessed consumption of macronutrients with generally mixed results [31–34, 40, 42, 43, 49–51, 54, 62, 64]. Macronutrient outcomes included calories (and share of calories) from macronutrients [31, 32, 49, 51], grams per day/month or grams per 100 g [31–33, 43, 50], and price elasticity [34]. For sugar consumption, measures also included percentage change in sugar consumption, budget share, and expenditure on sugar [33, 34, 42, 43, 50, 51, 54, 62].

Four studies reported on carbohydrate consumption (Additional File 4 A11) - two reported decreases ranging from 3.3 g to 16 g per day [43, 64], one reported a slight increase [51], and one reported no significant change [33]. Eight studies examined sugar intake (Additional File 4 A12), although for many of these it was unclear whether they were examining added sugar or general dietary sugar intake [33, 34, 42, 43, 50, 51, 54, 62]. Four found that sugar intake increased [34, 50, 52, 62] while three studies identified decreases [42, 43, 54]. For example, UK households increased their overall sugar intake by 0.20 g per 100 g, but for households with children this increase was by 0.44 g per 100 g increase (an increase of ~ 6 g per day) [50].

Seven studies examined protein intakes (Additional File 3 A13) and four reported decreases [33, 40, 43, 51], two reported small increases [50, 64], and one reported no change [49]. In one US study, protein intakes significantly decreased (by ~ 4 g) only in men [43]. Four studies reported on total fat consumption and saturated fat consumption (Additional File 3 A14 and A15) [31, 33, 43, 64]. Directions of patterns were consistent within studies but different between studies, with increases [64], decreases [33], and no significant changes reported [31, 32]. In the UK, the share of calories from unsaturated fats increased slightly alongside a decrease in share of calories from saturated fats in one study, although in a separate study there was a small increase in saturated fat consumption, particularly in pensioners [50, 51]. Total daily fat intakes decreased by ~ 3 g in women only in the USA [43]. Additionally, there was no significant change in cholesterol consumption in the USA [31, 32]. Three of five studies reported decreases in dietary fibre over the Great Recession [31, 32, 64]; one reported no significant change [33] and one UK study observed an increase [51] (Additional File 4 A16).

### Inequalities

Eighteen studies examined inequalities including high, middle, and low income nations and found that the recession led to greater changes in dietary intake for low SEP individuals [30, 34, 36, 38, 46, 48–51, 54, 55, 57, 58, 60, 64, 65, 69, 70]. Of these, ten were high quality [34, 36, 46, 49–51, 60, 64, 65, 70], six medium quality [30, 38, 55, 57, 58, 69], and two low quality [48, 54]. Two studies assessed calorie intakes, four dietary quality, twelve food intake, and one macronutrient intake. Inequalities were operationalised in terms of education, income/wealth, social class, or job type. Low SEP individuals consistently had greater decreases in fruit and vegetable intake [46, 54, 55, 58, 60, 65, 70] while patterns of meat and fish consumption were less clear but generally suggestive of inequalities [30, 58, 65, 69]. Results were more mixed for fast food consumption [36, 60]. The Great Recession also seems to have increased already-existing inequalities in dietary quality [38, 49, 64], except for one UK study which found that lower income households improved their HEI score the most [51]. Only four studies investigated the impact on children’s dietary intake so we were unable to make meaningful conclusions regarding this subgroup [36, 39, 50, 51].

## Discussion

Our systematic review suggests that the Great Recession impacted dietary intake in diverse ways. Our meta-analysis found a decrease in daily calorie intake in high income countries and an increase in daily calorie intake in middle income countries. We report decreases in fruit and vegetable intake which may have large negative population health impacts. These impacts were larger among low SEP people. We also observed decreases in fast food, sugary products, and soft drink consumption which may confer benefits to health.

Findings from our meta-analysis indicate a decrease of 103 cal per adult equivalent per day in high income countries and an increase in 106 cal per adult per day in middle income countries. More research is needed regarding impacts in middle income countries, as only two studies were included in our meta-analysis of middle income countries. Although only based on five studies, the decrease of 103 cal in high income countries which we have described is consistent with other evidence suggesting reduced food expenditure during the recession in high income settings, reflecting a tightening of household budgets [12, 14, 33, 51]. These results in high income countries should be treated with caution, but may reflect a shift in foods consumed. Fast food, sugary products, and soft drinks consumption decreased in most high-income countries observed, which may have contributed to the decrease in calorie intake we describe. These decreases may confer benefits for health in high income countries [72, 73].

However, another response to the recession may be purchasing different groceries or altering the nutritional characteristics of foods, which may result in changes to calorie intakes and differential impacts on nutrition [51]. The decreases in fruit and vegetable consumption we describe may be reflective of switching to cheaper diets in order to save money, as diets high in fruits and vegetables tend to be more expensive [74]. Small changes in fruit and vegetable consumption can significantly affect risk of coronary heart disease and overall mortality [75] and thus, reductions in intake of these foods may have large negative impacts on population health [76]. We have also found that the 2008 Great Recession may have been associated with poorer dietary quality, which further suggests that changes in food intakes are translating to poorer quality diet – however, our review found studies on the impact on dietary quality to be inconclusive due to some high quality studies suggesting a positive impact. Consistent with previous studies, we found that the Great Recession may have widened already existing inequalities, especially in relation to fruit and vegetable consumption [77]. This further supports a role for dietary costs as a mechanism for the recession’s impacts, as low SEP groups tend to select cheaper, nutrient-poor diets [78]. Our review supports previous evidence regarding the impact of the recession on widening health inequalities within countries [5, 6].

Our review has several strengths. We focused on longitudinal studies, used a variety of dietary measures as outcomes, and employed meta-analysis where this was feasible. We applied numerous search terms to seven databases and a range of grey literature sources to ensure that our search of the literature was comprehensive and examined impacts globally. However, our systematic review has some limitations which should be considered when interpreting findings. First, studies were heterogeneous in terms of exposures, methods, outcomes, and results. The studies included in the meta-analysis were heterogeneous in terms of time periods and whether they adjusted or weighted for age and sex. We used a random effects model for meta-analysis and undertook a subgroup analysis of three studies with the same time period in the USA, with each study weighting data to be nationally representative. The results did not differ considerably from the full high-income countries meta-analysis (see Additional File 2). Second, only studies in English were included in this review, which may have led to some research being excluded. Third, many of the included studies used the year when the Great Recession commenced as their exposure rather than macroeconomic measures, which may bias findings towards the null. However, both groups of studies had broadly similar findings suggesting that use of different exposure measures did not have a substantive impact.

More robust research is needed to overcome the issues relating to the heterogeneity of the literature that we have encountered and establish causal links between recessions and dietary intake. A large range of measures of dietary intake were identified in our study. The field of public health nutrition could benefit from initiatives to agree on core outcomes for measurement in future studies. Additionally, more research on the impact of recessions on food intakes and dietary quality is especially needed as we had insufficient study data to quantify impacts on these outcomes. We also recommend further research into the pathways through which recessions may positively or negatively impact dietary intake, particularly the role of unemployment, changes in income, and food price increases. Furthermore, only four studies investigated the impact of the recession on children’s diets which remains an important avenue for future work [36, 39, 50, 51].

## Conclusions

Our systematic review suggests that the Great Recession had a diverse impact on dietary intake, with reductions in daily energy intake in high income countries and fruit and vegetable consumption. These reductions are likely to have substantial impacts on population health. Furthermore, it seems that the Great Recession disproportionately affected dietary intake among low SEP individuals, and thus may contribute to widening health inequalities. However, we also observed decreases in fast food, sugary products, and soft drink consumption, which may confer benefits to health, suggesting that the Great Recession impacted diets in diverse ways. Policy-makers should consider interventions to ensure healthfulness of diets during recessions, particularly for low SEP individuals. With the COVID-19 pandemic initiating a new global recession, we would urge international and national policy-makers to consider strategies to mitigate potential impacts of recessions on dietary intake, nutrition, and health for the whole population but particularly those of low SEP.

## Supplementary Information

**Additional file 1.**

**Additional file 2.**

**Additional file 3.**

**Additional file 4.**

## Data Availability

The dataset of energy intakes analysed during the current study are available from the corresponding author on reasonable request. All papers included in our review are cited.

## Abbreviations

GDP: Gross Domestic Product
SEP: socio-economic position
NOS: Newcastle-Ottawa Scale
HEI: Healthy Eating Index
