# Supplementary material for: Impacts of the 2008 Great Recession on dietary intake: a systematic review and meta-analysis
Source: Int J Behav Nutr Phys Act. 2021 Apr 29;18:57. doi: 10.1186/s12966-021-01125-8 (PMC8084260; doi:10.1186/s12966-021-01125-8)
Supplement: Supplementary file 1 — Additional file 1. [file 12966_2021_1125_MOESM1_ESM.pdf]

Additional File 1: Search Strategy

Component	Search Terms
Exposure	"economic recession" or "great recession" or "economic crisis" or "economic collapse*" or "economic downturn*" or "economic insecur*" or "financial crisis" or "credit crunch" or "foreclos*"<br>Database-appropriate MeSH terms were used.
Outcome	"food*" or "food intake" or "food expenditure" or "food spending" or "food consumption" or "food purchas*" or "fruit" or "vegetable" or "sweet*" or "sugar-sweetened beverage*" or "dietary" or "macronutrient" or "micronutrient" or "eating"<br>Database-appropriate MeSH terms were used.
Other factors	Non-English papers were excluded.<br>Papers were included if they were primary empirical, longitudinal studies.<br>Search was limited to humans
