# Supplementary material for: Impacts of the 2008 Great Recession on dietary intake: a systematic review and meta-analysis
Source: Int J Behav Nutr Phys Act. 2021 Apr 29;18:57. doi: 10.1186/s12966-021-01125-8 (PMC8084260; doi:10.1186/s12966-021-01125-8)

**Additional File 2**

We undertook a subgroup analysis of studies by Ng (2014), Todd (2014) and Todd (2017). These studies were conducted with the same baseline (2005-2006) and follow-up (2007-2008) periods in the USA and were weighted for the population. This meta-analysis gave an $I^2$ value of 0.0%, indicating very low heterogeneity. The mean difference of calories per adult per day was - 99.5.

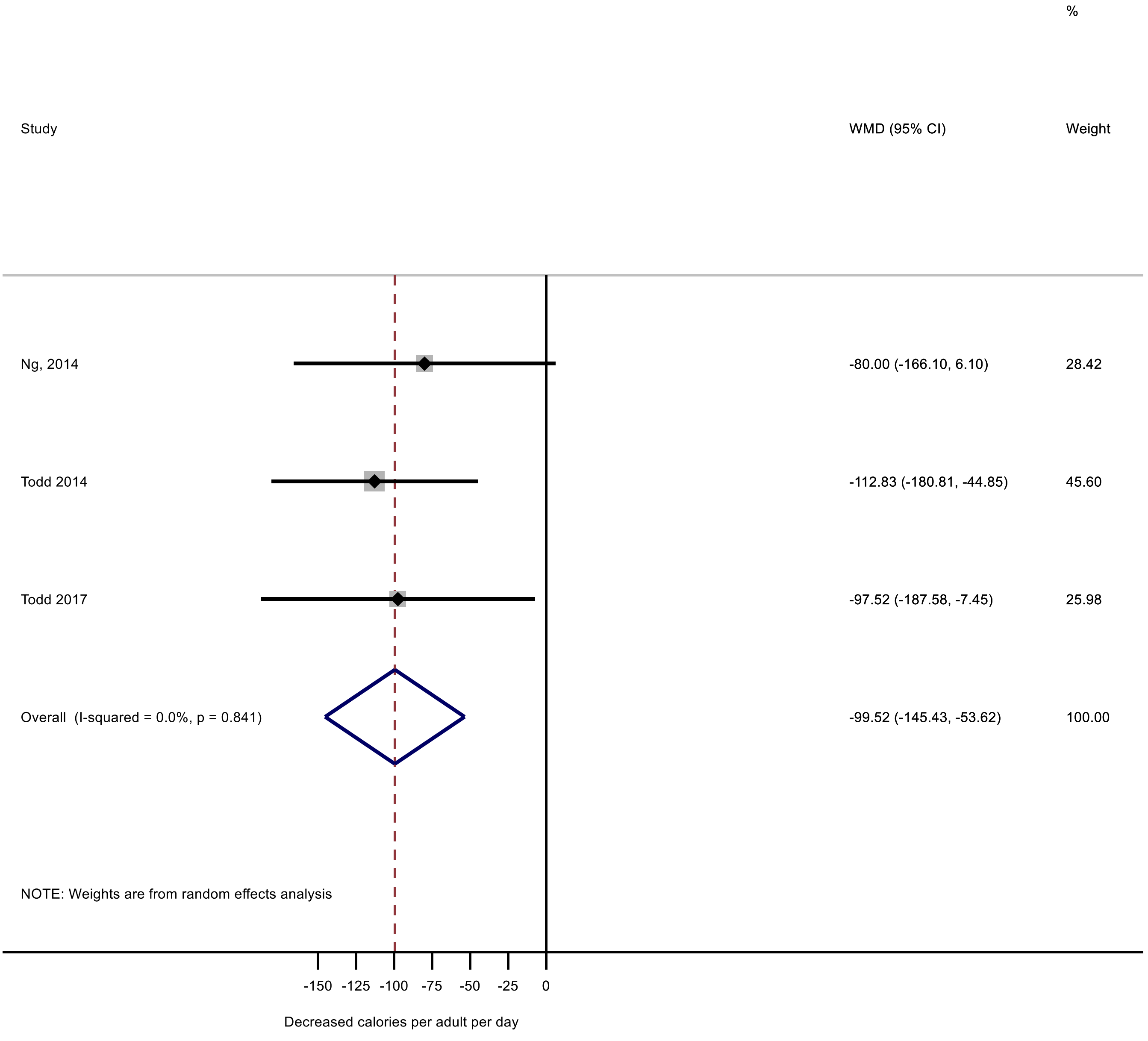

Supplement: Supplementary file 2 — Additional file 2. [file 12966_2021_1125_MOESM2_ESM.pdf]
