# Supplementary material for: Impacts of the 2008 Great Recession on dietary intake: a systematic review and meta-analysis
Source: Int J Behav Nutr Phys Act. 2021 Apr 29;18:57. doi: 10.1186/s12966-021-01125-8 (PMC8084260; doi:10.1186/s12966-021-01125-8)
Supplement: Supplementary file 3 — Additional file 3. [file 12966_2021_1125_MOESM3_ESM.pdf]

Additional File 3: Characteristics of study by 4 outcome categories:

Author & year (incl. citation)	Location (national and local if relevant)	Participants (n, population, age, dataset used, other character-istics)	Design (incl. stats methods)	Exposure incl. year(s)	Main outcomes	Main findings	Newcastle Ottawa Scale Score
Energy Intake							
Bonaccio et al, 2014 Ref:	National: Italy<br>Local: the Molise region	Moli-Sani study. Adults aged over 35 (mean age 54.4); percentage male: 47.30%. Randomly recruited.<br>Total n=21,001.	Serial cross-sectional study of participants recruited before and after the recession. Means and p-values of calorie intake adjusted for age and sex.	Exposure: Commence-ment of GR (time)<br>Time point 1: Recruited in 2005-2006 (n=6999)<br>Time point 2: Recruited in 2007-2010 (n=14,002)	Calorie intake (means + SD) using Italian EPIC food frequency questionnaire.	Mean calories 2005-2006: 2228 ± 675.<br>Mean calories 2007-2010: 2101 ± 614<br>p-value <0.0001	Selection total: 3<br>Comparability total: 2<br>Outcomes total: 2<br>**Total score: 7**
Griffith et al, 2013	National: UK<br>Local: n/a	Kantar Worldpanel data.<br>N=15,850.	Longitudinal.<br>Used three time-period dummies and controlled for month and household fixed effects.<br>Percentage change in calories and real expenditure per calorie controlled for month effects and permanent differences in real expenditure per calorie across households.	Exposure: Commence-ment of GR (time)<br>Time point 1: 2005-2007<br>Time point 2: 2008-2009<br>Time point 3:	Percentage change in calories and real expenditure per calorie from 2005–07 to 2008–09 and 2010–12.<br>Percentage change in calorie density.<br>Participants record spending on all grocery purchases brought into the home via an	**1) Percentage change in calories:**<br>Single non-pensioners:<br>2008-09: –0.3, 2010-12: 0.2<br>Single pensioners:<br>2008-09: –3.5, 2010-12: –5.6<br>Couple non-pensioners:<br>2008-0: –1.0, 2010-12: –3.7<br>Couple pensioners:<br>2008-09: –2.2, 2010-12: –4.1<br>Multi-adult households:<br>2008-09: –1.1, 2010-12: –4.1<br>Single parents:<br>2008-09: –5.4, 2010-12: –7.5<br>2+ adults, young children:<br>2008-09: –5.3, 2010-12: –9.8<br>2+ adults, older children:	Selection total: 3<br>Comparability total: 2<br>Outcomes total: 2<br>**Total score: 7**

			The change in calorie density was divided into a 'between' component (the change that was due to people substituting between food types) and a 'within' component (the change that was due to people substituting within food types).		electronic hand-held scanner in the home.	2008-09: –3.6, 2010-12: –8.4<br>All households:<br>2008-09: –1.8, 2010-12: –3.6<br>All differences are statistically different from zero at the 99% level apart from 'single non-pensioners' (2008–09 and 2010–12)<br>**Percentage change in real expenditure per calorie:**<br>Single non-pensioners:<br>2008-09: –3.0, 2010-12: –5.7<br>Single pensioners:<br>2008-09: –1.3, 2010-12: –3.6<br>Couple non-pensioners:<br>2008-09: –2.3, 2010-12: –5.1<br>Couple pensioners:<br>2008-09: –1.0, 2010-12: –4.1<br>Multi-adult households:<br>2008-09: –1.5, 2010-12: –4.2<br>Single parents:<br>2008-09: –2.5 2010-12: –6.6<br>2+ adults, young children:<br>2008-09: –4.3, 2010-12: –9.0<br>2+ adults, older children:<br>2008-09: –2.1, 2010-12: –4.9<br>All households:<br>2008-09: –2.1, 2010-12: –5.2<br>All changes are statistically different from zero at the 99% level.<br>**Percentage change in calorie density:**<br>Single non-pensioners: Actual: 4.4, between: 2.9, within: 1.6<br>Single pensioners: Actual 5.4, between: 4.6, within: 0.1<br>Couple non-pensioners: Actual 3.7, between: 2.5, within: 1.0	

						Couple pensioners: Actual 4.9, between: 4.1, within: 0.7<br>Multi-adult households: Actual 4.3, between: 3.0, within: 1.1<br>Single parents: Actual 5.3, between: 3.3, within: 1.6<br>2+ adults, young children: Actual 6.6, between: 4.6, within: 1.9<br>2+ adults, older children: Actual 3.6, between: 1.5, within: 1.9<br>All changes are statistically different from zero at the 99% level apart from 'Single pensioner – Within'.	
Griffith et al, 2016a	National: UK<br>Local: n/a	Kantar Worldpanel data for UK households. n=14,694.	Longitudinal study. Change in calories. Price paid per calorie is denoted as P=P (*e*, **z**, ϕ)<br>Where *e* denotes shopping effort, **z** is the characteristics of the shopping basket (monthly purchases) and ϕ denotes other factors including common time-varying factors, regional time-varying factors, household level characteristics and time-varying household time-varying character-istics.	Exposure: Commence-ment of GR (time).<br>Time point 1: 2005-2007,<br>Time point 2: 2010-12	Calories purchased (per adult equivalent per day) and average price paid per calorie. Outcome data of food purchases from all types of stores using an electronic hand-held scanner in the home.	**Mean calorie intake:**<br>2005-2007: 2300, 2010-2012: 2274, percent change: - 1.10.<br>Pre-school children:<br>2005-7: 2011, 2010-12: 1931, Percentage change: - 3.99<br>School-age children:<br>2005-7: 2041, 2010-12: 1948, Percentage change: - 4.57<br>Adults:<br>2005-7: 2288, 2010-12: 2295, Percentage change: 0.29<br>Pensioners:<br>2005-7: 2530, 2010-12: 2497, Percentage change: - 1.32<br>Working high-income:<br>2005-7: 2028, 2010-12: 2011, Percentage change: - 0.86<br>Working mid-income:<br>2005-7: 2150, 2010-12: 2099, Percentage change: - 2.37<br>Working low-income:<br>2005-7: 2170, 2010-12: 2131,	Selection total: 3<br>Comparability total: 2<br>Outcomes total: 2<br>**Total score: 7**

						Percentage change: - 1.81<br>Unemployed:<br>2005-7: 2271, 2010-12: 2230,<br>Percentage change: -1.78<br>**Price paid per calorie:**<br>Change in price paid per calorie: 17.74 (log points) (19.4%)<br>Change in price paid per calorie without change in shopping behaviour: 20.34 log points (22.5%)<br>Change in price paid per calorie with changes in within-household behaviour: - 2.59 (3.1% reduction)<br>Contribution made by changes in behaviour:<br>Shopping effort: - 1.06<br>(40.8% reduction)<br>Number of shopping trips: - 0.02<br>(0.8% reduction)<br>Number of chains visited: 0.03<br>(1.2% increase)<br>Savings from discounter: - 0.09<br>(3.5% reduction)<br>Savings from sales: - 0.97<br>(37.6% reduction)<br>Changes in nutrient characteristics:<br>Total: - 0.93 (35.8% reduction)<br>Protein: - 0.43 (16.7% reduction)<br>Saturated fat: - 0.22 (8.5% reduction)<br>Unsaturated fat: 0.05 (- 1.9% reduction)<br>Sugar: 0.01 (- 0.4% reduction)<br>Fibre: - 0.39 (15.1% reduction)<br>Salt: 0.06 (- 2.3% reduction)<br>Fruit: 0.28 (- 10.6% reduction)<br>Vegetables: - 0.23 (8.9% reduction)	

						Dairy: 0.00 (0.00% reduction)<br>Cheese and fats: - 0.00 (0.00% reduction)<br>Poultry and fish: - 0.11 (4.3% reduction)<br>Red meat and nuts: 0.04 (- 1.6% reduction)<br>Drinks: - 0.04 (1.6% reduction)<br>Prepared sweets: 0.11 (- 4.3% reduction)<br>Prepared savoury: 0.02 (- 0.8% reduction)<br>Alcohol: - 0.08 (3.1% reduction)	
Hasan, 2019	National: Bangladesh<br>Local: n/a	Bangladesh Household Income and Expenditure Survey.<br>Repeated cross-sectional study using two-stage stratified random sampling.<br>Analysis was done for those who buy rice (compared to autarkic households and rice sellers, but there was no significant difference between these types).	Serial cross-sectional design.<br>The study used difference-in-difference framework and OLS models including district fixed effects and employing clustered standard errors (weighted).	Exposure: Commence-ment of GR (time).<br>Time point 1: 2005 (n=4,978), time point 2: 2010 (n=6,744).	Total calorie intake per day using food composition data to create caloric intake profiles. Weighted based on adult equivalent calorie requirements.	2010 coefficient: 13.70 (41.47).	Selection total: 3<br>Comparability total: 2<br>Outcomes total: 2<br>**Total score: 7**

		n=11,722					
Iannotti & Robles, 2011	Setting: Guatemala<br>Local: n/a	Encuesta Nacional Sobre Condiciones de Vida (ENCOVI) 2006.<br>n= 13,686	Quadratic Almost Ideal Demand System for food consumption under two different price scenarios.<br>Kernel density estimates used to examine the distributions of calorie intakes (per adult equivalent).	Exposure: price changes using two scenarios: actual changes between 2006 and 2008, with Scenario B representing a 10% increase across all food groups.<br>Time point: 2006	Total calorie intake per day using food composition data to create caloric intake profiles. Weighted based on adult equivalent calorie requirements.	National<br>Total: Before: 2,521, after: 2,542, change: –24<br>Wealth quintile 1 (poor): Before: 1,576, after: 1,481, change: –115<br>Wealth quintile 2: Before: 2,281, after: 2,105, change: -123<br>Wealth quintile 3: Before: 2,716, after: 2,595, change: –74<br>Wealth quintile 4: Before: 2,975, after: 3,071, change: 38<br>Wealth quintile 5 (rich): Before: 3,120, after: 3,631, change: 466<br>Households with children <2 years<br>Total: Before: 2,353, after: 2,307, change: –61<br>Wealth quintile 1 (poor): Before: 1,609, after: 1,517, change: -130<br>Wealth quintile 2: Before: 2,383, after: 2,261, change: -102<br>Wealth quintile 3: Before: 2,812, after: 2,667, change: - 60<br>Wealth quintile 4: Before: 3,154, after: 3,307, change: 206<br>Wealth quintile 5 (rich): Before: 3,047, after: 3,797, change: 706	Selection total: 1<br>Comparability total: 1<br>Outcomes total: 1<br>**Total score: 3**
Iannotti & Robles, 2011	Setting: Honduras<br>Local: n/a	Encuesta de Condiciones de Vida (ENCOVI) 2004 n= 8175	Quadratic Almost Ideal Demand System for food consumption under two different price scenarios.<br>Kernel density estimates used to examine the	Exposure: price changes using two scenarios: actual changes between 2006 and 2008, with Scenario	Total calorie intake per day using food composition data to create caloric intake profiles. Weighted based on adult	National<br>Total: Before: 2,370, after: 2,336, change: –32<br>Wealth quintile 1 (poor): Before: 1,986, after: 1,591, change: –330<br>Wealth quintile 2: Before: 2,312, after: 2,135, change: –133<br>Wealth quintile 3: Before: 2,409, after: 2,349, change: –15	Selection total: 1<br>Comparability total: 1<br>Outcomes total: 1<br>**Total score: 3**

			distributions of calorie intakes (per adult equivalent).	B representing a 10% increase across all food groups.<br>Time point: 2006	equivalent calorie requirements.	Wealth quintile 4: Before: 2,632, after: 2,713, change: 55<br>Wealth quintile 5 (rich): Before: 2,705, after: 3,348, change: 651<br><u>Households with children <2 years</u><br>Total: Before: 2,418, after: 2,325, change: –110<br>Wealth quintile 1 (poor): Before: 1,997, after: 1,658, change: –357<br>Wealth quintile 2: Before: 2,436, after: 2,274, change: –117<br>Wealth quintile 3: Before: 2,544, after: 2,499, change: –59<br>Wealth quintile 4: Before: 2,863, after: 2,994, change: 55<br>Wealth quintile 5 (rich): Before: 2,955, after: 3,756, change: 767	
Iannotti & Robles, 2011	Setting: Nicaragua<br>Local: n/a	Encuesta Nacional de Hogares Sobre Medición de Nivel de Vida (EMNV) n= 4959	Quadratic Almost Ideal Demand System for food consumption under two different price scenarios.<br>Kernel density estimates used to examine the distributions of calorie intakes (per adult equivalent).	Exposure: price changes using two scenarios: actual changes between 2006 and 2008, with Scenario B representing a 10% increase across all food groups.<br>Time point: 2006	Total calorie intake per day using food composition data to create caloric intake profiles. Weighted based on adult equivalent calorie requirements.	<u>National</u><br>Total: Before: 2,642, after: 2,496 change: –226<br>Wealth quintile 1 (poor): Before: 1,655, after: 1,242, change: –414<br>Wealth quintile 2: Before: 2,363, after: 1,968, change: –380<br>Wealth quintile 3: Before: 2,803, after: 2,548, change: –280<br>Wealth quintile 4: Before: 3,134, after: 3,036, change: –82<br>Wealth quintile 5 (rich): Before: 3,578, after: 4,328, change: 553<br><u>Households with children <2 years</u><br>Total: Before: 2,509, after: 2,287, change: –260<br>Wealth quintile 1 (poor): Before: 1,669, after: 1,240, change: –413	Selection total: 1<br>Comparability total: 1<br>Outcomes total: 1<br>**Total score: 3**

						Wealth quintile 2: Before: 2,497, after: 2,145, change: –309<br>Wealth quintile 3: Before: 3,253, after: 3,109, change: –169<br>Wealth quintile 4: Before: 3,144, after: 3,166, change: 176<br>Wealth quintile 5 (rich): Before: 4,144, after: 4,214, change: 316	
Iannotti & Robles, 2011	Setting: Panama<br>Local: n/a	Encuesta de Niveles de Vida (ENV) 2003 n= 6158	Quadratic Almost Ideal Demand System for food consumption under two different price scenarios.<br>Kernel density estimates used to examine the distributions of calorie intakes (per adult equivalent).	Exposure: price changes using two scenarios: actual changes between 2006 and 2008, with Scenario B representing a 10% increase across all food groups.<br>Time point: 2006	Total calorie intake per day using food composition data to create caloric intake profiles. Weighted based on adult equivalent calorie requirements.	National<br>Total: Before: 1,904, after: 1,600, change: –261<br>Wealth quintile 1 (poor): Before: 1,276, after: 762, change: –479<br>Wealth quintile 2: Before: 1,809, after: 1,457, change: –314<br>Wealth quintile 3: Before: 2,059, after: 1,690, change: –235<br>Wealth quintile 4: Before: 2,141, after: 1,986, change: –136<br>Wealth quintile 5 (rich): Before: 2,318, after: 2,311, change: –57<br>Households with children <2 years<br>Total: Before: 1,701, after: 1,330, change: –362<br>Wealth quintile 1 (poor): Before: 1,264, after: 621, change: –552<br>Wealth quintile 2: Before: 1,949, after: 1,498, change: –334<br>Wealth quintile 3: Before: 2,233, after: 1,783, change: –282<br>Wealth quintile 4: Before: 2,151, after: 2,078, change: –130<br>Wealth quintile 5 (rich): Before: 2,030, after: 2,671, change: 424	Selection total: 1<br>Comparability total: 1<br>Outcomes total: 1<br>**Total score: 3**

Iannotti & Robles, 2011	Setting: Haiti<br>Local: n/a	Enquête Budget et Consommation des Ménages 1999-2000 n= 4625	Quadratic Almost Ideal Demand System for food consumption under two different price scenarios.<br>Kernel density estimates used to examine the distributions of calorie intakes (per adult equivalent).	Exposure: price changes using two scenarios: actual changes between 2006 and 2008, with Scenario B representing a 10% increase across all food groups.<br>Time point: 2006	Total calorie intake per day using food composition data to create caloric intake profiles. Weighted based on adult equivalent calorie requirements.	National<br>Total: Before: 1,863, after: 1,661, change: –172<br>Wealth quintile 1 (poor): Before: 1,079, after: 805 change: –249<br>Wealth quintile 2: Before: 1,645, after: 1,357, change: –326<br>Wealth quintile 3: Before: 2,021, after: 1,820, change: –214<br>Wealth quintile 4: Before: 2,689, after: 2,618, change: –53<br>Wealth quintile 5 (rich): Before: 2,633, after: 3,419, change: 290<br>Households with children <2 years<br>Total: Before: 1,635, after: 1,495, change: –165<br>Wealth quintile 1 (poor): Before: 1,095, after: 831, change: –244<br>Wealth quintile 2: Before: 1,659, after: 1,454, change: –237<br>Wealth quintile 3: Before: 1,904, after: 2,159, change: –11<br>Wealth quintile 4: Before: 3,229, after: 3,395, change: –2<br>Wealth quintile 5 (rich): Before: 2,498, after: 3,834, change: 851	Selection total: 1<br>Comparability total: 1<br>Outcomes total: 1<br>**Total score: 3**
Iannotti & Robles, 2011	Setting: Ecuador<br>Local: n/a	Encuesta de Condiciones de Vida (ECV) 2006 n= 13,018	Quadratic Almost Ideal Demand System for food consumption under two different price scenarios.<br>Kernel density estimates used to examine the distributions of	Exposure: price changes using two scenarios: actual changes between 2006 and 2008, with Scenario B representing	Total calorie intake per day using food composition data to create caloric intake profiles. Weighted based on adult equivalent calorie requirements.	National<br>Total: Before: 2,105, after: 1,747, change: –318<br>Wealth quintile 1 (poor): Before: 1,296, after: 966, change: –305<br>Wealth quintile 2: Before: 1,992, after: 1,527, change: –437<br>Wealth quintile 3: Before: 2,301, after: 1,870, change: –361	Selection total: 1<br>Comparability total: 1<br>Outcomes total: 1<br>**Total score: 3**

			calorie intakes (per adult equivalent).	a 10% increase across all food groups.<br>Time point: 2006		Wealth quintile 4: Before: 2,583, after: 2,256, change: –271<br>Wealth quintile 5 (rich): Before: 2,685, after: 2,571, change: –107<br><u>Households with children <2 years</u><br>Total: Before: 1,968, after: 1,641, change: –328<br>Wealth quintile 1 (poor): Before: 1,251, after: 912, change: –258<br>Wealth quintile 2: Before: 2,067, after: 1,595, change: –521<br>Wealth quintile 3: Before: 2,424, after: 1,954, change: –420<br>Wealth quintile 4: Before: 2,605, after: 2,434, change: –189<br>Wealth quintile 5 (rich): Before: 2,648, after:2,666, change: 33	
Iannotti & Robles, 2011	Setting: Peru<br>Local: n/a	Encuesta Nacional de Hogares (ENAHO) 2006<br>n= 20,577	Quadratic Almost Ideal Demand System for food consumption under two different price scenarios.<br>Kernel density estimates used to examine the distributions of calorie intakes (per adult equivalent).	Exposure: price changes using two scenarios: actual changes between 2006 and 2008, with Scenario B representing a 10% increase across all food groups.<br>Time point: 2006	Total calorie intake per day using food composition data to create caloric intake profiles. Weighted based on adult equivalent calorie requirements.	<u>National</u><br>Total: Before: 2,586, after: 2,392, change: –175<br>Wealth quintile 1 (poor): Before: 1,835, after: 1,539, change: –272<br>Wealth quintile 2: Before: 2,412, after: 2,062, change: –326<br>Wealth quintile 3: Before: 2,749, after: 2,489, change: –57<br>Wealth quintile 4: Before: 3,031, after: 2,963, change: –71<br>Wealth quintile 5 (rich): Before: 3,257, after: 3,649, change: 413<br><u>Households with children <2 years</u><br>Total: Before: 2,475, after: 2,298, change: –167<br>Wealth quintile 1 (poor): Before: 1,901, after:1,695, change: –229	Selection total: 1<br>Comparability total: 1<br>Outcomes total: 1<br>**Total score: 3**

						Wealth quintile 2: Before: 2,597, after: 2,332, change: –297<br>Wealth quintile 3: Before: 2,872, after:2,805, change: –142<br>Wealth quintile 4: Before: 3,197, after: 3,465, change: 160<br>Wealth quintile 5 (rich): Before: 3,399, after: 3,659, change: 400	
Marcotte-Chenard	National: USA<br>Local: n/a	National Health and Nutrition Examination Survey. Adults aged between 20-85 (average age 49), 48.1% male.<br>N=38,541	Serial cross-sectional study.<br>Factorial ANOVAs (post hoc test and contrast) used to compare 1999-2006 intervals to 2006-2007 intervals.	Exposure: Commence-ment of GR (time).<br>Time point 1: 1999-2006<br>Time point 2: 2007-2008.	24-hr dietary recall used to calculate average total daily calorie intake in men and women.	Men:<br>1999-2006: 2318 ± 1013, 2007-2008: 2233 ± 875; P = 0.0001<br>Women:<br>1999-2006: 1786 ± 762, 2007-2008: 1688 ± 625 calories/day; P = 0.0001	Selection total: 3<br>Comparability total: 1<br>Outcomes total: 2<br>**Total score: 6**
Mohseni-Cheraglou, 2016	Global: 63 countries (and 100 recessions).	Reinhart and Rogoff's dataset on financial crises; FAOSTAT data 2010. 93 observations.	Investigating whether growth rates of different variables are affected by financial crises and computing an average of these changes over all crisis episodes in all countries.	Exposure: value of a currency falling by 15% or more against the US dollar or banking distress including closures, mergers and government takeovers.<br>Data from 1981-2007.	Calorie Intake per Capita Per Day	Average change in growth rate (t-test): -2.1 (p<0.01)<br>Crises with recessions, average change in growth rate (t-test): -2.8 (p<0.01)<br>Crises without recessions, average change in growth rate (t-test): -1.4 (p<0.05).	Selection total: 2<br>Comparability total: 0<br>Outcomes total: 1<br>**Total score: 3**
Ng	National: USA<br>Local: n/a	National Health and Nutrition	Cross-sectional. Comparison of calorie intakes over	Exposure: Commence-	Total calorie intake per day using food	**Children aged 2-18:**<br>Mean calories consumed per day<br>2003-4: 2118 (SE 23)	Selection total: 4

		Examination Survey.<br>Children and adults. Multi-stage, stratified-area probability sample of US population.<br>N for children ranges from 2966 to 3778, adults n ranges from 2449 to 3038.	time in adults and children. Statistical difference assessed using 2-sample t tests.	ment of GR (time)<br>Time point 1: 2003-2004<br>Time point 2: 2005-2006<br>Time point 3: 2007-2008<br>Time point 4: 2009-2010	composition data to create caloric intake profiles.	Mean calories consumed per day 2005-6: 2027 (SE 33)<br>Mean calories consumed per day 2007-8: 1907 (SE 25) – significantly different from 2003-2004, p<0.01.<br>Mean calories consumed per day 2009-10: 1908 (SE 25) – significantly different from 2003-2004, p<0.01.<br>Mean annualised change: -35<br>Largest annualised decreases from Mexican American children (-47 kcal/day), children from low-income families (-45 kcal/day) and children whose household head had a high school education (-51 kcal/day).<br>No significant declines observed from 2003-2004 to 2009-2010 in adolescents, non-Hispanic blacks and children whose household head had less than high school education.<br>**Adults aged 19+:**<br>Mean calories consumed per day 2003-4: 2220 (SE 16)<br>Mean calories consumed per day 2005-6: 2201 (SE 33)<br>Mean calories consumed per day 2007-8: 2121 (SE 29)<br>Mean calories consumed per day 2009-10: 2135 (SE 19) – significantly different from 2003-2004, p<0.01.<br>Mean annualised change: -14<br>Few significant declines in energy intake for adults – significant declines only seen in Mexican Americans, women and individuals with some college education.	Comparability total: 2<br>Outcomes total: 2<br>**Total score: 8**

Shabnam et al., 2016	National: Pakistan<br>Local: n/a	Household Integrated Economic Survey (HIES), a nationally representative survey of rural and urban areas (14 big cities and 81 districts in each of the country's four provinces).<br>Mean age: 45, Female-headed households 7.5% in 2005 and 8.4% in 2010.<br>N= 14,863 and 15,191	Serial cross-sectional. Demand equation for price elasticity of calories.	Exposure: Commence-ment of GR (time)<br>Time point 1: 2005-6 (n=14,863)<br>Time point 2: 2010-11<br>n= 15,191	The data on household food consumption covered a period of 14 days and 30-days call period.<br>Price elasticity of calories.	Price elasticity of calories<br>2005-2006: -0.03<br>2010-2011: -0.21	Selection total: 3<br>Comparability total: 2<br>Outcomes total: 2<br>**Total score: 7**
Smed et al., 2017	National: Denmark<br>Local: n/a	GfK Panel Services Scandinavia of households of working age.<br>N=3440	Longitudinal.<br>Fixed methods econometric methods to control for unobserved heterogeneity.<br>Unemployment, single, location and number of children included in model.<br>If increasing CCI is associated with increasing consumption, an economic downturn	Exposure: Consumer Confidence Interval as a proxy for economic downturn.<br>Time: January 2008 to December 2012.	Energy in kJ per person per month.<br>Constructed consumption per individual in households by dividing each household's consumption data with weights constructed from gender- and age-dependent daily energy intake.	CCI (β1): coefficient: 143·43, P value: 0·637	Selection total: 4<br>Comparability total: 2<br>Outcomes total: 2<br>**Total score: 8**

			is associated with decreasing consumption and vice versa.				
Todd, 2014	National: USA<br>Local: n/a	National Health and Nutrition Examination Survey.<br>N=9,839, adults born between 1946-1985 so between the ages of 20 – 64 during the study period. 48-49% male.	Serial cross-sectional. Multivariate linear regression models were used to estimate the conditional changes in outcome variables. Used weighted ordinary least squares with SE accounting for the complex sample design. Model (conditioning) includes age, household size, and indicators for gender, ethnicity, marital intake, data collected during weekend, and for the older cohort, education as controls.	Exposure: Commence-ment of GR (time)<br>Time point 1: 2005-2006 N=3,014<br>Time point 2: 2007-2008 N=3,294<br>Time point 3: 2009-2010 N=3,531	Daily calories from one-day dietary recall.	**Total daily calories**<br><u>Unconditional and conditional differences in mean outcomes between 2005-06 and 2009- 10:</u><br>Unconditional: - 117.73<br>Conditional upon age: -90.37 (difference from unconditional is statistically significant with p<0.01)<br>Conditional upon age, other demographics: - 78.45<br>Conditional upon age, other demographics, and income: -78.79<br>Difference in variable mean between full model (conditional upon age, other demographics and income) and unconditional is statistically significant with p<0.05<br><u>Conditional changes by subgroups:</u><br>Born 1946-85, some college or more: 2005-06: 2325.61, change 2005-06 to 2009-10: -85.89 (change is statistically significant at P<0.05)<br>Born 1946-85, no college education: 2005-06: 2333.15, change 2005-06 to 2009-10: -70.15<br>Men, born 1946-85, no college: 2005-06: 2794.45, change 2005-06 to 2009-10: -103.93<br>Adults born before 1946: 2005-06: 1788.48, Change 2005-06 to 2009-10: -2.08	Selection total: 3<br>Comparability total: 2<br>Outcomes total: 1<br>**Total score: 6**

Todd, 2017	National: USA<br>Local: n/a	National Health and Nutrition Examination Survey. Includes working age adults between 25-65 (n=12,129), and a secondary sample of 15-24 year olds (23-32 year olds in 2013-2014) (n=5197).	Serial cross-sectional. Used multivariate linear regression models and ordinary least squares. Adjusted for age, household income relative to poverty, household size, and indicators for gender, ethnicity, marital intake, data collected during weekend, and for the older cohort, education as controls.	Exposure: Commence-ment of GR (time)<br>Time point 1: 2005-2006<br>Time point 2: 2007-2008<br>Time point 3: 2009-2010<br>Time point 4: 2013-2014	Daily calories from one-day recall using Automated Multiple Pass Method (log total energy)	<u>Conditional differences with their standard errors, and percentage change in estimated difference from unconditional difference in log total energy intake:</u><br>2007-08: β −0·03, SE: 0·02<br>2009-10: β −0·03, SE: 0·02 (estimate is statistically significant P<0·05), percentage change: - 40<br>2011-12: β 0.00, SE: 0·02<br>2013-14 β −0·03, SE: 0·02, percentage change: - 57<br>Constant: β 7.59, SE: 0.00 (estimate is statistically significant P<0·05)	Selection total: 4<br>Comparability total: 2<br>Outcomes total: 2<br>**Total score: 8**
Dietary Quality							
Bonaccio et al., 2014	National: Italy<br>Local: the Molise region	Moli-Sani study. Adults aged over 35 (mean age 54.4); percentage male: 47.30%. Randomly recruited.<br>Total n=21,001.	Serial cross-sectional study of participants recruited before and after the recession. Multivariable binomial (Poisson) regression with log link function. Covariates included total energy intake, total physical activity, BMI, smoking, hypertension, hypercholesterol-aemia and diabetes. Used an interaction term to test for a difference between two time periods.	Exposure: Commence-ment of GR (time).<br>Time point 1: Recruited in 2005-2006 (n=6999)<br>Time point 2: Recruited in 2007-2010 (n=14,002)	Mediterranean Diet Adherence using Italian EPIC food frequency questionnaire and Italian Mediterranean Index (IMI) (higher number indicates higher adherence to Mediterranean diet, with a score of more than 5 indicating high adherence).<br>Also Total Food Dietary	High adherence (IMI score ≥ 5):<br>2005-2006: 31.1%, 2007-2010: 18.3% (difference -13.0, p-value for difference between time periods <0.001).<br>Prevalence ratios (95% CI) of high adherence to Mediterranean diet according to SE indicators over time.<br>**Age groups:**<br>2005-2006: 35-43: ref; 44-53: 1.21 (1.09-1.35); 54-59: 1.35 (1.19–1.53); 60-70: 1.50 (1.32–1.70); 70+: 1.35 (1.14–1.59)<br>2007-2010: 35-43: ref;<br>44-53: 1.01 (0.91–1.12);<br>54-59: 1.30 (1.15–1.47);<br>60-70: 1.27 (1.12–1.45);<br>70+: 1.12 (0.94–1.34)<br>**Sex:**	Selection total: 3<br>Comparability total: 2<br>Outcomes total: 2<br>**Total score: 7**

					Antioxidant Content score, using Italian EPIC food frequency questionnaire.	2005-2006: Women: ref, men 0.77 (0.71–0.83)<br>2007-2010: Women: ref, men 0.75 (0.69–0.82)<br>**Wealth Score:**<br>2005-2006: Low: ref,<br>Medium: 1.04 (0.94–1.16),<br>High: 1.05 (0.94–1.16),<br>Non-respondent: 1.03 (0.92–1.15)<br>2007-2010: Low: ref,<br>Medium: 1.10 (0.98–1.22),<br>High: 1.31 (1.18–1.46),<br>non-respondent: 1.00 (0.91–1.11)<br>**Education:**<br>2005-6: ≤8 years of study: ref,<br>9-13 years of study: 1.02 (0.93–1.11),<br>>13 years of study: 1.16 (1.04–1.31)<br>2007-10: ≤8 years of study: ref,<br>9-13 years of study: 1.24 (1.14–1.35),<br>>13 years of study: 1.32 (1.17–1.50)<br>**Total Food Dietary Antioxidant Content score:**<br>2005/6: 5.9 (50.2)<br>2007-2010: −3.4 (47.6)<br>p-value <0.0001	
Brinkman et al., 2010	National: Haiti<br>Local: 5 villages (Acul Samedi, Ferrier, Beau Roc, Grison Garde, and Dupity)	World Food Programme conducted household-level food security assessments.<br>N=517	Regression model and Ordinary Least Squares estimates linking changes in Food Consumption Score with changes in food prices.	Exposure: changes in food prices (price variable: rice).<br>Time point 1: January, June and September 2006	Food Consumption Score, calculated using eight different food groups (main staples, pulses, vegetables, fruit, meat and fish, milk, sugar, oil) over seven days	Intercept: 2.90 (significant at 1%)<br>Prices: -0.23 (significant at 5%)	Selection total: 2<br>Comparability total: 0<br>Outcomes total: 0<br>**Total score: 2**

				Time point 2: January, June and September 2007	and multiplying frequency by food weight and summing all groups to create a consumption score, with a score of over 35 being considered as an acceptable diet		
Brinkman et al., 2010	National: Nepal<br>Local: 3 zones (mountains, Terai, and Hill districts)	World Food Programme conducted household-level food security assessments.<br>N=600	Regression model and Ordinary Least Squares estimates linking changes in Food Consumption Score with changes in food prices.	Exposure: changes in food prices (price variable: weighted commodity index).<br>Time point 1: July - September 2008<br>Time point 2: October – December 2008	Food Consumption Score (see above)	Intercept: 4.09 (significant at 1%)<br>Prices: -0.05 (significant at 10%)	Selection total: 2<br>Comparability total: 0<br>Outcomes total: 0<br>**Total score: 2**
Brinkman et al., 2010	National: Niger<br>Local: 357 villages	World Food Programme conducted household-level food security assessments.<br>N=4376	Regression model and Ordinary Least Squares estimates linking changes in Food Consumption Score with changes in food prices.	Exposure: changes in food prices (price variable: millet).<br>June, August, November,	Food Consumption Score (see above)	Intercept: 4.13 (significant at 1%)<br>Prices: -0.08 (significant at 1%)	Selection total: 2<br>Comparability total: 0<br>Outcomes total: 0<br>**Total score: 2**

				December 2007.			
Dave et al., 2012	National: USA<br>Local: n/a	Behavioural Risk Factor Surveillance System. Adults aged between 26-58.<br>N=56,354 (answered all healthy/ unhealthy food questions)	Serial cross-sectional. Reduced-form cross-equation (fixed effects) estimates of the average effect of state unemployment on healthy and unhealthy food consumption seemingly unrelated regression.<br>Controlled for gender, education, age, marital status, ethnicity and state indicators.	Exposure: area-level unemploy-ment rates.<br>Time: 1990 – 2009 (excluding 2004, 2006 and 2008).	How often do you eat (FOOD) with options of times per day, week, month, or year?<br>Healthy foods: carrots, fruit, fruit juice, green salad, and vegetables.<br>Unhealthy food: snacks, hamburgers, hot dogs, French fries, fried chicken, and doughnuts	**Healthy food consumption:**<br>All: −0.0057 (0.0014) [0.000]<br>Ages 26–58: −0.0048 (0.0013) [0.000]<br>Ages 44–58: −0.0030 (0.0011) [0.009]<br>Ages 65+: −0.0004 (0.0015) [0.767]<br>Males: −0.0062 (0.0012) [0.000]<br>Females: −0.0035 (0.0015) [0.015]<br>Married: −0.0058 (0.0018) [0.001]<br>Unmarried: −0.0029 (0.0008) [0.000]<br>Less than college: −0.0055 (0.0011) [0.000]<br>College plus: −0.0033 (0.0025) [0.187]<br>**Unhealthy food consumption:**<br>All: 0.0074 (0.0065) [0.261]<br>Ages 26–58: 0.0106 (0.0076) [0.161]<br>Ages 44–58: 0.0137 (0.0104) [0.185]<br>Ages 65+: −0.0066 (0.0058) [0.253]<br>Males: −0.0022 (0.0073) [0.766]<br>Females: 0.0160 (0.0079) [0.043]<br>Married: 0.0121 (0.0073) [0.099]<br>Unmarried: −0.0022 (0.0103) [0.830]<br>Less than college: 0.0099 (0.0080) [0.216]<br>College plus: −0.0011 (0.0080) [0.895]	Selection total: 3<br>Comparability total: 2<br>Outcomes total: 2<br>**Total score: 7**
Foscolou, 2017	20 Mediterrane an islands: the Republic of Malta (n=250), Sardinia (n=60) and Sicily (n=50)	MEDIS Study. People above 65, 50% male.<br>N=2749	Serial Cross-sectional study of participants recruited before and after the recession. Scores compared before and after 2009 using independent samples t-test.	Exposure: Commence-ment of GR (time).<br>Time point 1: Recruited between 2005-2008 (n=1220).	Adherence to Mediterranean Diet using MedDietScore (ranging from 0-55; 0-29 low adherence, 30-37 medium adherence, 38-55	**Enrolled before 2009:**<br>MedDietScore: 34 ± 4<br>Low adherence: n=174 (14%),<br>Medium adherence: n=868 (72%)<br>High adherence: n=173 (14%)<br>**Enrolled after 2009:**<br>MedDietScore: 32 ± 5<br>Low adherence: n=524 (36%)<br>Medium adherence: n=721 (50%)	Selection total: 2<br>Comparability total: 0<br>Outcomes total: 1<br>**Total score: 3**

	in Italy, the Republic of Cyprus (n=300), Gökçeada (n=55) in Turkey and the Greek islands of Lesbos (n=142), Samothraki (n=100), Cephalonia (n=115), Crete (n=131), Corfu (n=149), Limnos (n=150), Ikaria (n=76), Syros (n=151), Naxos (n=145), Zakynthos (n=103), Salamina (n=147), Kassos (n=52), Rhodes and Karpathos (n=149), Tinos			Time point 2: recruited between 2009-2015 (n=1529)	high adherence). Dietary habits assessed through a food frequency questionnaire.	High Adherence: n=213 (14%).<br>**Difference in MedDietScore:**<br>P-value <0.001 (independent samples t-test)<br>**Difference in low adherence:**<br>P-value <0.001 (Pearson chi-squared test)	

	(n=129), as well as the rural region of east Mani (n=295).						
Griffith et al, 2016a	National: UK<br>Local: n/a	Kantar Worldpanel data for UK households. n=14,694.	Longitudinal study. HEI scores and percentage change.	Exposure: Commence-ment of GR (time).<br>Time point 1: 2005-2007,<br>Time point 2: 2010-12	Healthy Eating (HEI) index. The HEI gives a score between 0 and 100 based on amount per 1000 calories of different food groups and nutrients (fruit, vegetables, grains, milk, meat, oils, sodium, and saturated fat). Outcome data of food purchases from all types of stores using an electronic hand-held scanner in the home.	**All Households:**<br>Max score: 100<br>Mean in 2005-7: 49.0<br>Change to 2010-12: 0.72<br>% change to 2010-12: 1.5<br>'Good' change: change to 2010-12: 1.45, % change to 2010-12: 3.0<br>'Bad' change: change to 2010-12: - 0.72, % change to 2010-12: 1.5<br>**Pre-school children:**<br>Mean in 2005-7: 48.7<br>Change to 2010-12: 1.52<br>% change to 2010-12: 3.1%<br>'Good' change: 3.02,<br>'Bad' change: - 1.51<br>**School-age children:**<br>Mean in 2005-7: 46.1<br>Change to 2010-12: 1.03<br>% change to 2010-12: 2.2<br>'Good' change: 1.90,<br>'Bad' change: - 0.87<br>**Adults:**<br>Mean in 2005-7: 47.8<br>Change to 2010-12: 1.46<br>% change to 2010-12: 3.1<br>'Good' change: 1.93,<br>'Bad' change: - 0.46<br>**Pensioners:**<br>Mean in 2005-7: 51.5<br>Change to 2010-12: - 0.23<br>% change to 2010-12: - 0.4	Selection total: 3<br>Comparability total: 2<br>Outcomes total: 2<br>**Total score: 7**

						'Good' change: 0.91,<br>'Bad' change: - 1.14<br>**Working high-income**:<br>Mean in 2005-7: 49.6<br>Change to 2010-12: 0.87<br>% change to 2010-12: 1.8<br>'Good' change: 1.78,<br>'Bad' change: - 0.91<br>**Working mid-income:**<br>Mean in 2005-7: 48.0<br>Change to 2010-12: 1.03<br>% change to 2010-12: 2.1<br>'Good' change: 1.78,<br>'Bad' change: - 0.75<br>**Working low-income:**<br>Mean in 2005-7: 46.6<br>Change to 2010-12: 2.01<br>% change to 2010-12: 4.3<br>'Good' change: 2.44,<br>'Bad' change: - 0.43<br>**Unemployed:**<br>Mean in 2005-7: 46.7<br>Change to 2010-12: 1.11<br>% change to 2010-12: 2.4<br>'Good' change: 1.67,<br>'Bad' change: - 0.56	
Griffith et al, 2013	National: UK<br>Local: n/a	Kantar Worldpanel data.<br>N=15,850.	Longitudinal. Regressed variables on three time-period dummies and controlled for month and household fixed, estimating regression separately by household.	Exposure:<br>Time point 1: 2005-2007<br>Time point 2: 2008-2009<br>Time point 3: 2010-2012	1) Nutrient Profile Model.<br>Based on a food item’s energy density, saturated fat, sodium, sugar content (all of which contribute negatively), protein, fibre, and	**1) NPM**<br>Single non-pensioners:<br>2008-09: – 0.16, 2010-12: – 0.20,<br>Single pensioners:<br>2008-09: – 0.26, 2010-12: – 0.31,<br>Couple non-pensioners:<br>2008-09: – 0.12, 2010-12: – 0.13,<br>Couple pensioners:<br>2008-09: – 0.15, 2010-12: – 0.24,<br>Multi-adult households:	Selection total: 3<br>Comparability total: 2<br>Outcomes total: 2<br>**Total score: 7**

					fruit and vegetable content (which contribute positively). They constructed a weighted average for each household in each month across all the products purchased.<br>2) Healthy Eating Index - constructed based on the amounts per 1000 calories of produce of 12 components, including both food types (fruit, vegetables, grains, milk, meat and oils) and nutrients (saturated fat, sodium, added sugar, solid fat and alcohol). Participants record spending on all grocery purchases via an electronic hand-held scanner in the home	2008-09: – 0.05, 2010-12: – 0.07,<br>Single parents:<br>2008-09: – 0.12, 2010-12: – 0.24<br>2+ adults, young children:<br>2008-09: – 0.14, 2010-12: – 0.26<br>2+ adults, older children:<br>2008-09: – 0.01, 2010-12: – 0.05<br>All households:<br>2008-09: – 0.13, 2010-12: –0.18<br>All the changes are statistically different from zero at the 99% level, apart from '2+ adults, older children (2008–09)'. The NPM score ranges from –6.1 to 21.6, with a mean of 1.45 across all households and months.<br>**2) HEI**<br>Single non-pensioners:<br>2008-09: – 1.25, 2010-12: – 1.57,<br>Single pensioners:<br>2008-09: – 1.97, 2010-12: – 2.74,<br>Couple non-pensioners:<br>2008-09: – 1.02, 2010-12: – 1.13,<br>Couple pensioners:<br>2008-09: – 1.39, 2010-12: – 2.40,<br>Multi-adult households:<br>2008-09: – 0.40, 2010-12: – 0.88<br>Single parents:<br>2008-09: – 1.21, 2010-12: – 2.48<br>2+ adults, young children:<br>2008-09: – 0.56, 2010-12: – 1.28<br>2+ adults, older children:<br>2008-09: – 0.40, 2010-12: – 1.19<br>All households:<br>2008-09: – 1.00, 2010-12: – 1.64.<br>All changes were statistically significant from zero at the 99% level.	

					(purchases brought into the home).	The HEI ranges from 2.9 to 100, with a mean of 50.5 across all households and months.	
Hasan, 2019	National: Bangladesh<br>Local: n/a	Bangladesh Household Income and Expenditure Survey.<br>Repeated cross-sectional study using two-stage stratified random sampling.<br>Analysis was done for those who buy rice (compared to autarkic households and rice sellers, but there was no significant difference between these types).<br>n=11,722	Serial cross-sectional design.<br>The study used difference-in-difference framework and OLS models including district fixed effects and employing clustered standard errors (weighted).	Exposure: Commence-ment of GR (time)<br>Time point 1: 2005 (n=4,978), time point 2: 2010 (n=6,744).	1) Household Dietary Diversity Score which involves counting the number of nutritional food groups consumed by the household in a day to come up with a score that is a number between 0 and 12, determined whether you consumed foods in 12 pre-defined groups (cereals, roots and tubers, pulses and nuts, vegetables, fruit, meat, eggs, fish and seafood, milk and dairy products, oil and fats, sugar and miscellaneous (for example, condiments)).<br>2) Food Consumption Score - calculated using eight different food	1) Household Dietary Diversity Score:<br>Year 2010 coefficient: 0.19 (0.08) $P<0.05$<br>2) Food Consumption Score:<br>Year 2010 coefficient: −0.22 (0.88)<br>3) Number of food groups consumed:<br>Year 2010 coefficient: 0.60 (0.17) $P<0.01$	Selection total: 3<br>Comparability total: 2<br>Outcomes total: 2<br>**Total score: 7**

					groups (main staples, pulses, vegetables, fruit, meat and fish, milk, sugar, oil) over seven days and multiplying frequency by food weights (based on energy, protein and micro-nutrient density of food). Summed to create a consumption score, with a score of over 35 being considered as an acceptable diet.<br>3) Number of food groups consumed		
Kuhns, 2014	National: USA<br>Local: n/a	Neilson Homescan Data. N=4.2 million observations	Panel survey. Multivariate panel regression analysis with healthfulness of monthly shopping baskets modelled as a function of macroeconomic conditions. Controlled for unobserved time-invariant geographic fixed effects.	Exposure: Commence-ment of GR (time)<br>Time: 2004-2010 (dummy variable used for 2007-2009).	The USDA scores are squared-error loss functions, designed to assign penalties for household expenditure shares that deviate from USDA recomm-endations.<br>1) USDAScore1 is the simplest and	**1) USDA Score 1**<br>Overall: mean: 5.949, St. Dev.: 2.357, min: 0.898, max.: 16.364<br>Recession: mean: 5.996, St. Dev.: 2.395, min: 0.898, max.: 16.365<br>Not Recession: mean: 5.932, St. Dev.: 2.343, min: 0.898, max.: 16.364<br><u>Regression results:</u><br>Recession: monthly average: 0.120 (0.222); household FE: 0.451 (0.005) (Significant at the 0.01 level);<br>**2) USDA Score 2**	Selection total: 3<br>Comparability total: 2<br>Outcomes total: 2<br>**Total score: 7**

					operates on the assumption that the Homescan households report 100% food at home purchases to Nielsen. Input an expenditure share of zero for those food groups for which households report no purchases.<br>2) USDAScore2 assumes that households simply have not recorded purchases for completely empty food categories. Therefore, USDAScore2 is calculated based only on those food categories with recorded purchases and expenditure shares greater than zero.<br>3) USDAScore3 assigns penalties only when	Overall: mean: 7.996, St. Dev.: 3.440, min: 0.971, max.: 22.808<br>Recession: mean: 8.059, St. Dev.: 3.483, min: 0.971, max.: 17.874<br>Not Recession: mean: 7.972, St. Dev.: 3.424, min: 0.990, max.: 22.808<br><u>Regression results:</u><br>Recession: monthly average: 0.074 (0.139); household FE: 0.286 (0.009) (Significant at the 0.01 level);<br>**3) USDA Score 3**<br>Overall: mean: 6.430, St. Dev.2.685, min: 0.915, max.: 17.875<br>Recession: mean: 6.487, St. Dev.: 2.728, min: 0.915, max.: 17.874<br>Not Recession: mean: 6.410, St. Dev.: 2.669, min: 0.915, max.: 17.875<br>Regression results:<br>Recession: monthly average: 0.122 (0.205); household FE: 0.425 (0.006) (Significant at the 0.01 level);<br>Dietary quality 4-8% better during recession.	

					households exceed recommend-ations for limited categories or falls short of recommend-ations for categories.		
Martin-Prevel et al, 2012	National: Burkina Faso<br>Local: Ouaga-dougou	Households randomly selected from Ouagadougou census. Average age of household head: 42, household head 86.8% (2007) and 87.8% (2008) male.	Serial cross-sectional. General linear mixed model.<br>Model 0: no adjustment,<br>Model 1: comparison adjusted for age and gender of the head of household, residency in the compound, size of household, youth ratio, dependency ratio, economic score, salary + interaction terms: year × economic score<br>Model 2: same as model 1 + adjustment for household food expenditure/adult equivalent.<br>All analyses took into account the sampling design by including a random EA effect.	Exposure: Commence-ment of GR (time)<br>Time Point 1: July 2007 (n=3017)<br>Time Point 2: July 2008 (n=3002)	Index member Dietary Diversity Score (preceding 24 hours)<br>14 food groups [cereals, vitamin A (VA)-rich vegetables and tubers, white tubers/roots, green leafy vegetables, other vegetables, VA-rich fruits, other fruits, offal, meat, eggs, fish, legumes/nuts/seeds, milk/dairy product, oils/fats]. A point allocated for each group consumed and the index-member dietary diversity score (IDDS) was the sum of these	2007 score mean 2007 (n=2970): 5.7 ± 1.7<br>2008 score mean (n=2962): 5.2 ± 1.5<br>P < 0.0001<br>Tertiles 2007 (n=2970): low 24.2%, medium 44.9%, high 30.9%.<br>Tertiles 2008 (n=2962): low 31.1%, medium 52.4%, high 16.5%<br>P < 0.0001<br>Raw and adjusted changes in dietary diversity between 2007 and 2008:<br>Model 0:<br>2007: 5.59 ± 0.08, 2008: 5.20 ± 0.07, <0.0001<br>Model 1:<br>2007: 5.63 ± 0.08, 2008: 5.20 ± 0.07, <0.0001<br>Model 2:<br>2007: 5.65 ± 0.08, 2008: 5.15 ± 0.07, <0.0001	Selection total: 3<br>Comparability total: 2<br>Outcomes total: 2<br>**Total score: 7**

					points, theoretically ranging from 0 (no food consumed the previous day) to 14 (maximum diversity).		
Norte et al, 2019	National: Spain<br>Local: n/a	Spanish National Health Survey. Stratified random sampling (tri-stage sample). Data were collected by personal interviews and all the information included was self-reported. Adults only. 49.1% (2006) and 50.1% (2011).<br>N= 28,296 in 2006/2007 and 20,920 in 2011/12	Serial cross-sectional. Logistic regression adjusted by age and sex.	Exposure: Commence-ment of GR (time)<br>Time point 1: 2006/2007 (=28,296)<br>Time point 2: 2011/2012 (n=20,920)	Odds of poor diet, using Spanish Healthy Eating Index (odds of score of <51). Scored out of 100 based on 10 equally weighted components: consumption of cereals and derivatives; vegetables; fruits; milk and derivatives; meat, fish and eggs; pulses; sausages and cold meats; sweets; soft drinks with sugar; and variety of the diet, based on the Spanish Healthy Eating Guide. This was then made into a categorical variable with a 'good' diet as a	**SHEI by education level (university = ref):**<br><u>2006</u><br>Secondary AOR: 1.79 (95% CI 1.45 - 2.22), p<0.001<br>No studies/primary AOR: 2.16 (95% CI 1.76–2.63), p=0.227<br><u>2012</u><br>Secondary AOR: 1.96 (95% CI 1.42–2.71), p<0.001<br>No studies/primary AOR: 2.86 (95% CI 2.12–3.87), p<0.001<br>**SHEI by social class (skilled manual work = ref) :**<br><u>2006</u><br>Unskilled manual work AOR: 1.54 (95% CI 1.32–1.79), p<0.001<br><u>2012</u><br>Unskilled manual work AOR: 1.81 (95% CI 1.50–2.18), p<0.001<br>**SHEI by employment situation (paid worker = ref)**<br><u>2006</u><br>Unemployed AOR: 0.74 (95% CI 0.62–0.90), p<0.005<br>Homemakers AOR: 0.36 (95% CI 0.28–0.47), p<0.001<br><u>2012</u>	Selection total: 3<br>Comparability total: 1<br>Outcomes total: 2<br>**Total score: 6**

					score over 80, a 'needs improvement' diet as 51-80, and a 'poor' diet as less than 50.	Unemployed AOR: 1.27 (95% CI 1.05–1.55), p<0.005<br>Homemakers AOR: 1.11 (95% CI 0.85–1.45), p=0.443	
Todd, 2014	National: USA<br>Local: n/a	National Health and Nutrition Examination Survey (NHANES – Consumer Behaviour Adult follow-up survey).<br>Adults born between 1946 and 1985 (aged 20-59 in 2005-6 and 25-64 2009-10)<br>N=9829	Serial Cross-sectional.<br>Weighted means reported and Person Chi-Squared test accounting for complex survey design.	Exposure: Commence-ment of GR (time)<br>Time point 1: 2005-2006 (n=3,014)<br>Time point 2: 2007-2008 (n= 3,294)<br>Time point 3: 2009-2010 (n=3,531)	Self-reported dietary quality (excellent, very good, good, fair or poor).<br>Collected in the Diet Behaviour dataset.	**Born 1946-85**<br>Excellent - 2007-08: 7.6%, 2009-10: 7.9%<br>Very good - 2007-08: 22.1%, 2009-10: 21.2%<br>Good - 2007-08: 40.1%, 2009-10: 44.0%<br>Fair - 2007-08: 23.0%, 2009-10: 22.5%<br>Poor - 2007-08: 7.1%, 2009-10: 4.5%<br>Pearson Chi-squared = 2.63, p=0.053<br>**Born before 1946**<br>Excellent - 2007-08: 15.3%, 2009-10: 16.3%<br>Very good - 2007-08: 30.0%, 2009-10: 31.4%<br>Good - 2007-08: 38.2%, 2009-10: 40.4%<br>Fair - 2007-08: 13.2%, 2009-10: 9.3%<br>Poor - 2007-08: 3.3%, 2009-10: 2.5%<br>Pearson Chi-squared = 1.565, p=0.194	Selection total: 3<br>Comparability total: 2<br>Outcomes total: 1<br>**Total score: 6**
Food Groups							
Alves, 2019<br>Ref:	National: Portugal<br>Local: n/a	National Health Interview Survey.<br>Regional and multistage stratified sampling.<br>Adults aged 25-79, 46% male.	Serial cross-sectional.<br>Used logistic regression to model consumption as a function of year, controlling for age, sex and education.	Exposure: Commence-ment of GR (time)<br>Time point 1: 2005/2006<br>Time point 2: 2014	Dichotomised consumption of food on the previous day (yes/no) for meat, soup, fish, potatoes/ rice/ pasta, bread, legumes, sweets,	Meat: 2005/06: 79% yes, 2014: 29% AOR: 1.004<br>Soup: 2005/6: 68% yes, 2014: 64%, AOR: 0.779 (P<0.01)<br>Fish: 2005/6: 52% yes, 2014: 49%, AOR: 0.811 (P<0.01)<br>Potatoes/ rice/ pasta: 2005/6: 90% yes, 2014: 89%, AOR: 0.973	Selection total: 3<br>Comparability total: 1<br>Outcomes total: 1<br>**Total score: 5**

		N=43,273 (28,144 in 2005/2006).			fruits and vegetables.	Bread: 2005/6: 93% yes, 2014: 92%, AOR: 0.912<br>Legumes: 2005/6: 27% yes, 2014: 32%, AOR: 1.336 (P<0.01)<br>Sweets: 2005/6: 26% yes, 2014: 37%, AOR: 1.536<br>Fruits: 2005/6: 82% yes, 2014: 73%, AOR: 0.502 (P<0.01)<br>Vegetables: 2005/6: 78% yes, 2014: 52%, AOR: 0.446 (P<0.01).<br>**Only significant change across subpopulation was for potatoes/rice/pasta in ages 40-64:**<br>All groups: 0.95<br>No/pre-primary education: 0.92<br>Primary/secondary education: 1.15<br>Tertiary education: 0.89<br>*Note that Alves considered these changes to be reflective of long-term declines in the Mediterranean diet rather than due to the GR.*	
Antelo, 2017	National: Spain<br>Local: n/a	National household budget survey. Households with an active breadwinner over 16. Male dominated households went from a mean of 0.81 to 0.70.<br>N= 12,480 in 2006 and 14,215 in 2013.	Longitudinal survey. Used Propensity Score Matching with Gaussian kernel methods and a difference-in-difference approach to examine the impact of the economic crisis on Unemployment Effect on Food Expenditure (UEFE). Controlled for age, sex, household size,	Exposure: time (boom and crisis periods).<br>Time point 1: 2006 (boom)<br>Time point 2: 2013 (crisis)<br>Investigated Unemployment Effect on Food Expenditure in boom and crisis periods	Data on food expenditure by household collected for two weeks.<br>Expenditure on: bread, cereals, rice and pasta; meat; fish; milk, cheese and eggs; oils and fats; fruits; vegetables, pulses, potatoes and other root crops; sugar, jam,	DiD estimates with Gaussian kernel Matching for crisis impact/UEFE:<br>Bread, cereals, rice and pasta crisis effect: −0.164 (−6.13) (p at 1% level)<br>Meat crisis effect:<br>− 0.158 (− 3.55) (p at 1% level)<br>Fish crisis effect:<br>− 0.192 (− 2.91) (p at 1% level)<br>Milk, cheese and eggs crisis effect:<br>- 0.082 (- 2.19) (p at 5% level)<br>Oils and fats crisis effect:<br>− 0.073 (− 1.03)<br>Fruits crisis effect:<br>− 0.304 (- 6.20) (p at 1% level)	Selection total: 3<br>Comparability total: 2<br>Outcomes total: 2<br>**Total score: 7**

			marital status, education, home ownership, residential area, other houses, number of home-cooked meals, and region.	(unemployment rate was 4.8% in 2006 and 15.5% in 2013).	honey, chocolate, sweets and ice cream.	Vegetables, pulses, potatoes and other root crops crisis effect:<br>− 0.173 (− 4.10) (p at 1% level)<br>Sugar, jam, honey, chocolate, sweets and ice cream crisis effect:<br>− 0.106 (− 1.83) (p at 10% level)	
Asgeirs-dottir, 2014	National: Iceland<br>Local: n/a	(Icelandic) Health and Wellbeing survey, weighted and using stratified random sampling. Adults aged 18 – 79. Unweighted: 48.9% male completed 2009 survey, 46.6% when weighted. N = 7688. Response rate 60.8% in 2007 and 69.3% in 2009.	Longitudinal.<br>Used fixed effects models using pooled data. Time-varying covariates are married, cohabiting, lives with adult other than partner, and lives in rural area.	Exposure: Commence-ment of GR (time)<br>Time point 1: 2007<br>Time point 2: 2009	Outcome variables: daily sugared soft drink, daily sweets, weekly consumption of fast food (either at a fast food restaurant or by taking home prepared foods), daily consumption of fruit or berries, daily consumption of cooked or raw vegetables.	Excluding cases with missing data (n=5616) and including time-varying covariates:<br>Soft drink: - 0.016 (p<0.01)<br>Sweets: - 0.023 (p<0.01)<br>Fast food: - 0.048 (p<0.01)<br>Fruit/berries: - 0.018 (p<0.1)<br>Vegetables: - 0.028 (p<0.01)<br>Working age subset:<br>Soft drink: - 0.017 (p<0.01)<br>Sweets: - 0.024 (p<0.01)<br>Fast food: - 0.067 (p<0.01)<br>Fruit/berries: - 0.013 (p<0.1)<br>Vegetables: - 0.025 (p<0.01)	Selection total: 3<br>Comparability total: 2<br>Outcomes total: 2<br>**Total score: 7**
Asgeirs-dottir, 2016	National: Iceland<br>Local: n/a	(Icelandic) Health and Wellbeing survey. Stratified random sampling. Aged 18 – 79 but generally	Longitudinal.<br>Fixed effects models, covariates included married, cohabiting, lives with adult other than partner, lives in rural area, and homeowner.	Exposure: Commence-ment of GR (time)<br>Time point 1: 2007<br>Time point 2: 2009<br>Time point 3:	Outcome variables: daily sugared soft drink, daily sweets, weekly consumption of fast food (either at a fast food restaurant or by	Analysis sample with time-varying covariates:<br>**Soft drinks:**<br>Effect of 2009 indicator: -0.013 (p < 0.01)<br>Effect of 2012 indicator: - 0.027 (p < 0.10)<br>p-Value for difference between 2009 and 2012: 0.056	Selection total: 3<br>Comparability total: 2<br>Outcomes total: 2<br>**Total score: 7**

		older than Icelandic population so sample weights applied. 46% of those who completed all three surveys were male.<br>33% of those who completed the 2007 survey also completed the 2009 and 2012 surveys, N=3238.		2012	taking home prepared foods), daily consumption of fruit or berries.	R-squared: 0.009<br>**Sweets:**<br>Effect of 2009 indicator: -0.021 (p < 0.10)<br>Effect of 2012 indicator: - 0.008<br>p-Value for difference between 2009 and 2012: 0.109<br>R-squared: 0.008<br>**Fast food:**<br>Effect of 2009 indicator: - 0.053 (p < 0.10)<br>Effect of 2012 indicator: - 0.071 (p < 0.10)<br>p-Value for difference between 2009 and 2012: 0.126<br>R-squared: 0.023<br>**Fruit/berries:**<br>Effect of 2009 indicator: -0.029 (p < 0.05)<br>Effect of 2012 indicator: 0.049 (p < 0.1)<br>p-Value for difference between 2009 and 2012: <0.001<br>R-squared: 0.015	
Bartoll, 2015	National: Spain<br>Local: n/a	Spanish National Health Survey<br>Adults aged 25-64.<br>N=47,156.<br>Response rates among eligible units in the last three waves were 77%, 70%, 72%	Repeated cross-sectional.<br>Before-after model (linear probability regression model).<br>All models are adjusted by age, age^2, marital status, region of residence (autonomous community), type of residential area (rural/urban),	Exposure: Commence-ment of GR (time)<br>Time point 1: 2001 (n=9,252)<br>Time point 2: 2003/2004 (n=10,840)<br>Time point 3: 2006/2007 (n=15,470)	Daily consumption (yes/no) of vegetables, fruit. Binary variable for consumption three or more times a week of legumes, fish, meat, processed meat (eg. salami, sausages), sweet	**Vegetables:**<br><u>MEN</u><br>*Overall:* -0.002 (0.0141)<br>*Employment status*<br>Employed: 0.009 (0.0150), unemployed -0.063 (0.0448), p (a) 0.004<br>*Education level*<br>University -0.004 (0.0316), High secondary 0.038 (0.0289), Lower secondary or primary - 0.013 (0.0194), without any qualification - 0.068 (0.0734), p (b) 0.385.	Selection total: 3<br>Comparability total: 2<br>Outcomes total: 2<br>**Total score: 7**

			occupation and linear time trend.<br>(a) Significance of t-test of the interaction between economic recession dummy and employment status<br>(b) Significance of likelihood ratio of the model with and without interaction between economic recession and education level.<br>Robust standard errors in parentheses.	Time point 4: 2011/2012 (n=11,594)	foods (eg. jam, cookies).	WOMEN<br>*Overall:* −0.024 (0.0150)<br>*Employment status*<br>Employed: −0.017 (0.0166), unemployed −0.043 (0.0364), p (a) 0.065<br>*Education level*<br>University −0.013 (0.0287), High secondary 0.034 (0.0313), Lower secondary or primary −0.030 (0.0222), without any qualification −0.271 (0.0795) p<0.01, p (b) 0.004<br>**Fruit:**<br>MEN<br>*Overall*: −0.091 (0.0146) p<0.01<br>*Employment status*<br>Employed: −0.074 (0.0154) p<0.01, unemployed −0.121 (0.0498) p<0.05, p (a) 0.041<br>*Education level*<br>University − 0.045 (0.0319), High secondary − 0.061 p<0.05 (0.0289), lower secondary or primary − 0.114 (0.02040) p<0.01, without any qualification − 0.218 p<0.01 (0.0729), p (b) 0.060.<br>WOMEN<br>*Overall*: − 0.079 (0.0144) p<0.01<br>*Employment status*<br>Employed: −0.071 (0.0159) p<0.01, unemployed −0.106 (0.0345) p<0.01, p (a) 0.315<br>*Education level*<br>University −0.058 (0.0268) p<0.05, High secondary −0.048 (0.0301), lower secondary or primary −0.092 (0.0216)	

						p<0.01, without any qualification −0.151 (0.0781) p<0.1, p (b) 0.429<br>**Legumes:**<br>MEN<br>*Overall:* 0.034 (0.0135) p<0.05<br>*Employment status*<br>Employed: 0.038 (0.0142) p<0.01, unemployed −0.074 (0.0479),<br>p (a) 0.041<br>*Education level*<br>University 0.040 (0.0281),<br>High secondary 0.010 (0.0280),<br>Lower secondary or primary 0.038 (0.0194) p<0.05, without any qualification −0.035 (0.0681),<br>p (b) 0.621.<br>WOMEN<br>*Overall*: 0.043 (0.0136) p<0.01<br>*Employment status*<br>Employed: 0.039 (0.0151) p<0.05, unemployed 0.057 (0.0325) p<0.1,<br>p (a) 0.392<br>*Education level*<br>University 0.051 (0.0256) p<0.1,<br>High secondary 0.038 (0.0274),<br>Lower secondary or primary 0.045 (0.0207) p<0.05, without any qualification −0.105 (0.0781),<br>p (b) 0.314<br>**Fish:**<br>MEN<br>*Overall*: −0.001 (0.0144)<br>*Employment status*<br>Employed: 0.012 (0.0152), unemployed −0.068 (0.0501),<br>p (a) 0.055	

						Education level<br>University −0.020 (0.0319),<br>High secondary 0.001 (0.0293),<br>Lower secondary or primary 0.004 (0.0200), without any qualification −0.003 (0.0728), p (b) 0.937<br>WOMEN<br>*Overall:* −0.000 (0.0149)<br>*Employment status*<br>Employed: 0.002 (0.0166),<br>unemployed −0.007 (0.0354),<br>p (a) 0.437<br>*Education level*<br>University 0.042 (0.0287),<br>High secondary −0.006 (0.0307),<br>Lower secondary or primary −0.013 (0.0222), without any qualification −0.048 (0.0840), p (b) 0.570<br>**Meat:**<br>MEN<br>*Overall*: −0.097 (0.0125) p<0.01<br>*Employment status*<br>Employed: −0.093 (0.0131) p<0.01,<br>unemployed −0.110 (0.0457) p<0.05,<br>p (a) 0.229<br>*Education level*<br>University −0.093 (0.0281) p<0.01,<br>High secondary −0.079 (0.0251) p<0.01, Lower secondary or primary −0.092 (0.0175) p<0.01, without any qualification −0.268 (0.0705) p<0.01,<br>p (b) 0.092<br>WOMEN<br>*Overall:* −0.100 (0.0135) p<0.01<br>*Employment status*	

						Employed: −0.097 (0.0149) p<0.01, unemployed −0.109 (0.0328) p<0.01, p (a) 0.567<br>*Education level*<br>University −0.093 (0.0258) p<0.01, High secondary −0.089 (0.0287) p<0.01, Lower secondary or primary −0.104 (0.0197) p<0.01, without any qualification −0.211 (0.0808) p<0.01, p (b) 0.541<br>**Cold Meat:**<br><u>MEN</u><br>*Overall:* −0.047 (0.0148) p<0.01<br>Employment status<br>Employed: −0.047 (0.0157) p<0.01, unemployed −0.003 (0.0496), p (a) 0.341<br>*Education level*<br>University −0.085 (0.0323) p<0.01, High secondary −0.026 (0.0304), Lower secondary or primary −0.036 (0.0205) p<0.1, without any qualification −0.173 (0.0806) p<0.05, p (b) 0.203<br><u>WOMEN</u><br>*Overall*: −0.037 (0.0145) p<0.05<br>*Employment status*<br>Employed: −0.029 (0.0161) p<0.1, unemployed −0.065 (0.0344) p<0.1, p (a) 0.836<br>*Education level*<br>University −0.054 (0.0276) p<0.1, High secondary −0.058 (0.0297) p<0.1, Lower secondary or primary −0.019 (0.0218), without any qualification −0.099 (0.0777), p (b) 0.558	

						**Sweets:**<br><u>MEN</u><br>*Overall*: 0.012 (0.0149)<br>*Employment status*<br>Employed: 0.007 (0.0158),<br>Unemployed 0.011 (0.0491),<br>p (a) 0.272<br>*Education level*<br>University −0.017 (0.0325),<br>High secondary 0.025 (0.0306),<br>Lower secondary or primary 0.031 (0.0208), without any qualification −0.169 (0.0785) p<0.05, p (b) 0.067<br><u>WOMEN</u><br>*Overall:* 0.001 (0.0152)<br>*Employment status*<br>Employed: 0.002 (0.0168),<br>unemployed −0.004 (0.0361),<br>p (a) 0.921<br>*Education level*<br>University 0.024 (0.0289),<br>High secondary −0.011 (0.0312),<br>Lower secondary/ primary 0.001 (0.0226), without any qualification −0.039 (0.0848), p (b) 0.806	
Bonaccio et al, 2014<br>Ref:	National: Italy<br>Local: the Molise region	Moli-Sani study. Adults aged over 35 (mean age 54.4); percentage male: 47.30%. Randomly recruited.<br>Total n=21,001.	Serial cross-sectional study of participants recruited before and after the recession. Means and p-values of intake in grams/day adjusted for age and sex.	Exposure: Commence-ment of GR (time).<br>Time point 1: Recruited in 2005-2006 (n=6999)<br>Time point 2: Recruited in 2007-2010	Animal proteins, vegetarian proteins, animal fats and vegetable fats in grams/day (means + SD) using Italian EPIC food frequency questionnaire.	Animal proteins:<br>2005/6: 55.1 (18.7), 2007-2010: 56.4 (17.0), p-value <0.0001<br>Vegetarian proteins:<br>2005/6: 29.6 (11.1), 2007-2010: 28.7 (9.9), p-value <0.0001<br>Animal fats:<br>2005/6: 44.2 (18.8), 2007-2010: 45.3 (17.0), p-value <0.0001<br>Vegetarian fats:	Selection total: 3<br>Comparability total: 2<br>Outcomes total: 2<br>**Total score: 7**

				(n=14,002)		2005/6: 33.1 (11.8), 2007-2010: 32.5 (10.6), p-value <0.0001	
Colman & Dave, 2018	National: USA<br>Local: n/a	National Longitudinal Survey of Youth-1979.<br>A nationally representative sample of 12,686 young men and women who were 14–22 years old when they were first surveyed in 1979 (food and drink data collected from 2008).<br>N=7100 in 2014.	Longitudinal.<br>Separate fixed-effect regressions controlling for age, marital status, state of residence, and month.	Exposure: Unemploy-ment (own).<br>Time point 1: 2008<br>Time point 2: 2010<br>Time point 3: 2012<br>Time point 4: 2014	Fast food per week (In the past seven days, how many times did you eat food from a fast food restaurant such as McDonalds, Kentucky Fried Chicken, Pizza Hut, or Taco Bell?), snacks per week, soft drinks per week	**Fast food:**<br>MEANS: 2008: 1.7, 2010: 1.5, 2012: 1.5, 2014: 1.3<br>EFFECTS OF UNEMPLOYMENT BY REASON FOR UNEMPLOYMENT:<br>Nonemployed:<br>All: −0.37 (0.070) (significant at 1%),<br>Non-movers: −0.34 (0.084) (significant at 1%),<br>Unemployed:<br>All: −0.49 (0.14) (significant at 1%),<br>Non-movers: −0.44 (0.16) (significant at 1%),<br>Laid off:<br>All: −0.29 (0.22),<br>Non-movers: −0.34 (0.27)<br>Plant closed:<br>All: −0.50 (0.44),<br>Non-movers: −0.59 (0.51)<br>**Snacks:**<br>MEANS: 2008: 6.3, 2010: 6.2, 2012: 6.1, 2014: 6.6<br>EFFECTS OF UNEMPLOYMENT BY REASON FOR UNEMPLOYMENT:<br>Nonemployed:<br>All: 0.18 (0.25),<br>Non-movers 0.18 (0.28),<br>Unemployed:<br>All: −0.26 (0.43),<br>Non-movers: −0.11 (0.51),<br>Laid off:<br>All: 0.45 (0.59),<br>Non-movers: 0.91 (0.67)<br>Plant closed:	Selection total: 4<br>Comparability total: 1<br>Outcomes total: 2<br>**Total score: 7**

						All: −0.14 (1.06),<br>Non-movers: −0.66 (1.18)<br>**Soft drinks:**<br>MEANS: 2008: 3.9, 2010: 3.4, 2012: 3.1, 2014: 2.9<br>EFFECTS OF UNEMPLOYMENT BY REASON FOR UNEMPLOYMENT:<br>Nonemployed:<br>All: −0.15 (0.19),<br>Non-movers −0.22 (0.22),<br>Unemployed:<br>All: 0.0001 (0.40),<br>Non-movers: −0.18 (0.46),<br>Laid off:<br>All: −0.74 (0.50),<br>Non-movers: −0.99 (0.61)<br>Plant closed:<br>All: 0.061 (0.60),<br>Non-movers: −0.15 (0.70)	
Çirakli & Yildirim, 2019	National: Turkey<br>Local: n/a	OECD data.	Used ARDL bounds testing and cointegration analysis including OLS: unit root tests, Augmented Dickey-Fuller Test, Unrestricted Error Correction Model using OLS and Wald test, estimation of long-term coefficients and creation of Error Correction Model.	Exposure: Commence-ment of GR (time), using real GDP, unemploy-ment rates, inflation rate as indicators of crises.<br>Time: 1974-2015 (42 time points), covering economic crises in 1994, 2001, 2009).	Used OECD data on annual per capita vegetable and fruit consumption (kg)	Result of ADF Unit Root Test:<br>level values t: -2.43, p 0.138; first difference values: t: -7.16 (significant at 1% level), p < 0.001.<br>Result of ARDL correction model:<br>Change D 2009: coefficient: 0.051, t: 2.139, p: 0.042<br>(significant positive impact on fruit and vegetable consumption).	Selection total: 2<br>Comparability total: 1<br>Outcomes total: 1<br>**Total score: 4**

Dave et al., 2012	National: USA<br>Local: n/a	Behavioural Risk Factor Surveillance System. Adults aged between 26-58.<br>N (fruit) = 1,354,093<br>N (fruit juice) = 1,357,023<br>N (carrots) = 1,344,014<br>N (green salad) = 1,357,023<br>N (vegetables) = 1,349, 973<br>N (snacks) = 56,376<br>N hamburgers) = 56,742<br>N (hot dogs) = 56,586<br>N (French fries) = 56,467<br>N (fried chicken) = 56,528<br>N (doughnuts) = 56,428	Serial cross-sectional. Reduced-form cross-equation (fixed effects) estimates of the average effect of state unemployment on healthy and unhealthy food consumption seemingly unrelated regression. Controlled for gender, education, age, marital status, ethnicity and state indicators.	Exposure: Area-level unemploy-ment rates.<br>Time: 1990 – 2009 (excluding 2004, 2006 and 2008).	How often do you eat (FOOD) with options of times per day, week, month, or year for fruit, fruit juice, carrots, green salad, vegetables, snacks, hamburgers, hot dogs, French fries, fried chicken, and doughnuts.	**Fruit:**<br>State unemployment: −0.1494 (0.0451) ($p<0.01$)<br>Male: −6.6536 (0.2100) ($p<0.01$)<br>Some high school: −2.9580 (0.4090) ($p<0.01$)<br>High School: −1.5104 (0.4957) ($p<0.01$)<br>Some college: 1.2394 (0.5265) ($p<0.05$)<br>College: 6.3012 (0.5294) ($p<0.01$)<br>Age: −0.1147 (0.0316) ($p<0.01$)<br>Age squared: 0.0033 (0.0004) ($p<0.01$)<br>Black: 0.3630 (0.3553)<br>Hispanic: 3.2468 (0.4478) ($p<0.01$)<br>Other race: 1.0008 (0.5885) ($p<0.1$)<br>**Fruit juice:**<br>State unemployment: −0.1406 (0.0492) ($p<0.01$)<br>Male: 1.9020 (0.1619) ($p<0.01$)<br>Some high school: −1.5162 (0.3097) ($p<0.01$)<br>High School: −1.3282 (0.4140) ($p<0.01$)<br>Some college: −0.0554 (0.4594)<br>College: 0.8333 (0.4808) ($p<0.1$)<br>Age: −0.8824 (0.0273) ($p<0.01$)<br>Age squared: 0.0098 (0.0003) ($p<0.01$)<br>Black: 7.3910 (0.2541) ($p<0.01$)<br>Hispanic: 5.6835 (0.4354) ($p<0.01$)<br>Other race: 3.7315 (0.3987) ($p<0.01$)<br>**Carrots:**<br>State unemployment: −0.1051 (0.0222) ($p<0.01$)<br>Male: −1.7989 (0.0555) ($p<0.01$)<br>Some high school: −1.2052 (0.2242) ($p<0.01$)<br>High School: −0.8552 (0.2666) ($p<0.01$)<br>Some college: 0.1453 (0.2863)	Selection total: 3<br>Comparability total: 2<br>Outcomes total: 2<br>**Total score: 7**

						College: 1.0244 (0.2945) (p<0.01)<br>Age: 0.2381 (0.0161) (p<0.01)<br>Age squared: −0.0020 (0.0002) (p<0.01)<br>Black: −0.8415 (0.1054) (p<0.01)<br>Hispanic: 1.6735 (0.1748) (p<0.01)<br>Other race: 0.7720 (0.2129) (p<0.01)<br>**Green salad:**<br>State unemployment: −0.0894 (0.0321) (p<0.01)<br>Male: −2.9886 (0.0687) (p<0.01)<br>Some high school: 0.1686 (0.1331)<br>High School: 1.5524 (0.1578) (p<0.01)<br>Some college: 3.3623 (0.1964) (p<0.01)<br>College: 5.2470 (0.2051) (p<0.01)<br>Age: 0.2560 (0.0196) (p<0.01)<br>Age squared: −0.0015 (0.0002) (p<0.01)<br>Black: −0.7445 (0.1536) (p<0.01)<br>Hispanic: 1.5114 (0.2731) (p<0.01)<br>Other race: 0.0837 (0.2419)<br>**Vegetables:**<br>State unemployment: −0.0997 (0.0712)<br>Male: −6.8483 (0.2285) (p<0.01)<br>Some high school: 2.1519 (0.3098) (p<0.01)<br>High School: 4.4294 (0.3490) (p<0.01)<br>Some college: 8.3667 (0.3556) (p<0.01)<br>College: 12.2960 (0.3824) (p<0.01)<br>Age: 0.0737 (0.0266) (p<0.01)<br>Age squared: −0.0004 (0.0003)<br>Black: −2.8057 (0.4086) (p<0.01)<br>Hispanic: −7.4141 (0.7634) (p<0.01)<br>Other race: 0.7997 (0.7680)<br>**Snacks:**	

						State unemployment: 0.1860 (0.1061) ($p<0.1$)<br>Male: 0.6343 (0.1160) ($p<0.01$)<br>Some high school: 2.0369 (0.4282) ($p<0.01$)<br>High School: 1.6495 (0.4167) ($p<0.01$)<br>Some college: 1.0825 (0.4678) ($p<0.05$)<br>College: 0.5646 (0.4752)<br>Age: 0.0768 (0.0777)<br>Age squared: −0.0026 (0.0009) ($p<0.01$)<br>Black: −0.7003 (0.3244) ($p<0.05$)<br>Hispanic: −1.6512 (0.3306) ($p<0.01$)<br>Other race: −1.4626 (0.3809) ($p<0.01$)<br>**Hamburgers:**<br>State unemployment: 0.0370 (0.0697)<br>Male: 1.8570 (0.0766) ($p<0.01$)<br>Some high school: 0.4917 (0.3082)<br>High School: 0.0888 (0.2864)<br>Some college: −0.3641 (0.2857)<br>College: −1.4603 (0.3362) ($p<0.01$)<br>Age: −0.0280 (0.0294)<br>Age squared: −0.0008 (0.0004) ($p<0.05$)<br>Black: −0.6633 (0.1932) ($p<0.01$)<br>Hispanic: −0.8492 (0.1274) ($p<0.01$)<br>Other race: −0.1696 (0.3522)<br>**Hot dogs:**<br>State unemployment: 0.0766 (0.1567)<br>Male: 3.3519 (0.1798) ($p<0.01$)<br>Some high school: 1.0307 (0.4678) ($p<0.05$)<br>High School: −0.3194 (0.3365)<br>Some college: −1.1975 (0.3859) ($p<0.01$)<br>College: −2.4662 (0.3513) ($p<0.01$)	

						Age: −0.2279 (0.0775) (p<0.01)<br>Age squared: 0.0014 (0.0009)<br>Black: −0.8830 (0.2041) (p<0.01)<br>Hispanic: −0.8022 (0.2380) (p<0.01)<br>Other race: −0.6258 (0.4391)<br>**French fries**<br>State unemployment: 0.0116 (0.1021)<br>Male: 2.3808 (0.1188) (p<0.01)<br>Some high school: −0.5574 (0.3325)<br>High School: −1.3748 (0.3650) (p<0.01)<br>Some college: −2.0336 (0.3693) (p<0.01)<br>College: −2.7835 (0.3672) (p<0.01)<br>Age: −0.1912 (0.0288) (p<0.01)<br>Age squared: 0.0007 (0.0003) (p<0.05)<br>Black: −0.5871 (0.2280) (p<0.05)<br>Hispanic: −0.1536 (0.1992)<br>Other race: −0.0350 (0.1671)<br>**Fried chicken:**<br>State unemployment: 0.0782 (0.0591)<br>Male: 0.9190 (0.1060) (p<0.01)<br>Some high school: 0.0258 (0.3215)<br>High School: −0.8157 (0.1851) (p<0.01)<br>Some college: −1.2040 (0.2094) (p<0.01)<br>College: −1.8284 (0.2314) (p<0.01)<br>Age: 0.0371 (0.0370)<br>Age squared: −0.0006 (0.0005)<br>Black: 3.1662 (0.1238) (p<0.01)<br>Hispanic: 1.0068 (0.1875) (p<0.01)<br>Other race: 1.4597 (0.2047) (p<0.01)<br>**Doughnuts:**<br>State unemployment: 0.1079 (0.1434)<br>Male: 0.5286 (0.1785) (p<0.01)<br>Some high school: −0.0171 (0.4621)<br>High School: −0.8243 (0.4170) (p<0.1)	

						Some college: −1.0546 (0.5096) (p<0.05)<br>College: −1.1150 (0.4393) (p<0.05)<br>Age: 0.1816 (0.0998) (p<0.1)<br>Age squared: −0.0024 (0.0012) (p<0.1)<br>Black: −1.1121 (0.2311) (p<0.01)<br>Hispanic: −1.2748 (0.3503) (p<0.01)<br>Other race: −1.6652 (0.5399) (p<0.01)	
Di Pietro, 2018	National: Italy<br>Local: n/a	Italian Multipurpose Household Survey on Everyday Life Issues.<br>Age 25-54.<br>Maximum n=189.631	Serial cross-sectional. Reduced form demand function (linear probability models estimated with OLS). All equations include fixed effects for region and year in addition to controls for education, age, marital status and gender. Eq. (2) contains region-specific linear time trends. Eq. (3) contains linear time trends in age group by gender specific to each region and fixed effects for the interaction between year and region.	Two measures of unemploy-ment rate are used: a general unemploy-ment and unemploy-ment rate by age group and gender is used. Data on both unemploy-ment rates come from the ISTAT.<br>Time: 2005-2012	Dichotomous variable - whether respondent regularly consumes at least 5 daily servings of fruits and/or vegetables or snacks high in salt (eg. French fries, popcorn).	**Fruit & vegetables:**<br>Eq. 1 general unemployment rate: 0.0048 (0.0036)<br>Eq. 2 general unemployment rate: 0.0052 (0.0036)<br>Eq. 3: unemployment rate by gender and age group:<br>− 0.0017 (0.0007) (p<0.05)<br>n= 160,060<br>**Snacks:**<br>Eq. 1 general unemployment rate:<br>− 0.0003 (0.0007)<br>Eq. 2 general unemployment rate:<br>− 0.0007 (0.0008)<br>Eq. 3: unemployment rate by gender and age group:<br>− 0.0006 (0.0003) (p<0.5)<br>n= 158,844	Selection total: 3<br>Comparability total: 2<br>Outcomes total: 2<br>**Total score: 7**
Díaz-Méndez & García-Espejo, 2019	National: Spain<br>Local: n/a	National Health Survey.<br>Adults aged 16 and above.	Serial Cross-sectional.<br>Logistic regression.<br>Age, sex, education, parental occupation	Exposure: Commence-ment of GR (time)<br>Time point 1:	Daily consumption of fruit and vegetables (Y/N), three times a	**Fruit:**<br>Time Trend 2006-2011: diverging from the guidelines, with frequency of consumption falling.<br>Logistic Regression:	Selection total: 3<br>Comparability total: 2

		2006 (n=29,478) 2011 n=21,007). All data weighted using original weighting variable for each year.	(for SES), size of community, nationality, marital status, presence of minors in the household, employment situation, BMI, being on a diet and physical activity included in model.	2006<br>Time point 2: 2011	week or more consumption of meat or fish, less than once a week/seldom or never (vs. more frequent) consumption of cookies, pastries, sweetened cereals, jelly and candy (based on healthy diet guidelines).	Sex (base: woman) 2006: -0.513 (p<0.01). 2011: -0.412 (p<0.01)<br>Age (base: under 30 years old)<br>30-44: 2006: 0.385 (p<0.01). 2011: 0.295 (p<0.01)<br>45-59: 2006: 1.018 (p<0.05). 2011: 0.858 (p<0.01)<br>60-74: 2006: 1.571 (p<0.01). 2011: 1.198 (p<0.01)<br>Over 74: 2006: 1.770 (p<0.01). 2011: 1.401 (p<0.01)<br>Education level (base: none)<br>Primary education: 2006: 0.112, 2011: 0.272 (p<0.01)<br>General secondary: 2006 0.122 (p<0.1), 2011: 0.269 (p<0.01)<br>Vocational training: 2006 0.187 (p<0.05), 2011: 0.263 (p<0.01)<br>University: 2006 0.282 (p<0.01), 2011: 0.427 (p<0.01)<br>Unemployed (base: employed)<br>2006: -0.184 (p<0.01), 2011: -0.213 (p<0.01)<br>Social class of breadwinner (base: non-skilled workers)<br>Public administration directors and directors of companies with 10 or more workers and professionals associated with a graduate degree: 2006: 0.192 (p<0.05), 2011: 0.254 (p<0.01)<br>Executives of public administration and of companies with fewer than 10 workers, professionals associated with university degrees:<br>2006: 0.079, 2011: 0.178 (p<0.05)	Outcomes total: 1<br>**Total score: 6**

						Administrative support and professionals<br>2006: 0.100 (p<0.1), 2011: 0.122 (p<0.1)<br>Skilled manual workers<br>2006: 0.076, 2011: 0.107 (p<0.1)<br>Semi-skilled manual workers<br>2006: 0.035, 2011: 0.102 (p<0.1)<br>**Vegetables:**<br>Time Trend 2006-2011: diverging from the guidelines, with frequency of consumption falling.<br>Logistic Regression:<br>Sex (base: woman): 2006: -0.510 (p<0.01). 2011: -0.507 (p<0.01)<br>Age (base: under 30 years old)<br>30-44: 2006: 0.276 (p<0.01). 2011: 0.356 (p<0.01)<br>45-59: 2006: 0.628 (p<0.01). 2011: 0.692 (p<0.01)<br>60-74: 2006: 0.768 (p<0.01). 2011: 0.982 (p<0.01)<br>Over 74: 2006: 0.735 (p<0.01). 2011: 1.037 (p<0.01)<br>Education level (base: none)<br>Primary education:<br>2006: 0.031, 2011: 0.253 (p<0.01)<br>General secondary:<br>2006: 0.061, 2011: 0.444 (p<0.01)<br>Vocational training:<br>2006 0.007, 2011: 0.473 (p<0.01)<br>University:<br>2006: 0.168, 2011: 0.604 (p<0.01)<br>Unemployed (base: employed)<br>2006: -0.006, 2011: -0.219 (p<0.01)	

						Social class of breadwinner (base: non-skilled workers)<br>Public administration directors and directors of companies with 10 or more workers and professionals associated with a graduate degree 2006: 0.388 (p<0.01), 2011: 0.385 (p<0.01)<br>Executives of public administration and of companies with fewer than 10 workers, professionals associated with university degrees 2006: 0.286 (p<0.01), 2011: 0.197 (p<0.01)<br>Administrative support and professionals: 2006: 0.233 (p<0.01), 2011: 0.201 (p<0.01)<br>Skilled manual workers: 2006: 0.192 (p<0.01), 2011: 0.153 (p<0.01)<br>Semi-skilled manual workers 2006: 0.204 (p<0.01), 2011: 0.114 (p<0.05)<br>**Meat:**<br>Time Trend 2006-2011: most of the population still within guidelines, but the % of those reducing consumption is on the rise.<br>Logistic Regression:<br>Sex (base: woman) 2006: 0.044, 2011: 0.152 (p<0.01)<br>Age (base: under 30 years old)<br>30-44: 2006: -0.057, 2011: -0.077<br>45-59: 2006: -0.229 (p<0.01). 2011: -0.231 (p<0.01)<br>60-74: 2006: -0.150 (p<0.1). 2011: -0.407 (p<0.01)<br>Over 74: 2006: - 0.206 (p<0.05). 2011:	

						-0.435 (p<0.01)<br>Education level (base: none)<br>Primary education: 2006: 0.221 (p<0.01), 2011: 0.289 (p<0.01)<br>General secondary: 2006: 0.209 (p<0.01), 2011: 0.104 (p<0.1)<br>Vocational training: 2006: 0.170 (p<0.01), 2011: 0.160 (p<0.05)<br>University: 2006 0.319 (p<0.01), 2011: 0.210 (p<0.01)<br>Unemployed (base: employed)<br>2006 -0.080 2011: -0.072<br>Social class of breadwinner (base: non-skilled workers)<br>Public administration directors and directors of companies with 10 or more workers and professionals associated with a graduate degree<br>2006: 0.038, 2011: 0.043<br>Executives of public administration and of companies with fewer than 10 workers, professionals associated with university degrees:<br>2006: 0.060, 2011: -0.023<br>Administrative support and professionals:<br>2006: 0.050, 2011: 0.109 (p<0.05)<br>Skilled manual workers: 2006: 0.152 (p<0.01), 2011: 0.121 (p<0.05)<br>Semi-skilled manual workers:<br>2006: 0.057, 2011: 0.088 (p<0.1)<br>**Fish:**<br>Time Trend 2006-2011: diverging from the guidelines, with frequency of consumption falling.<br>Logistic Regression:	

						Sex (base: woman) 2006: - 0.216 (p<0.01). 2011: - 0.170 (p<0.01)<br>Age (base: under 30 years old)<br>30-44: 2006: 0.398 (p<0.01). 2011: 0.265 (p<0.01)<br>45-59: 2006: 0.720 (p<0.01). 2011: 0.539 (p<0.01)<br>60-74: 2006: 0.939 (p<0.01). 2011: 0.813 (p<0.01)<br>Over 74: 2006: 0.964 (p<0.01). 2011: 0.747 (p<0.01)<br>Education level (base: none)<br>Primary education:<br>2006: 0.350 (p<0.01), 2011: 0.070<br>General secondary:<br>2006: 0.250 (p<0.01), 2011: 0.107 (p<0.1)<br>Vocational training:<br>2006: 0.349 (p<0.001), 2011: 0.076<br>University:<br>2006: 0.423 (p<0.01), 2011: 0.203 (p<0.01)<br>Unemployed (base: employed)<br>2006: - 0.051, 2011: - 0.151 (p<0.01)<br>Social class of breadwinner (base: non-skilled workers)<br>Public administration directors and directors of companies with 10 or more workers and professionals associated with a graduate degree<br>2006: 0.025, 2011: 0.091<br>Executives of public administration and of companies with fewer than 10 workers, professionals associated with university degrees<br>2006: 0.055, 2011: -0.000	

						Administrative support and professionals<br>2006: 0.023, 2011: -0.028<br>Skilled manual workers<br>2006: 0.024, 2011: -0.036<br>Semi-skilled manual workers<br>2006: 0.045, 2011: -0.100<br>**Sweets:**<br>Time trend 2006-2011: Approaching closer to the guidelines with frequency of consumption falling.<br>Logistic Regression:<br>Sex (base: woman)<br>2006: 0.092 ($p<0.01$). 2011: 0.033<br>Age (base: under 30 years old)<br>30-44: 2006: 0.275 ($p<0.01$). 2011 0.252 ($p<0.01$)<br>45-59: 2006: 0.479 ($p<0.01$). 2011: 0.582 ($p<0.01$)<br>60-74: 2006: 0.393 ($p<0.01$). 2011: 0.685 ($p<0.01$)<br>Over 74: 2006: 0.046. 2011: 0.455 ($p<0.01$)<br>Education level (base: none)<br>Primary education: 2006: -0.188 ($p<0.01$), 2011: -0.271 ($p<0.01$)<br>General secondary: 2006: -0.063, 2011: -0.314 ($p<0.01$)<br>Vocational training: 2006: -0.149 ($p<0.05$), 2011: -0.429 ($p<0.01$)<br>University: 2006 -0.150 ($p<0.05$), 2011: -0.382 ($p<0.01$)<br>Unemployed (base: employed)<br>2006: 0.077, 2011: 0.083 ($p<0.1$)<br>Social class of breadwinner (base: non-skilled workers)	

						Public administration directors and directors of companies with 10 or more workers and professionals associated with a graduate degree 2006: 0.112, 2011: 0.157 ($p<0.05$)<br>Executives of public administration and of companies with fewer than 10 workers, professionals associated with university degrees<br>2006: 0.027, 2011: 0.107<br>Administrative support and professionals 2006: 0.051, 2011: 0.135 ($p<0.05$)<br>Skilled manual workers<br>2006: -0.019, 2011: 0.033<br>Semi-skilled manual workers<br>2006: -0.001, 2011: -0.001	
Duquenne, 2014	National: Greece<br>Local: Thessaly	Stratified random sample of Greek households. N=932	Exploratory factor analysis and hierarchical cluster analysis.<br>1 corresponds to no change nor reduction in the consumption, 2: limited reduction (less than 10 per cent), 3: relatively important reduction (more than 10 per cent), 4: change of food brand (less expensive) and 5: abandonment of consumption or abandonment of supply through the	Exposure: change over time<br>Time point: not stated	Questionnaire which asked about impact of Great Recession on diet.<br>Change for pasta, potatoes, olive oil, rice, bread, vegetables, milk, fruits, beef, sheep and goat, pork, cold cuts, chicken, fish, sweets, cheese, feta.	**Pasta:** 0.777 (component 1)<br>Type of consumption behaviours: no change 43%, limited reduction 10%, high reduction 7%, change of brand 37%, abandonment 2%<br>**Potatoes:** 0.775 (component 1)<br>Type of consumption behaviours: no change 47%, limited reduction 11%, high reduction 9%, change of brand 28%, abandonment 4%<br>**Olive oil:** 0.750 (component 1)<br>Type of consumption behaviours: no change 43%, limited reduction 15%, high reduction 10%, change of brand 28%, abandonment 4%<br>**Rice**: 0.747 (component 1)<br>Type of consumption behaviours: no change 40%, limited reduction 11%,	Selection total: 2<br>Comparability total: 2<br>Outcomes total: 2<br>**Total score: 6**

			market, following alternative supply modes (own production, especially in case of vegetables). Reporting pattern matrix of factor analysis and % of behaviour change. Component 1: relatively limited impact of crisis on consumers behaviour. Component 2: component 2, more impacted by crisis, >60% of houses have changed their behaviour & abandonment is quite significant.			high reduction 8%, change of brand 39%, abandonment 3%<br>**Bread:** 0.707 (component 1)<br>Type of consumption behaviours: no change 52%, limited reduction 13%, high reduction 11%, change of brand 19%, abandonment 5%<br>**Vegetables:** 0.638 (component 1)<br>Type of consumption behaviours: no change 47%, limited reduction 14%, high reduction 12%, change of brand 22%, abandonment 5%<br>**Milk:** 0.591 (component 1)<br>Type of consumption behaviours: no change 51%, limited reduction 11%, high reduction 6%, change of brand 28%, abandonment 5%<br>**Fruits:** 0.590 (component 1)<br>Type of consumption behaviours: no change 42%, limited reduction 15%, high reduction 15%, change of brand 23%, abandonment 5%<br>**Beef:** 0.664 (component 2)<br>Type of consumption behaviours: no change 35%, limited reduction 14%, high reduction 18%, change of brand 18%, abandonment 16%<br>**Sheep and goat:** 0.618 (component 2)<br>Type of consumption behaviours: no change 26%, limited reduction 9%, high reduction 17%, change of brand 15%, abandonment 33%<br>**Pork:** 0.615 (component 2)<br>Type of consumption behaviours: no change 37%, limited reduction 15%,	

						high reduction 18%, change of brand 19%, abandonment 11%<br>**Cold cuts:** 0.607 (component 2)<br>Type of consumption behaviours: no change 28%, limited reduction 8%, high reduction 17%, change of brand 26%, abandonment 22%<br>**Chicken:** 0.594 (component 2)<br>Type of consumption behaviours: no change 47%, limited reduction 16%, high reduction 11%, change of brand 21%, abandonment 5%<br>**Fish:** 0.563 (component 2)<br>Type of consumption behaviours: no change 36%, limited reduction 12%, high reduction 20%, change of brand 20%, abandonment 12%<br>**Sweets:** 0.547 (component 2)<br>Type of consumption behaviours: no change 26%, limited reduction 9%, high reduction 21%, change of brand 21%, abandonment 24%<br>**Cheese**: 0.545 (component 2)<br>Type of consumption behaviours: no change 28%, limited reduction 8%, high reduction 15%, change of brand 27%, abandonment 22%<br>**Feta:** 0.508 (component 2)<br>Type of consumption behaviours: no change 38%, limited reduction 13%, high reduction 11%, change of brand 30%, abandonment 8%	
Filippidis et al, 2014	National: Greece<br>Local: n/a	Hellas Health surveys.<br>Adults aged between 18 –	Serial Cross-sectional.<br>Outcome-specific trends and	Exposure: Commence-ment of GR (time)	5 or more fruit and vegetables per day (self-reported)	Proportion of Greek adults who reported consumption of at least five portions of fruit and vegetables per	Selection total: 2<br>Comparability total: 2

		69. Random sampling. Percentage male and N:<br>2006: n =1005, male: 48.1%,<br>2008: n=1490, male: 47.6%<br>2011: n=1008, male: 48.0%	differences between surveys were assessed by using linear coefficients in a binary logistic regression model. Polynomials were developed to account for variations in time between survey years. Results were adjusted for gender and age.	Time point 1: 2006<br>Time point 2: 2008<br>Time point 3: 2011		day, by demographic and socio-economic variables (%, 95% CI)<br>**Total**<br>2006: 21.2 (18.6–23.7);<br>2008: 9.1 (7.6–10.6);<br>2011: 7.1 (5.6–8.7).<br>2006-08 % change: −57.01% ($p<0.001$),<br>2008-2011 % change: −21.54%<br>2006-11: −66.27% ($p<0.001$)<br>**Gender**<br>Male:<br>2006: 23.2 (19.4–27.0);<br>2008: 8.4 (19.4–27.0);<br>2011: 5.6 (3.5–7.6).<br>2006-08 % change: −63.86% ($p<0.001$),<br>2008-2011 % change: −33.33%,<br>2006-11 % change: −75.91% ($p<0.001$)<br>Female:<br>2006: 19.3 (15.9–22.7;<br>2008: 9.8 (7.7–11.9);<br>2011: 8.6 (6.2–11.0).<br>2006-08 % change: −49.48% ($p<0.001$),<br>2008-2011 % change: −12.08%,<br>2006-11 % change: −55.58% ($p<0.001$)<br>**Socio-economic status**<br>Higher:<br>2006: 27.0 (20.2–33.7);<br>2008: 8.0 (5.2–10.7)<br>2011: 8.4 (4.7–12.1).<br>2006-08 % change: −70.43% ($p<0.001$),<br>2008-2011 % change: +5.23%,<br>2006-11 % change: −68.79% ($p<0.001$)<br>Middle:<br>2006: 18.1 (14.5–21.6);<br>2008: 9.8 (7.5–12.1);<br>2011: 8.9 (6.2–11.5).	Outcomes total: 2<br>**Total score: 6**

						2006-08 % change: −45.68% (p<0.001),<br>2008-2011 % change: −9.58%,<br>2006-11 % change: −50.89% (p<0.001)<br>Lower:<br>2006: 22.4 (18.1–26.6);<br>2008: 9.0 (6.5–11.6);<br>2011: 4.1 (2.0–6.2).<br>2006-08 % change: −59.63% (p<0.001),<br>2008-2011 % change: −54.82% (p<0.01),<br>2006-11 % change: −81.76% (p<0.001)	
Filippidis et al, 2017	National: Greece<br>Local: n/a	Hellas Health surveys. Adults (over 18). Percentage male and N:<br>2006: n= 1005, male: 48.1%,<br>2008: n=1490, male: 47.6%<br>2010: n= 1000, male: 50.6%<br>2011: n=1008, male: 48.0%<br>2015: n=1001, male: 48.0%	Serial Cross-sectional. Interrupted time series analysis (2010 as "intervention" year. Risk Ratio (RR) adjusted for age, gender, area of residence, education, occupation and SES.	Exposure: Commence-ment of GR (time)<br>Time point 1: 2006<br>Time point 2: 2008<br>Time point 3: 2010<br>Time point 4: 2011<br>Time point 5: 2015	Low fruit and vegetable consumption (two or less servings a day)	2008: 52.1% (49.6 to 54.7)<br>2010: n/a<br>2011: 51.3% (48.2 to 54.4)<br>2015: 51.2% (47.9 to 54.6)<br>RR (2015 vs pre-crisis): 1.00 (0.92 to 1.09)	Selection total: 2<br>Comparability total: 2<br>Outcomes total: 2<br>**Total score: 6**
Florkowski, 2012	National: Poland<br>Local: n/a	The study is based on data obtained from annual Glowny Urzad Statystyczny surveys of Polish households.	Serial cross-sectional. Households average yearly expenditure.	Exposure: Commence-ment of GR (time)<br>Time point 1: 2004<br>Time point 2: 2005<br>Time point 3: 2006	Average expenditure on bread, pasta and flour, offal and offal products, barley, pork, chicken, seafood, freshwater fish, milk, farmers cheese, hard	Bread:<br>2006:<br>All households, n= 6794, average expenditure: 47.60<br>Households above minimum income, n= 3900, average expenditure: 51.59<br>Households at or below minimum income, n= 2894, average expenditure: 42.23<br>2007:	Selection total: 2<br>Comparability total: 0<br>Outcomes total: 1<br>**Total score: 3**

				Time point 4: 2007<br>Time point 5: 2008	cheese, eggs, margarine, vegetable oil, animal fat, citrus, apples and potatoes (in zloty).	All households, n= 5897, average expenditure: 53.66<br>Households above minimum income, n= 3484, average expenditure: 58.14<br>Households at or below minimum income, n= 2413, average expenditure: 47.20<br>2008:<br>All households, n= 5789, average expenditure: 58.48<br>Households above minimum income, n= 3505, average expenditure: 63.65<br>Households at or below minimum income, n= 2284, average expenditure: 50.56<br>**Pasta and flour:**<br>2006:<br>All households, n= 12466 average expenditure: 12.73<br>Households above minimum income, n= 6299, average expenditure: 12.93<br>Households at or below minimum income, n=6167, average expenditure: 12.52<br>2007:<br>All households, n= 11036, average expenditure: 14.23<br>Households above minimum income, n= 5873, average expenditure: 14.68<br>Households at or below minimum income, n= 5163, average expenditure: 13.73<br>2008:<br>All households, n= 967, average expenditure: 16.79	

						Households above minimum income, n= 720, average expenditure: 17.25<br>Households at or below minimum income, n= 247, average expenditure: 15.47<br>**Offal and offal products:**<br>2004 (not available for 2005-2007):<br>All households, n= 3377, average expenditure: 78.35<br>Households above minimum income, n= 1822, average expenditure: 84.64<br>Households at or below minimum income, n=1555, average expenditure: 70.98<br>2008:<br>All households, n= 1579, average expenditure: 82.83<br>Households above minimum income, n= 935, average expenditure: 88.93<br>Households at or below minimum income, n= 644, average expenditure: 73.98<br>**Barley:**<br>2006:<br>All households, n= 8065, average expenditure: 3.37<br>Households above minimum income, n= 3754, average expenditure: 3.27<br>Households at or below minimum income, n= 4311, average expenditure: 3.46<br>2007:<br>All households, n= 6971, average expenditure: 3.82<br>Households above minimum income, n= 3404, average expenditure: 3.82	

						Households at or below minimum income, n= 2567, average expenditure: 3.83<br>**Pork:**<br>2006:<br>All households, n= 17754, average expenditure: 47.00<br>Households above minimum income, n= 9171, average expenditure: 49.79<br>Households at or below minimum income, n= 8583, average expenditure: 44.03<br>2007:<br>All households, n= 15996, average expenditure: 51.40<br>Households above minimum income, n= 8645, average expenditure: 54.76<br>Households at or below minimum income, n= 7351, average expenditure: 47.45<br>2008:<br>All households, n= 16100, average expenditure: 53.01<br>Households above minimum income, n= 9079, average expenditure: 56.28<br>Households at or below minimum income, n=8021, average expenditure: 48.78<br>**Chicken:**<br>2006:<br>All households, n= 18799, average expenditure: 26.52<br>Households above minimum income, n= 9604, average expenditure: 28.52	

						Households at or below minimum income, n= 9195, average expenditure: 24.43<br>2007:<br>All households, n= 16754, average expenditure: 30.93<br>Households above minimum income, n= 8956, average expenditure: 33.78<br>Households at or below minimum income, n= 7798, average expenditure: 27.65<br>2008:<br>All households, n= 16866, average expenditure: 31.61<br>Households above minimum income, n= 7467, average expenditure: 34.29<br>Households at or below minimum income, n= 7399 average expenditure: 28.19<br>**Seafood:**<br>2006:<br>All households, n= 9202, average expenditure: 16.32<br>Households above minimum income, n= 4909, average expenditure: 17.29<br>Households at or below minimum income, n= 4293, average expenditure: 15.21<br>2007:<br>All households, n= 8285, average expenditure: 17.16<br>Households above minimum income, n= 4522, average expenditure: 18.16<br>Households at or below minimum income, n= 3763 average expenditure: 15.96	

						2008:<br>All households, n= 8156, average expenditure: 17.31<br>Households above minimum income, n= 4628, average expenditure: 18.14<br>Households at or below minimum income, n= 3528, average expenditure: 16.22<br>**Freshwater fish:**<br>2006:<br>All households, n= 1633, average expenditure: 25.21<br>Households above minimum income, n= 860, average expenditure: 26.43<br>Households at or below minimum income, n= 773, average expenditure: 23.84<br>2007:<br>All households, n= 1932, average expenditure: 24.90<br>Households above minimum income, n= 1107, average expenditure: 25.96<br>Households at or below minimum income, n= 825, average expenditure: 23.48<br>2008:<br>All households, n= 2475, average expenditure: 23.27<br>Households above minimum income, n= 1431, average expenditure: 24.15<br>Households at or below minimum income, n= 1044, average expenditure: 22.06<br>**Milk:**<br>2006:	

						All households, n= 13541, average expenditure: 18.65<br>Households above minimum income, n= 6967, average expenditure: 18.00<br>Households at or below minimum income, n= 6574, average expenditure: 19.33<br>2007:<br>All households, n= 12317, average expenditure: 19.68<br>Households above minimum income, n= 6631, average expenditure: 19.32<br>Households at or below minimum income, n= 5686, average expenditure: 20.10<br>2008:<br>All households, n= 12330, average expenditure: 21.10<br>Households above minimum income, n= 6901, average expenditure: 21.09<br>Households at or below minimum income, n= 5429, average expenditure: 21.10<br>**Farmers cheese:**<br>2006:<br>All households, n= 19227, average expenditure: 17.13<br>Households above minimum income, n= 9943, average expenditure: 18.63<br>Households at or below minimum income, n= 9284, average expenditure: 15.53<br>2007:<br>All households, n= 16961, average expenditure: 17.55	

						Households above minimum income, n= 9176, average expenditure: 18.93<br>Households at or below minimum income, n= 7785, average expenditure: 15.93<br>2008:<br>All households, n= 16910, average expenditure: 18.87<br>Households above minimum income, n= 9486, average expenditure: 20.22<br>Households at or below minimum income, n= 7424, average expenditure: 17.14<br>**Hard cheese:**<br>2006:<br>All households, n= 19053, average expenditure: 18.67<br>Households above minimum income, n= 10264, average expenditure: 22.00<br>Households at or below minimum income, n= 8789, average expenditure: 14.79<br>2007:<br>All households, n= 16867, average expenditure: 20.15<br>Households above minimum income, n= 9459, average expenditure: 23.81<br>Households at or below minimum income, n= 7408, average expenditure: 15.48<br>2008:<br>All households, n= 16965, average expenditure: 21.40<br>Households above minimum income, n= 9876, average expenditure: 24.91	

						Households at or below minimum income, n= 7089, average expenditure: 16.51<br>**Eggs:**<br>2006:<br>All households, n= 19566, average expenditure: 15.42<br>Households above minimum income, n= 9868, average expenditure: 16.16<br>Households at or below minimum income, n= 9698, average expenditure: 14.67<br>2007:<br>All households, n= 17236 average expenditure:16.32<br>Households above minimum income, n=9070, average expenditure: 17.15<br>Households at or below minimum income, n= 8166, average expenditure: 15.41<br>2008:<br>All households, n= 17042, average expenditure: 16.76<br>Households above minimum income, n= 9396, average expenditure: 17.73<br>Households at or below minimum income, n= 7646, average expenditure: 15.57<br>**Margarine:**<br>2006:<br>All households, n= 17602, average expenditure: 10.19<br>Households above minimum income, n= 8972, average expenditure: 10.89	

						Households at or below minimum income, n= 8630, average expenditure: 9.46<br>2007:<br>All households, n= 15713, average expenditure: 10.60<br>Households above minimum income, n= 8423, average expenditure: 11.44<br>Households at or below minimum income, n= 7290, average expenditure: 9.63<br>2008:<br>All households, n= 15463, average expenditure: 11.95<br>Households above minimum income, n= 8721, average expenditure:12.91<br>Households at or below minimum income, n=6742, average expenditure: 10.71<br>**Vegetable oil:**<br>2006:<br>All households, n= 14636, average expenditure: 8.16<br>Households above minimum income, n= 7449, average expenditure: 8.39<br>Households at or below minimum income, n= 7187, average expenditure: 7.92<br>2007:<br>All households, n= 13073, average expenditure: 8.86<br>Households above minimum income, n= 7041, average expenditure: 9.22<br>Households at or below minimum income, n= 6032, average expenditure: 8.43	

						2008:<br>All households, n= 13036, average expenditure: 10.69<br>Households above minimum income, n= 7300, average expenditure: 11.14<br>Households at or below minimum income, n= 5736, average expenditure: 10.11<br>Animal fat:<br>2006:<br>All households, n= 8494, average expenditure: 7.37<br>Households above minimum income, n= 3910, average expenditure: 7.34<br>Households at or below minimum income, n= 4584, average expenditure: 7.40<br>2007:<br>All households, n= 7440, average expenditure: 7.65<br>Households above minimum income, n= 3595, average expenditure: 7.64<br>Households at or below minimum income, n= 3845, average expenditure: 7.66<br>2008:<br>All households, n= 6920, average expenditure: 7.86<br>Households above minimum income, n= 3502, average expenditure: 7.85<br>Households at or below minimum income, n= 3418, average expenditure: 7.86<br>**Citrus:**<br>2006:	

						All households, n= 14591, average expenditure: 9.38<br>Households above minimum income, n= 7644, average expenditure: 10.18<br>Households at or below minimum income, n= 6947, average expenditure: 8.50<br>2007:<br>All households, n= 13293, average expenditure: 10.70<br>Households above minimum income, n=7206, average expenditure: 11.63<br>Households at or below minimum income, n= 6087, average expenditure: 9.60<br>2008:<br>All households, n= 13106, average expenditure: 11.32<br>Households above minimum income, n= 7388, average expenditure: 12.26<br>Households at or below minimum income, n= 5718, average expenditure: 10.11<br>**Apples:**<br>2006:<br>All households, n= 16751, average expenditure: 10.80<br>Households above minimum income, n= 8605, average expenditure: 11.40<br>Households at or below minimum income, n= 8146, average expenditure: 10.17<br>2007:<br>All households, n=14439, average expenditure: 12.48	

						Households above minimum income, n= 7710, average expenditure: 13.02<br>Households at or below minimum income, n= 6729, average expenditure: 11.85<br>2008:<br>All households, n= 14120, average expenditure: 12.37<br>Households above minimum income, n= 7797, average expenditure: 12.79<br>Households at or below minimum income, n= 6323, average expenditure: 11.86<br>**Potatoes:**<br>2006:<br>All households, n= 17347, average expenditure: 21.31<br>Households above minimum income, n= 8632, average expenditure: 21.96<br>Households at or below minimum income, n= 8715, average expenditure: 20.67<br>2007:<br>All households, n= 15302, average expenditure: 23.01<br>Households above minimum income, n= 7975, average expenditure: 23.98<br>Households at or below minimum income, n= 7327, average expenditure: 21.97<br>2008:<br>All households, n= 15035, average expenditure: 18.02<br>Households above minimum income, n= 8192, average expenditure: 18.88	

						Households at or below minimum income, n= 6843, average expenditure: 17.00	
García-Mayor	National: Spain<br>Local: n/a	Spanish National Health Survey.<br>Adults aged 18-64<br>N=72,574	Serial Cross-sectional.<br>Descriptive analysis estimating frequencies.<br>Multivariate logistic regression adjusting for age, gender and social class.	Exposure: Commence-ment of GR (time)<br>Time point 1: 2006,<br>Time point 2: 2012<br>Time point 3: 2017	Daily fruit, vegetable, pastries and sweets, sweetened beverage, intake (Y/N)	**Fruit:**<br>Proportion consuming fruit daily:<br>2006: Men 59.7% (95% CI 58.3–61.0), women: 71.4% (95% CI 70.4–72.3)<br>2012: Men 56.3% (95% CI 54.8–57.9), women: 64.2% (95% C 62.9–65.6)<br>2017: Men:55.1% (95% CI 53.6–56.7), women: 66.5% (95% CI 65.2–67.7)<br>Multinomial logistic regression stratified by social class by examining health indicators in men<br>Daily fruit consumption:<br>High social class:<br>2012: 0.90 (0.78–1.05)<br>2017: 0.84 (0.72–0.97) $p < 0.05$.<br>Middle social class:<br>2012: 0.87 (0.75–1.00)<br>2017: 0.81 (0.70–0.93) $p < 0.01$.<br>Low social class:<br>2012: 0.83 (0.76–0.90) $p < 0.001$.<br>2017: 0.72 (0.66–0.79) $p < 0.001$.<br>In women:<br>Daily fruit consumption:<br>High social class:<br>2012: 0.70 (0.61–0.81) $p < 0.001$.<br>2017: 0.98 (0.85–1.14)<br>Middle social class:<br>2012: 0.68 (0.59–0.78) $p < 0.001$.<br>2017: 0.70 (0.61–0.80) $p < 0.001$.<br>Low social class:<br>2012: 0.72 (0.67–0.79) $p < 0.001$.<br>2017: 0.69 (0.63–0.75) $p < 0.001$.<br>**Vegetables**	Selection total: 3<br>Comparability total: 2<br>Outcomes total: 2<br>**Total score: 7**

						Proportion consuming vegetables daily:<br>2006: Men 35.0% (95% CI 33.3–36.8), women: 48.2% (95% CI 47.0–49.5)<br>2012: Men 39.8% (95% CI 38.0–41.6, women: 51.7% (95% CI 50.2–53.3)<br>2017: Men: 32.9% (95% CI 31.1–34.8), women: 47.5% (95% CI 45.9–49.1)<br>Multinomial logistic regression stratified by social class by examining health indicators in men<br>Daily veg consumption:<br>High social class:<br>2012: 1.37 (1.19–1.58) $p < 0.001$.<br>2017: 0.99 (0.85–1.14)<br>Middle social class:<br>2012: 1.30 (1.13–1.50) $p < 0.001$.<br>2017: 0.87 (0.75–1.00)<br>Low social class:<br>2012: 1.14 (1.05–1.25) $p < 0.01$.<br>2017: 0.82 (0.75–0.90) $p < 0.001$.<br>In women:<br>Daily veg consumption:<br>High social class:<br>2012: 1.19 (1.05–1.36) $p < 0.01$.<br>2017: 1.10 (0.97–1.25)<br>Middle social class:<br>2012: 1.26 (1.11–1.42) $p < 0.001$.<br>2017: 0.92 (0.82–1.04)<br>Low social class:<br>2012: 1.14 (1.06–1.24) $p < 0.01$.<br>2017: 0.92 (0.85–0.99) $p < 0.05$<br>**Pastries and sweets**<br>Proportion consuming pastries and sweets daily	

						2006: Men 34.0% (95% CI 32.2–35.7), women: 35.9% (95% CI 34.4–37.3)<br>2012: Men 29.6% (95% CI 27.6–31.5), women: 29.2% (95% CI 27.3–31.1)<br>2017: Men: 25.2% (95% CI 23.2–27.2), women: 26.6% (95% CI 24.8–28.5)<br>Multinomial logistic regression stratified by social class by examining health indicators in men<br>Daily consumption:<br>High social class:<br>2012: 0.87 (0.75–1.01)<br>2017: 0.64 (0.54–0.75) p < 0.001<br>Middle social class:<br>2012: 0.71 (0.61–0.83) p < 0.001<br>2017: 0.64 (0.54–0.74) p < 0.001<br>Low social class:<br>2012: 0.64 (0.54–0.74) p < 0.001.<br>2017: 0.68 (0.62–0.75) p < 0.001<br>In women:<br>Daily consumption:<br>High social class:<br>2012: 0.79 (0.69–0.91) p < 0.01<br>2017: 00.61 (0.53–0.70) p < 0.001<br>Middle social class:<br>2012: 0.71 (0.62–0.81) p < 0.001<br>2017: 0.67 (0.58–0.76) p < 0.001<br>Low social class:<br>2012: 0.75 (0.69–0.82) p < 0.001<br>2017: 0.68 (0.62–0.74) p < 0.001<br>**Sweetened drinks**<br>Proportion consuming sweetened drinks daily<br>2006: Men 20.2% (95% CI 18.3–22.2), women: 13.6% (95% CI 12.0–15.3)	

						2012: Men 15.5% (95% CI 13.4–17.7), women: 10.1% (95% CI 7.9–12.2)<br>2017: Men: 11.3% (95% CI 9.1–13.4), women: 8.6% (95% CI 6.5–10.7)<br>Multinomial logistic regression stratified by social class by examining health indicators in men<br>Daily consumption:<br>High social class:<br>2012: 0.43 (0.33–0.56) $p < 0.001$.<br>2017: 0.75 (0.60–0.93) $p < 0.001$.<br>Middle social class:<br>2012: 0.69 (0.56–0.85) $p < 0.001$.<br>2017: 0.59 (0.47–0.73) $p < 0.001$.<br>Low social class:<br>2012: 0.73 (0.66–0.82) $p < 0.001$.<br>2017: 0.56 (0.50–0.63) $p < 0.001$.<br>In women:<br>Daily consumption:<br>High social class:<br>2012: 0.74 (0.58–0.95) $p < 0.05$.<br>2017: 0.47 (0.36–0.63) $p < 0.001$.<br>Middle social class:<br>2012: 0.73 (0.59–0.90) $p < 0.01$.<br>2017: 0.65 (0.52–0.81) $p < 0.001$.<br>Low social class:<br>2012: 0.67 (0.60–0.75) $p < 0.001$.<br>2017: 0.65 (0.58–0.73) $p < 0.001$.	
Griffith et al, 2016a	National: UK<br>Local: n/a	Kantar Worldpanel data for UK households. n=14,694.	Longitudinal study. HEI scores and percentage change.	Exposure: Commence-ment of GR (time)<br>Time point 1: 2005-2007,<br>Time point 2: 2010-12	Healthy Eating (HEI) score for fruit, vegetables, grains, milk, meat, oils.<br>Share of calories from fruit, vegetables,	**Healthy Eating (HEI) score**<br>Total fruit:<br>Max score: 5<br>Mean in 2005-7: 3.06<br>Change to 2010-12: - 0.02<br>Percentage change to 2010-12: - 0.7<br>Whole fruit:<br>Max score: 5	Selection total: 3<br>Comparability total: 2<br>Outcomes total: 2<br>**Total score: 7**

					grains, dairy, cheese and fats, poultry and fish, red meat and nuts, drinks, prepared sweet and prepared savoury. Outcome data of food purchases from all types of stores using an electronic hand-held scanner in the home.	Mean in 2005-7: 3.36<br>Change to 2010-12: 0.08<br>Percentage change to 2010-12: 2.4<br>Total vegetables:<br>Max score: 5<br>Mean in 2005-7: 3.20<br>Change to 2010-12: - 0.13<br>Percentage change to 2010-12: - 4.1<br>Dark green/orange vegetables:<br>Max score: 5<br>Mean in 2005-7: 1.61<br>Change to 2010-12: 0.00<br>Percentage change to 2010-12: 0.00<br>Total grains:<br>Max score: 5<br>Mean in 2005-7: 3.69<br>Change to 2010-12: - 0.03<br>Percentage change to 2010-12: - 0.8<br>Whole grains:<br>Max score: 5<br>Mean in 2005-7: 1.55<br>Change to 2010-12: - 0.11<br>Percentage change to 2010-12: - 7.1<br>Milk<br>Max score: 10<br>Mean in 2005-7: 5.28<br>Change to 2010-12: -0.05<br>Percentage change to 2010-12: -0.9<br>Meat<br>Max score: 10<br>Mean in 2005-7: 7.96<br>Change to 2010-12: -0.22<br>Percentage change to 2010-12: - 2.8<br>Oils<br>Max score: 10<br>Mean in 2005-7: 4.93	

						Change to 2010-12: - 0.18<br>Percentage change to 2010-12: -3.7<br>**Share of calories**<br>Fruit<br>2005-7: 5.08<br>2010-12: 5.28<br>Change: 0.20, Percentage change: 3.86<br>Vegetables<br>2005-7: 6.97<br>2010-12: 6.43<br>Change: - 0.54, Percentage change: - 7.81<br>Grains<br>2005-7: 16.40<br>2010-12: 16.65<br>Change: 0.24, Percentage change: 1.48<br>Dairy<br>2005-7: 9.53<br>2010-12: 9.49<br>Change: - 0.04, Percentage change: - 0.46<br>Cheese and fats<br>2005-7: 11.73<br>2010-12: 11.73<br>Change: 0.01, Percentage change: 0.06<br>Poultry and fish<br>2005-7: 3.09<br>2010-12: 3.30<br>Change: 0.21, Percentage change: 6.87<br>Red meat and nuts<br>2005-7: 8.34<br>2010-12: 7.84<br>Change: - 0.51, Percentage change: - 6.07<br>Drinks<br>2005-7: 1.87	

						2010-12: 1.82<br>Change: - 0.04, Percentage change: - 2.36<br>Prepared sweet<br>2005-7: 19.06<br>2010-12: 19.53<br>Change: 0.47, Percentage change: 2.47<br>Prepared savoury<br>2005-7: 14.78<br>2010-12: 14.82<br>Change: 0.04, Percentage change: 0.30	
Griffith et al, 2016b	National: UK<br>Local: n/a	National Food Survey, Family Expenditure Survey/ Living Costs and Food Survey.<br>Households.<br>N not stated.	Serial cross-sectional<br>Change in calories and expenditure.<br>Calorie density calculated using backcasting:<br>Backcast 1 captures the cross-household variation by including the household characteristics (employment status, age, household structure, interactions between time effects and age and time effects and employment status) and assumes that variation is entirely driven by changes in these characteristics.<br>Backcast 2 includes seasonal variation and changes in the	Exposure: Commence-ment of GR (time)<br>Time point 1: 1980 – 2007<br>Time point 2: 2007 - 2013	Calories percentage change and expenditure percentage change over two time periods on eating out and fast food (food eaten at restaurants, cafes, bars, bistros, fast food outlets and takeaways), confectionary and soft drinks (in and outside the home). Also for foods eaten inside the home: grains, meat, cooking oil and fats, fruits and vegetables, dairy	**Eating out and fast food:** Equivalised daily expenditure:<br>1980: 1.17, 2007: 1.61, 2013: 1.53,<br>Percentage change: 1980-2007: 37.5<br>Percentage change 2007-13: -4.9<br>Backcast calorie density 1: demographic variation in calorie density:<br>1980: 220, 2007: 279, 2013: 256,<br>Percentage change: 1980-2007: 27.1,<br>Percentage change 2007-13: −8.3<br>Backcast calorie density 2: demographic variation & time variation in calorie density:<br>1980: 267, 2007: 268, 2013: 255,<br>Percentage change: 1980-2007: 0.6<br>Percentage change 2007-13: −1.0%<br>Calorie shares: 1980: 7.9, 2007: 12.4, 2013: 11.7,<br>Percentage change: 1980-2013: +3.8<br>**Confectionary and soft drinks:**<br>Equivalised daily expenditure:<br>1980: 0.26, 2007: 0.37, 2013: 0.33,<br>Percentage change: 1980-2007: 39.4,<br>Percentage change 2007-13: −10.4%	Selection total: 3<br>Comparability total: 2<br>Outcomes total: 1<br>**Total score: 6**

			prices of food at home and food group, and includes a second-order polynomial in log total expenditures for all food and drink, as well as for food at home		products, sugary products and preserves (e.g. jams etc.), fruit and vegetables, cheese, eggs, other (including ready meals) and fish. Expenditure is equivalized using the OECD Oxford scale. Calories are reported per person per day, they are individually allocated using daily recommended calorie intake by age and gender of the household members.	Backcast calorie density 1: demographic variation in calorie density:<br>1980: 88, 2007: 117, 2013: 106,<br>Percentage change: 1980-2007: 32.9<br>Percentage change 2007-13: −9.3<br>Backcast calorie density 2: demographic variation & time variation in calorie density:<br>1980: 78, 2007: 113, 2013: 118,<br>Percentage change: 1980-2007: 44.2,<br>Percentage change 2007-13: 5.1<br>Calorie shares: 1980: 3.2, 2007: 5.2, 2013: 4.9,<br>Percentage change: 1980-2013: +1.7<br>**Grains (at home):**<br>Calories:<br>1980: 845,<br>Percentage change: 1980-2007: −14.8,<br>Percentage change: 2007-13: −2.6<br>Expenditure shares,<br>1980: 0.18,<br>Percentage change: 1980-2007: −8.9,<br>Percentage change: 2007-13: 4.9%<br>**Meat (at home):**<br>Calories,<br>1980: 413,<br>Percentage change: 1980-2007: −28.9,<br>Percentage change: 2007-13: −9.0<br>Expenditure shares,<br>1980: 0.28,<br>Percentage change: 1980-2007: −17.3,<br>Percentage change: 2007-13: −0.5<br>**Fats and oils (at home):**<br>Calories,<br>1980: 397,	

						Percentage change: 1980-2007: −51.1,<br>Percentage change: 2007-13: −0.7<br>Expenditure shares,<br>1980: 0.04,<br>Percentage change: 1980-2007: −47.4,<br>Percentage change: 2007-13: 10.5.<br>**Fruit & Vegetables (at home):**<br>Calories,<br>1980: 301,<br>Percentage change: 1980-2007: 5.7,<br>Percentage change: 2007-13: −2.9<br>Expenditure shares,<br>1980: 0.18,<br>Percentage change: 1980-2007: 44.9,<br>Percentage change: 2007-13: −3.3<br>**Dairy (at home):**<br>Calories,<br>1980: 285,<br>Percentage change: 1980-2007: −32.7,<br>Percentage change: 2007-13: −5.9<br>Expenditure shares,<br>1980: 0.13,<br>Percentage change: 1980-2007: −22.1,<br>Percentage change: 2007-13: −7.5<br>**Sugary products and preserves (at home):**<br>Calories,<br>1980: 278,<br>Percentage change: 1980-2007: −73.7,<br>Percentage change: 2007-13: −1.8<br>Expenditure shares,<br>1980: 0.03,<br>Percentage change: 1980-2007: −62.1,<br>Percentage change: 2007-13: 6.7<br>**Cheese (at home):**<br>Calories,	

						1980: 68,<br>Percentage change: 1980-2007: 0.0,<br>Percentage change: 2007-13: −0.3<br>Expenditure shares,<br>1980: 0.03,<br>Percentage change: 1980-2007: 13.8,<br>Percentage change: 2007-13: 4.4<br>**Eggs (at home):**<br>Calories,<br>1980: 40,<br>Percentage change: 1980-2007: −50.6,<br>Percentage change: 2007-13: −4.3<br>Expenditure shares,<br>1980: 0.02,<br>Percentage change: 1980-2007: −46.1,<br>Percentage change: 2007-13: 14.5<br>**Other (at home, including ready meals):**<br>Calories,<br>1980: 27,<br>Percentage change: 1980-2007:114.2,<br>Percentage change: 2007-13: 7.2<br>Expenditure shares,<br>1980: 0.03,<br>Percentage change: 1980-2007: 92.2,<br>Percentage change: 2007-13: 2.5<br>**Fish (at home):**<br>Calories,<br>1980: 23,<br>Percentage change: 1980-2007: 43.0,<br>Percentage change: 2007-13: −3.5<br>Expenditure shares,<br>1980: 0.03,<br>Percentage change: 1980-2007: 37.9,<br>Percentage change: 2007-13: −2.6	

Griffith et al, 2013	National: UK<br>Local: n/a	Kantar Worldpanel data.<br>N=15,850.	Longitudinal.<br>Regressed variables on three time-period dummies and controlled for month and household fixed effects, estimating regression separately by household.	Exposure: Commence-ment of GR (time)<br>Time point 1: 2005-2007<br>Time point 2: 2008-2009<br>Time point 3:	Change in share of calories from fruit and vegetables (g per 100g).<br>Participants record spending on all grocery purchases via an electronic hand-held scanner in the home (purchases brought into the home).	Single non-pensioners:<br>2008-09: – 1.05, 2010-12: – 1.08,<br>Single pensioners:<br>2008-09: – 1.16, 2010-12: – 1.11<br>Couple non-pensioners:<br>2008-09: – 0.93, 2010-12: – 0.90<br>Couple pensioners:<br>2008-09: – 0.82, 2010-12: – 1.10<br>Multi-adult households:<br>2008-09: – 0.51, 2010-12: – 0.64<br>Single parents:<br>2008-09: – 0.82, 2010-12: – 1.22<br>2+ adults, young children:<br>2008-09: – 0.85, 2010-12: – 1.20<br>2+ adults, older children:<br>2008-09: – 0.43, 2010-12: – 0.71<br>All households:<br>2008-09: – 0.80, 2010-12: – 0.94<br>All the changes are statistically different from zero at the 99% level.	Selection total: 3<br>Comparability total: 2<br>Outcomes total: 2<br>**Total score: 7**
Hasan, 2019	National: Bangladesh<br>Local: n/a	Bangladesh Household Income and Expenditure Survey.<br>Repeated cross-sectional study using two-stage stratified random sampling.<br>Analysis was done for those who buy rice (compared to autarkic	Serial cross-sectional design.<br>The study used difference-in-difference framework and OLS models including district fixed effects and employing clustered standard errors (weighted).	Exposure: Commence-ment of GR (time)<br>Time point 1: 2005 (n=4,978), time point 2: 2010 (n=6,744).	Consumed rice (kg) and calorie intake from grain, non-rice grain, pulses (general), high value pulses (Lentil, Chickling vetch and Green Gram), low value pulses (Pea Gram, Mashkalai and other types of pulse), fruits, high value fish, low value fish and other items (all in	Consumed rice:<br>2010 coefficient: 1.14 (0.23) p<0.01<br>Calorie intake from grain<br>2010 coefficient: − 45.92 (27.92)<br>Calorie intake from non-rice grain:<br>2010 coefficient: 82.90 (11.20) p<0.01<br>Calorie intake from pulses (general),<br>2010 coefficient: 2.61 (2.92)<br>Calorie intake from high value pulses:<br>2010 coefficient: 1.05 (2.25)<br>Calorie intake from low value pulses:<br>2010 coefficient: 1.56 (1.75)<br>Calorie intake from fruits:<br>2010 coefficient: 17.35 (7.09) p<0.05<br>Calorie intake from proteins:<br>2010 coefficient: 8.31 (5.38)	Selection total: 3<br>Comparability total: 2<br>Outcomes total: 2<br>**Total score: 7**

		households and rice sellers, but there was no significant difference between these types).<br>n=11,722			kcal per capita per day).	Calorie intake from high value fish:<br>2010 coefficient: −3.10 (1.50) p<0.05<br>Calorie intake from low value fish:<br>2010 coefficient: 9.75 (1.80) p<0.01<br>Calorie intake from other items:<br>2010 coefficient: 28.50 (8.32) p<0.01	
Jofre-Bonet et al., 2016	National: UK<br>Local: England	Health Survey for England. Adults over 16, 44.78% male.<br>N=91,045	Serial cross-sectional. Non-linear estimation methods (Tobit and probit), reporting Average Marginal Effects. Impact of recession examined using the AME of the total effect of 2008 dummy variable, which involves the effect of the d08 coefficient plus its effect through the interaction with UR.	Exposure: annual unemploy-ment rate (UR) by Government Office Region. Plus, dummy variable for 2008 onwards (d08).<br>Time: 2001-2013 (excluding 2012).	Portions of fruits/vegetables eaten the day before being surveyed.	**Fruits:**<br>Association of UR and vegetable consumption: 0.0073 (0.014)<br>Interaction between UR and d08: −0.1962 (0.068) (p <0.01), suggesting that fruit consumption decreased by 0.196 portions on average.<br>**Vegetables:**<br>Association of UR and vegetable consumption: −0.0090 (0.010).<br>Interaction between UR and d08: 0.0916 (0.051) (p <0.1), suggesting that vegetable consumption increased by 0.092 portions.<br>Suggests that the recession had an impact on fruit and vegetable consumption that did not originate in change in UR.	Selection total: 3<br>Comparability total: 2<br>Outcomes total: 2<br>**Total score: 7**
Kim & Cubbin, 2019	National: USA<br>Local: California	The Geographic Research on Wellbeing (GROW) study is a follow-up survey of postpartum women 2012-2013.	Cross-sectional outcome with longitudinal exposure.<br>1) Bivariate analyses to examine food environment by individual-level and neighbourhood level	Exposure: changes in three neighbour-hood-level indicators before/after the Great Recession: (1) median	Binary variable relating to mother-reported availability of fruit or vegetables (excluding potatoes) in the home (very often = 1; otherwise = 0)	**Fruits:**<br>1) Bivariate analysis for fruit very often in the home<br>Total - poor families: 81.0%, non-poor families: 93.8%<br><u>Difference in median household income</u><br><$0 - poor families: 69.4%, non-poor families: 98.5%	Selection total: 3<br>Comparability total: 2<br>Outcomes total: 2<br>**Total score: 7**

		N=1359 households.	characteristics separately.<br>2) Logistic regression for odds ratios for dependent variables for poor families: a "sociodemographic" model (Model 1); "neighbourhood economic change" models (Models 2-4), which added variables of neighbourhood economic change one at a time to the sociodemographic model; and a fully adjusted model (Model 5), which added all three variables of neighbourhood economic change to the socio-demographic model.	household income, (2) proportions of vacant housing units, and (3) median housing value (economic changes between 2000 and 2009-2013).<br>Time: 2000-2013		\$0-\$100,000 - poor families: 80.4%, non-poor families: 95.4%<br>\$100,000-\$149,999 - poor families: 86.4%, non-poor families: 93.9%<br>\$150,000+: poor families: 85.2%, non-poor families: 90.4%<br><u>Difference in % of vacant housing units</u><br><0.00%: poor families: 77.2%, non-poor families: 92.8%<br>0.00% - 2.99%: poor families: 81.8%, non-poor families: 94.1%<br>3.00-4.99%: poor families: 86.7%, non-poor families: 92.5%<br>>5.00%: poor families: 79.8%, non-poor families: 95.9%<br><u>Difference in median housing values:</u><br><\$100,000: poor families: 77.0%, non-poor families: 90.2%<br>\$100,000 - \$149,000: poor families: 83.9%, non-poor families: 95.1%<br>\$150,000 - \$249,999: poor families: 79.0%, non-poor families: 92.6%<br>\$250,000+: poor families: 87.9%, non-poor families: 95.2%<br>2) Logistic regression<br><u>Difference in median household income</u><br><\$0 - ref, all 1.00<br>\$0-\$100,000: Model 2: 2.04 [1.07, 3.90] ($p<0.05$), Model 5: 1.92 [0.99, 3.73]<br>\$100,000-\$149,999: Model 2: 3.32 [1.52, 7.27] ($p < 0.01$), Model 5: 3.13 [1.42, 6.94] ($p < 0.01$)	

						\$150,000+: Model 2: 2.88 [1.30, 6.39] (p < 0.01), Model 5: 2.70 [1.19, 6.12] (p < 0.05)<br><u>Difference in % of vacant housing units</u><br><0.00%: Model 3: 0.96 [0.47, 1.93], Model 5: 0.99 [0.48, 2.03]<br>0.00% - 2.99%: Model 3: 1.22 [0.62, 2.39], Model 5: 1.13 [0.55, 2.31]<br>3.00-4.99%: Model 3: 1.69 [0.73, 3.93], Model 5: 1.73 [0.74, 4.06]<br>>5.00%: ref, all 1.00<br><u>Difference in median housing values</u><br><\$100,000: ref, all 1.00<br>\$100,000 - \$149,000: Model 4: 1.57 [0.83, 2.99], Model 5: 1.33 [0.69, 2.56]<br>\$150,000 - \$249,999: Model 4: 1.17 [0.63, 2.18], Model 5: 1.00 [0.53, 1.91]<br>\$250,000+: Model 4: 1.97 [0.81, 4.79], Model 5: 1.54 [0.61, 3.88]<br>**Vegetables**<br>Total - poor families: 78.5%, non-poor families: 91.1%<br><u>Difference in median household income</u><br><\$0: poor families: 65.5%, non-poor families: 93.6%<br>\$0-\$100,000: poor families: 78.4%, non-poor families: 89.7%<br>\$100,000-\$149,999 - poor families: 83.7%, non-poor families: 91.6%<br>\$150,000+: poor families: 83.3%, non-poor families: 90.7%<br><u>Difference in % of vacant housing units</u><br><0.00%: poor families: 76.2%, non-poor families: 91.3%	

						0.00% - 2.99%: poor families: 79.9%, non-poor families: 93.3%<br>3.00-4.99%: poor families: 83.7%, non-poor families: 88.2%<br>>5.00%: poor families: 74.6%, non-poor families: 88.5%<br>Difference in median housing values<br><$100,000: poor families: 78.1%, non-poor families: 91.3%<br>$100,000 - $149,000: poor families: 79.8%, non-poor families: 89.7%<br>$150,000 - $249,999: poor families: 76.1%, non-poor families: 90.3%<br>$250,000+: poor families: 83.5%, non-poor families: 92.3%<br>2) Logistic regression<br>Difference in median household income<br><$0 - ref, all 1.00<br>$0-$100,000: Model 2: 2.15 [1.17, 3.94] (p<0.05), Model 5: 2.14 [0.38, 1.90] (p<0.05)<br>$100,000-$149,999: Model 2: 3.23 [1.55, 6.71] (p < 0.01), Model 5: 3.31 [1.57, 6.97] (p < 0.01)<br>$150,000+: Model 2: 3.10 [1.43, 6.70] (p < 0.01), Model 5: 3.26 [1.50, 7.09] (p < 0.01)<br>Difference in % of vacant housing units<br><0.00%: Model 3: 1.17 [0.61, 2.25], Model 5: 1.23 [0.63, 2.39]<br>0.00% - 2.99%: Model 3: 1.45 [0.78, 2.70], Model 5: 1.39 [0.73, 2.62]<br>3.00-4.99%: Model 3: 1.86 [0.85, 4.04], Model 5:1.87 [0.86, 4.06]<br>> 5.00%: ref, all 1.00	

						Difference in median housing values<br><$100,000: ref, all 1.00<br>$100,000 - $149,000: Model 4: 1.12 [0.60, 2.10], Model 5: 0.92 [0.49, 1.70]<br>$150,000 - $249,999: Model 4: 0.94 [0.51, 1.74], Model 5: 0.76 [0.41, 1.40]<br>$250,000+: Model 4: 1.32 [0.55, 3.20], Model 5: 0.98 [0.39, 2.43]	
Kotel-nikova & Radaev, 2017		Russian Longitudinal Monitoring Survey (RLMS–HSE). The RLMS–HSE is a nationally representative annual panel survey of households. Multistage probability sampling.<br>Aged 14+.<br>N= between 3,317 and 6,180.	Longitudinal. Median changes in per capita food expenditures (percentage change to previous year).	Exposure: Commence-ment of GR (time)<br>Time point 1: 1995 (n=3,317)<br>Time point 2: 1998 (n=3,248)<br>Time point 3: 2009 (n=4,900)<br>Time point 4: 2014 (n=6180)	Food expenditure in the previous week on: bread, cereals, and canned food; fresh vegetables; fresh meat and fish; milk and dairy products; and berries and other fresh fruit.	Bread, cereals and canned food:<br>1995: 4.8 1998: -41.4, 2009: -5.2, 2014: -8.6 (not significant in 2009 and 2014).<br>Fresh vegetables:<br>1995: -69.3, 1998: -41.3, 2009: -43.6, 2014: -46.4<br>Fresh meat and fish:<br>1995: -29.9, 1998: -100.0, 2009: -4.8, 2014: -7.7<br>Milk and dairy products:<br>1995: -23.7, 1998: -52.7, 2009: -7.1, 2014: -5.2<br>Berries and other fresh fruits:<br>1995: -74.3, 1998: -100.0, 2009: -37.1, 2014: -23.7	Selection total: 3<br>Comparability total: 1<br>Outcomes total: 1<br>**Total score: 5**
Martin-Prevel et al, 2012	National: Burkina Faso<br>Local: Ouaga-dougou	Households randomly selected from Ouagadougou census. Average age of household head: 42, household head 86.8% (2007)	Serial cross-sectional. Changes in food-related indicators between 2007 and 2008 were analysed using chi-square tests for proportions.	Exposure: Commence-ment of GR (time)<br>Time Point 1: July 2007 (n=3017)<br>Time Point 2: July 2008 (n=3002)	Daily food expenditure. Obtain a food basket price per item per day calculating by summing mean price per kilogram and prices multiplied by	Changes in proportion of people consuming food groups in the preceding 24 hours 2007 to 2008:<br>Cereals: 0% increase<br>Roots and tubers: 34% increase<br>Vitamin A rich fruits/vegetables consumption: 31% decrease<br>Green leafy vegetables: 2% increase<br>Other vegetables: 2% decrease<br>VA-rich fruits: 69% decrease	Selection total: 3<br>Comparability total: 2<br>Outcomes total: 2<br>**Total score: 7**

		and 87.8% (2008) male.			daily consumption of that food item. Weighted prices were also summed by type of food to obtain the basket price of cereals, meat/fish, and fruit/vegetables per day.	Other fruits: 31% decrease<br>Liver/offal: 25% decrease<br>Other offals: 40% decrease<br>Meat and poultry: 18% decrease<br>Eggs: 42% increase<br>Fish: 4% decrease<br>Nuts/seeds: 15% decrease<br>Beans: 8% increase<br>Milk/dairy product: 21% decrease<br>Oils/fats: 6% decrease<br>Vitamin A rich oil (red palm oil): 221% increase<br>Condiments: 1% increase	
Mattei et al, 2017	National: Italy<br>Local: n/a	Italian Institute of Statistics database, aged over 3.<br>Age: 11 and above.	Serial Cross-sectional.<br>Linear Regression Models including a dummy variable for before/after 2008. 95% confidence intervals (CIs) were estimated for all coefficients on the basis of heteroscedasticity-robust standard errors.	Exposure: Commence-ment of GR (time)<br>Time point 1: 2000-2007<br>Time point 2: 2008-2015	Percentage of people who eat meat sometimes in the week, eat fish sometimes in the week, eat cheese at least once a day, eat vegetables at least once a day.	**Eating meat sometimes in the week:**<br>β: -1.19, p: 0.60, 95% CI: -6.12, 3.73, R2: 0.42; Time-trend association: not significant<br>**Eating fish sometimes in the week:**<br>β: -1.64, p: 0.22, 95% CI: -4.44, 1.16, R2: 0.29; Time-trend association: not significant<br>**Eating cheese at least once a day:**<br>β: 0.67, p: 0.47, 95% CI: -1.29, 2.64, R2: 0.94; Time-trend association: non-significant negative time trend.<br>**Eating vegetables at least once a day:**<br>β: -1.05, p: 0.35, 95% CI: -3.41, 1.30, R2: 0.81; Time-trend association: non-significant positive trend.	Selection total: 1<br>Comparability total: 0<br>Outcomes total: 2<br>**Total score: 3**
Ng et al., 2014	National: USA<br>Local: n/a	Neilson Homescan data. Weighted to be nationally representative.<br>N= 57,298 households	Cross-sectional and longitudinal data.<br>Maximum likelihood random effect models with clustering by household.	Exposure: unemploy-ment rate (monthly market and quarter-specific rates	Calories from Consumer Packaged Goods (CPG) and beverages.	**Households with children:**<br>Coefficient for one-percentage point increase in unemployment<br>Foods & beverages: 4.05 ± 0.85 p<0.001<br>Foods: 3.83 ± 0.75 p<0.001<br>Beverages: -0.02 ± 0.18	Selection total: 4<br>Comparability total: 2<br>Outcomes total: 2<br>**Total score: 8**

		(602, 389 observations).	Controlled for household composition, income, household head's age and education, race-ethnicity, market, seasonality, and year and market-level price and includes interactions between market and year, unemployment and year, unemployment and race-ethnicity, and race-ethnicity and year.	from Bureau of Labour Statistics' Local Area Unemployme nt Statistics), time.<br>Time:<br>2000-2011		**Households with adults only:**<br>Coefficient for one-percentage point increase in unemployment:<br>Foods & beverages: 1.64 ± 0.85<br>Foods: 1.08 ± 0.63<br>Beverages: 0.42 ± 0.16 p<0.01<br>Great Recession – proxied by unemployment – associated with small increases in caloric purchases, where one percentage-point increase in unemployment rate in the local market was associated with a 1.6-4.1-kcal/day/capita increase in total calories purchased.	
Nour et al., 2019	National: Canada<br>Local: n/a	Canadian Community Health Survey (CCHS), a nationally representative survey. 2-month data collection period.<br>Aged 15-64.<br>N=281,421	Serial cross-sectional. Logistic Regression model. Covariates included: age, gender, marital status and education, household income distribution, dwelling ownership and employment status.	Exposure: Commence-ment of GR (time) (periods determined by GDP and employment indicators).<br>Time point 1: Pre-crisis<br>Jan. 2007 – Aug. 2008<br>Time point 2: Crisis<br>Sept. 2008 – July 2009<br>Time point 3: Stimulus	Consuming 5 or more servings of fruit and vegetables per day.	Adjusted ORs (95% CI) - all estimates weighted and 95% Cis derived using bootstrap resampling methods.<br>Pre-crisis period: 1.00 (ref)<br>Crisis period: 1.03 (0.97 to 1.08)<br>Stimulus period: 0.98 (0.94 to 1.02)<br>Austerity period: 0.88 (0.85 to 0.91)<br>(P<0.001)	Selection total: 4<br>Comparability total: 1<br>Outcomes total: 2<br>**Total score: 7**

				July 2009 – Feb 2011<br>Time point 4: Austerity March 2011 – December 2013			
Rajmil et al, 2013	National: Spain<br>Local: Catalonia	Catalan Health Survey. Multistage probability sample.<br>Aged 3-15.<br>48.5% male in 2006 and 49.3% male in 2010-2012.<br>Total n=3982.	Serial cross-sectional. Multiple linear regression, adjusted for other factors in table (sex, maternal education, survey, employment).	Exposure: Commence-ment of GR (time)<br>Time point 1: 2006 (n=2220)<br>Time point 2: 2010-2012 (n=1967)	Junk food consumption assessed using 4 items from the Child Health and Illness profile. Higher scores reflect less junk food consumption	Boys: ref, girls: 0.53 (−0.12 to 1.18)<br>Age: -0.3 (−0.4 to −0.21) (statistically significant)<br>Maternal education<br>Primary maternal education: −4.64 (−6.07 to −0.21) (statistically significant)<br>Secondary maternal education: −2.25 (−3.37 to −1.13) (statistically significant)<br>Survey<br>2006: ref, 2010: -12: 0.89 (−0.27 to 2.06)<br>Employed: ref, Unemployed: 0.4 (−1.45 to 2.27)<br>Interaction terms:<br>Primary education by survey: 2.85 (0.83 to 4.88) (statistically significant)<br>Secondary education by survey: 1.22 (−0.22 to 2.67)	Selection total: 3<br>Comparability total: 2<br>Outcomes total: 2<br>**Total score: 7**
Regidor et al., 2019	National: Spain<br>Local: n/a	The data on fruit and vegetable consumption are from the survey 'Panel of Food Consumption' carried out by	Serial cross-sectional. Segmented linear regression models, where the outcomes were the natural logarithms of GDP, health behaviours indicators and mortality rates.	Macro-economic fluctuations characterised by the annual gross domestic product (GDP) from the	Intake of fruit and vegetables (annual kg purchased per household). This survey consists of daily collection, using an optical reader, of	Average annual intake:<br>2004: 174.2, 2005: 173.8, 2006: 171.6, 2007: 175.9, 2008: 175.6, 2009: 178.2, 2010: 186.1, 2011: 186.8, 2012: 189.2, 2013: 191.3, 2014:187.8, 2015: 180.8, 2016: 182.2.<br>The Annual Percentage Change for fruits and vegetables consumption in the different time intervals:	Selection total: 2<br>Comparability total: 0<br>Outcomes total: 1<br>**Total score: 3**

		the Ministry of Food in a representative sample of homes.<br>Sample size not stated.		World Bank. The economic decline in Spain was greater in the second part of the economic crisis.<br>Commence-ment of GR (time) (annual percentage change in GDP in brackets)<br>Time point 1: 2004–2007 (4.1)<br>Time point 2: 2008–2010 (-0.9)<br>Time point 3: 2011–2013 (-2.1)<br>Time point 4: 2014–2016 (2.7).	products that are purchased or enter the home. The results are published and broken down by food groups per household.	2004 - 2007 (before crisis): −0.1 (p value 0.831),<br>2008 - 2010 (during crisis): 2.1 (p-value <0.001)<br>2011 - 2013 (during crisis): 1.2 (p-value 0.026)<br>2014 - 2016 (after crisis): −1.9 (p-value 0.003)	
Shabnam et al., 2016	National: Pakistan<br>Local: n/a	Household Integrated Economic Survey (HIES), a nationally representative survey of rural and urban areas (14 big cities and 81 districts	Serial cross-sectional. Food budget shares of households across the years and quartiles of the expenditure distribution.<br>Quantile regression on demand equation including per capita	Exposure: Commence-ment of GR (time)<br>Time point 1: 2005-6 (n=14,863)<br>Time point 2: 2010-11<br>n= 15,191	The data on household food consumption covered a period of 14 days and 30-days call period. Food items are aggregated into budget share for	**Budget share:**<br>Milk & milk products budget share (litres) (% of expenditure):<br>2005 – 2006:<br>All: 16.49, Q1: 13.31, Q3: 18.64<br>2010-2011:<br>All: 20.75, Q1: 17.09, Q3: 24.59<br>Meat, poultry and fish budget share (kg) (% of expenditure):<br>2005 - 2006	Selection total: 3<br>Comparability total: 2<br>Outcomes total: 2<br>**Total score: 7**

		in each of the country's four provinces).<br>Mean age: 45, Female-headed households 7.5% in 2005 and 8.4% in 2010.<br>N= 14,863 and 15,191	monthly expenditure, price and household demographic characteristics.<br>and employment status), gender composition, regional and provincial dummies<br>and district fixed effects.		11 food groups:<br>milk & milk products (litres),<br>meat, poultry and fish (kg),<br>fresh fruits (kg),<br>vegetables (kg),<br>spices & condiments (kg),<br>wheat & wheat flour (kg),<br>rice (kg),<br>pulses (whole and split) (kg),<br>edible oils and fats (litres),<br>other foods (kg).<br>Price elasticity of changes in calorie intake by food group.	All: 8.56, Q1: 6.19, Q3: 11.35<br>2010-2011:<br>All: 10.04, Q1: 6.75, Q3: 13.74<br>Fresh fruits budget share (kg) (% of expenditure):<br>2005 - 2006<br>All: 2.61, Q1: 1.56, Q3: 3.93<br>2010-2011:<br>All: 2.93, Q1: 1.79, Q3: 4.51<br>Vegetable budget share (kg) (% of expenditure):<br>2005 - 2006<br>All: 7.56, Q1: 8.78, Q3: 6.14<br>2010-2011:<br>All: 9.64, Q1: 10.42, Q3: 8.21<br>Spices & condiments budget share (kg) (% of expenditure)<br>2005 - 2006<br>All: 2.72, Q1: 2.92, Q3: 2.46<br>2010-2011:<br>All: 3.63, Q1: 3.69, Q3: 3.44<br>Wheat & wheat flour budget share (kg) (% of expenditure)<br>2005 - 2006<br>All: 14.65, Q1: 20.1, Q3: 8.63<br>2010-2011:<br>All: 17.54, Q1: 23.31, Q3: 11.52<br>Rice budget share (kg) (% of expenditure)<br>2005 - 2006<br>All: 2.92, Q1: 3.41, Q3: 2.37<br>2010-2011:<br>All: 3.79, Q1: 3.99, Q3: 3.34<br>Pulses (whole and split) budget share (kg) (% of expenditure)<br>2005 - 2006	

						All: 2.09, Q1: 2.32, Q3: 1.73<br>2010-2011:<br>All: 2.91, Q1: 3.04, Q3: 2.57<br>Edible oils and fats budget share (litres) (% of expenditure)<br>2005 - 2006<br>All: 7.44, Q1: 8.16, Q3: 6.37<br>2010-2011:<br>All: 10.96, Q1: 11.99, Q3: 9.5<br>Other foods budget share (kg) (% of expenditure)<br>2005 - 2006<br>All: 4.69, Q1: 4.28, Q3: 5.59<br>2010-2011:<br>All: 7.77, Q1: 6.2, Q3: 10.44<br>**Quantile regression of changes in calorie intake to variation in prices**<br>Log price milk & milk products:<br>Estimate: –0.146 (SE 0.005) ($p < 0.10$)<br>θ = 0.10: –0.197 (SE 0.022) ($p < 0.01$);<br>θ = 0.50: –0.203 (0.021) ($p < 0.01$);<br>θ = 0.90: –0.165 (SE 0.030) ($p < 0.01$), $p = 0.052$<br>Log price meat, poultry and fish:<br>Estimate: 0.004 (SE 0.004) ($p < 0.10$)<br>θ = 0.10: –0.021 (SE 0.017);<br>θ = 0.50: –0.008 (SE 0.010);<br>θ = 0.90: –0.061 (SE 0.023) ($p < 0.01$), $p = 0.820$<br>Log price fresh fruits:<br>Estimate: 0.012 (0.002) ($p < 0.01$)<br>θ = 0.10: –0.115 (SE 0.016) ($p < 0.01$);<br>θ = 0.50: –0.082 (SE 0.007) ($p < 0.01$);<br>θ = 0.90: –0.116 (SE 0.014) ($p < 0.01$)<br>$p = 0.020$<br>Log price vegetables:	

						Estimate: –0.172 (0.011) ($p < 0.10$)<br>$\theta = 0.10$: –0.008 (SE 0.050);<br>$\theta = 0.50$: –0.083 (SE 0.031) ($p < 0.01$);<br>$\theta = 0.90$: –0.007 (SE 0.070),<br>$p = 0.313$<br>Log price spices & condiments:<br>Estimate: –0.006 (0.001) ($p < 0.10$)<br>$\theta = 0.10$: 0.041 (SE 0.011) ($p < 0.01$);<br>$\theta = 0.50$: 0.005 (SE 0.008);<br>$\theta = 0.90$: –0.032 (SE 0.012) ($p < 0.01$),<br>$p = 0.000$<br>Log price wheat & wheat flour:<br>Estimate: 0.447 (0.006) ($p < 0.01$)<br>$\theta = 0.10$: 0.147 (SE 0.027) ($p < 0.01$);<br>$\theta = 0.50$: 0.206 (SE 0.023) ($p < 0.01$);<br>$\theta = 0.90$: 0.150 (SE 0.043) ($p < 0.01$),<br>$p = 0.087$<br>Log price rice:<br>Estimate: –0.315 (0.005) ($p < 0.01$)<br>$\theta = 0.10$: –0.224 (SE 0.027) ($p < 0.01$);<br>$\theta = 0.50$: –0.205 (SE 0.014) ($p < 0.01$);<br>$\theta = 0.90$: –0.159 (SE 0.030) ($p < 0.01$),<br>$p =0.230$<br>Log price pulses (whole and split):<br>Estimate: –0.076 (0.013) ($p < 0.01$)<br>$\theta = 0.10$: –0.166 (SE 0.037) ($p < 0.01$);<br>$\theta = 0.50$: –0.251 (SE 0.028) ($p < 0.01$);<br>$\theta = 0.90$: –0.293 (SE 0.075) ($p < 0.01$),<br>$p = 0.059$<br>Log price edible oils and fats:<br>Estimate: 0.279 (0.018) ($p < 0.05$)<br>$\theta = 0.10$: –0.014 (SE 0.043);<br>$\theta = 0.50$: –0.022 (SE 0.034);<br>$\theta = 0.90$: –0.147 (SE 0.088) ($p < 0.10$),<br>$p = 0.249$<br>Log price other foods:	

						Estimate: –0.022 (0.000) (p < 0.01)<br>θ = 0.10: 0.011 (SE 0.004) (p < 0.01);<br>θ = 0.50: 0.018 (SE 0.004) (p < 0.01);<br>θ = 0.90: 0.028 (SE 0.008) (p < 0.01),<br>p = 0.093<br>Log price milk & milk products*Y2010<br>θ = 0.10: 0.085 (SE 0.036) (p < 0.01);<br>θ = 0.50: 0.115 (SE 0.026) (p < 0.01);<br>θ = 0.90: 0.156 (SE 0.044) (p < 0.01),<br>p = 0.488<br>Log price meat, poultry and fish*Y2010<br>θ = 0.10: 0.131 (SE 0.019) (p < 0.01);<br>θ = 0.50: 0.094 (SE 0.013) (p < 0.01);<br>θ = 0.90: 0.152 (SE 0.028) (p < 0.01),<br>p = 0.025<br>Log price fresh fruits*Y2010<br>θ = 0.10: 0.101 (SE 0.018) (p < 0.01);<br>θ = 0.50: 0.069 (SE 0.010) (p < 0.01);<br>θ = 0.90: 0.103 (SE 0.015) (p < 0.01),<br>p = 0.015<br>Log price vegetables*Y2010<br>θ = 0.10: –0.224 (SE 0.049) (p < 0.01);<br>θ = 0.50: –0.130 (SE 0.040) (p < 0.01);<br>θ = 0.90: –0.084 (SE 0.079) (p < 0.01),<br>p = 0.299<br>Log price spices & condiments*Y2010<br>θ = 0.10: –0.029 (SE 0.012) (p < 0.01);<br>θ = 0.50: –0.007 (SE 0.009);<br>θ = 0.90: 0.022 (SE 0.015),<br>p = 0.014<br>Log price wheat & wheat flour*Y2010<br>θ = 0.10: 0.120 (SE 0.040) (p < 0.10);<br>θ = 0.50: 0.061 (SE 0.031) (p < 0.05);<br>θ = 0.90: 0.000 (SE 0.056) (p < 0.01),<br>p = 0.029<br>Log price rice * Y2010:	

						$\theta = 0.10$: –0.054 (SE 0.028) ($p < 0.05$);<br>$\theta = 0.50$: –0.057 (SE 0.021) ($p < 0.01$);<br>$\theta = 0.90$: –0.037 (SE 0.036),<br>$p = 0.852$<br>Log price pulses*Y2010<br>$\theta = 0.10$: 0.199 (SE 0.054) ($p < 0.01$);<br>$\theta = 0.50$: 0.071 (SE 0.032) ($p < 0.01$);<br>$\theta = 0.90$: 0.056 (SE 0.091), $p=0.027$<br>Log price edible oils and fats*Y2010:<br>$\theta = 0.10$: 0.296 (SE 0.059) ($p < 0.01$);<br>$\theta = 0.50$: 0.314 (SE 0.059) ($p < 0.01$);<br>$\theta = 0.90$: 0.433 (SE 0.127) ($p < 0.01$),<br>$p = 0.482$<br>Log price other foods * Y2010:<br>$\theta = 0.10$: –0.037 (SE 0.004) ($p < 0.01$);<br>$\theta = 0.50$: –0.051 (SE 0.005) ($p < 0.01$);<br>$\theta = 0.90$: - 0.048 (SE 0.012) ($p < 0.01$),<br>$p = 0.081$<br>The positive prices of wheat and sugar indicate that these commodities are the main source of calories.<br>A vast majorities of elasticities decreased in 2010 after the food crisis, with the exception of milk and fruit, which have become less sensitive to price changes and wheat whose positive price elasticity has not changed.<br>Quintile regression reveals that changes in what elasticity are more relevant for low income prices.<br>Heterogeneous elasticities are also found for milk and oil, with meat, rice, fruit and vegetables and sugar particularly sensitive in lower income groups.	

Smed et al., 2017	National: Denmark<br>Local: n/a	GfK Panel Services Scandinavia of households of working age.<br>N=3440	Longitudinal.<br>Fixed methods econometric methods to control for unobserved heterogeneity.<br>Unemployment, single, location and number of children included in model and controlled for energy consumption.<br>If increasing CCI is associated with increasing consumption, an economic downturn is associated with decreasing consumption and vice versa.	Exposure: Consumer Confidence Interval as a proxy for economic downturn.<br>Time: January 2008 to December 2012.	Constructed consumption per individual in households by dividing each household's consumption data with weights constructed from gender- and age-dependent daily energy intake.<br>Monthly purchases at brand level amalgamated into food categories:<br>Canned and processed fish, fresh fish, frozen vegetables, fresh vegetables, fresh fruit, poultry, beef, pork, soft drinks (syrup, ice-tea, soft drinks, juice), carbonated soft drinks, processed meat (sausages and bacon), sliced meat (liver-pate, cold cuts, cold cuts salad),	Canned and processed fish<br>CCI (β1): coefficient: 1·40, P value: 0·0000<br>Fresh fish<br>CCI (β1): coefficient: 2·11, P value: 0·0000<br>Frozen Vegetables<br>CCI (β1): coefficient: 0·37, P value: 0·5640<br>Fresh vegetables<br>CCI (β1): coefficient: −61·96, P value: 0·1880<br>Fresh Fruit<br>CCI (β1): coefficient: 5·93, P value: 0·0700<br>Poultry<br>CCI (β1): coefficient: 6·57, P value: 0·0000<br>Beef<br>CCI (β1): coefficient: 1·38, P value: 0·1090<br>Pork<br>CCI (β1): coefficient: −2·13, P value: 0·0340<br>Soft drinks<br>CCI (β1): coefficient: 2·08, P value: 0·2300<br>Carbonated soft drinks<br>CCI (β1): coefficient: 3·61, P value: 0·3960<br>Processed meat<br>CCI (β1): coefficient: 1·18, P value: 0·0050<br>Sliced meat<br>CCI (β1): coefficient: 0·59, P value: 0·0220	Selection total: 4<br>Comparability total: 2<br>Outcomes total: 2<br>**Total score: 8**

					fats (margarine, butter and butter blends), oils, snacks (chips, salty and rice-based snacks, dairy snacks, sweets, pastilles and chewing-gum chocolate, marzipan and nougat), cheese (dessert cheese, block cheese), dairy (milk, yoghurt and similar products, cream), eggs, bread, flour, carbohydrates (Brown bread, white bread, crisp bread, flour, pasta and rice, cereals), processed food (Tinned dinners, Asian and Mexican food, pizza, pasta- and rice-meals, soup, desserts) and sugar products (Sugar, honey and syrup, ice cream, biscuits and	Fats<br>CCI (β1): coefficient: 1·09, P value: 0·0050<br>Oils<br>CCI (β1): coefficient: 0·01, P value: 0·9500<br>Snacks<br>CCI (β1): coefficient: −3·09, P value: 0·0000<br>Cheese<br>CCI (β1): coefficient: 1·30, P value: 0·0160<br>Dairy<br>CCI (β1): coefficient: 7·08, P value: 0·0490<br>Eggs<br>CCI (β1): coefficient: 0·84, P value: 0·1010<br>Bread, flour, carbohydrates<br>CCI (β1): coefficient: −0·58, P value: 0·6930<br>Processed food<br>CCI (β1): coefficient: 0·14, P value: 0·7550<br>Sugar products<br>CCI (β1): coefficient: 2·47, P value: 0·0020	

					cookies, marmalade, cakes, laying on chocolate and Nutella)		
Todd, 2014	National: USA<br>Local: n/a	National Health and Nutrition Examination Survey.<br>Adults born between 1946-1985 so between the ages of 20 – 64 during the study period. 48-49% male.<br>N=9,839	Serial cross-sectional. multivariate linear regression models were used to estimate the conditional changes in outcome variables. β estimated via weighted ordinary least squares with SE accounting for the complex sample design. Model (conditioning) includes age, household size, and indicators for gender, ethnicity, marital intake, data collected during weekend, and for the older cohort, education as controls.	Exposure: Commence-ment of GR (time)<br>Time point 1: 2005-2006<br>N=3,014<br>Time point 2: 2007-2008<br>N=3,294<br>Time point 3: 2009-2010<br>N=3,531	Calories from fast foods (total and %) and total/away from home snacks. From 1-day dietary recall.	**Calories from fast food:**<br><u>Unconditional and conditional differences in mean outcomes between 2005-06 and 2009- 10 calories from fast food:</u><br>Unconditional: - 83.95<br>Conditional upon age: -58.46 (difference from unconditional is statistically significant with p<0.01)<br>Conditional upon age, other demographics: -53.27<br>Conditional upon age, other demographics, and income: -52.89<br>Difference in variable mean between full model (conditional upon age, other demographics and income) and unconditional is statistically significant with p<0.01<br><u>Conditional changes by subgroups (calories from fast food):</u><br>Born 1946-85, some college or more: 2005-06: 347.56, Change 2005-06 to 2009-10: -75.92 (change is statistically significant at P<0.01)<br>Born 1946-85, no college education: 2005-06: 357.37, Change 2005-06 to 2009-10: - 17.53<br>Men, born 1946-85, no college: 2005-06: 420.12, Change 2005-06 to 2009-10: -22.30<br>Adults born before 1946:	Selection total: 3<br>Comparability total: 2<br>Outcomes total: 1<br>**Total score: 6**

						2005-06: 102.13, Change 2005-06 to 2009-10: 5.87 (estimate is different from that for the group with at least some college education, with p<0.10).<br>**Percentage calories from fast food:**<br><u>Unconditional and conditional differences in mean outcomes between 2005-06 and 2009- 10:</u><br>Unconditional: -2.92<br>Conditional upon age: -1.98 (difference from unconditional is statistically significant with p<0.01)<br>Conditional upon age, other demographics: -1.83<br>Conditional upon age, other demographics, and income: -1.83<br>Difference in variable mean between full model (conditional upon age, other demographics and income) and unconditional is statistically significant with p<0.01<br><u>Conditional changes by subgroups (% calories from fast food):</u><br>Born 1946-85, some college or more: 2005-06: 14.52, Change 2005-06 to 2009-10: -2.78 (change is statistically significant at P<0.01)<br>Born 1946-85, no college education: 2005-06: 14.31, Change 2005-06 to 2009-10: -0.35 (estimate is different from that for the group with at least some college education, with p<0.10)<br>Men, born 1946-85, no college: 2005-06: 14.07, Change 2005-06 to 2009-10: -0.36<br>Adults born before 1946:	

						2005-06: 5.69, Change 2005-06 to 2009-10: 0.00 (estimate is different from that for the group with at least some college education, with p<0.10)<br>**Total snacks:**<br><u>Unconditional and conditional differences in mean outcomes between 2005-06 and 2009- 10</u>:<br>Unconditional: -0.01<br>Conditional upon age: 0.04 (difference from unconditional is statistically significant with p<0.01)<br>Conditional upon age, other demographics: -0.02<br>Conditional upon age, other demographics, and income: -0.02<br><u>Conditional changes by subgroups (total snacks):</u><br>Born 1946-85, some college or more: 2005-06: 2.36, Change 2005-06 to 2009-10: 0.02<br>Born 1946-85, no college education: 2005-06: 2.09, Change 2005-06 to 2009-10: -0.08<br>Men, born 1946-85, no college: 2005-06: 2.18, Change 2005-06 to 2009-10: -0.12<br>Adults born before 1946:<br>2005-06: 2.05, Change 2005-06 to 2009-10: - 0.11<br>**Snacks away from home:**<br><u>Unconditional and conditional differences in mean outcomes between 2005-06 and 2009- 10:</u><br>Unconditional: -0.06<br>Conditional upon age: -0.06	

						Conditional upon age, other demographics: -0.05<br>Conditional upon age, other demographics, and income: -0.06 (difference from conditional upon age, other demographics is statistically significant with p<0.05)<br><u>Conditional changes by subgroups (FAFH snacks):</u><br>Born 1946-85, some college or more: 2005-06: 0.46, Change 2005-06 to 2009-10: - 0.07 (change is statistically significant at P<0.05)<br>Born 1946-85, no college education: 2005-06: 0.34, Change 2005-06 to 2009-10: -0.03<br>Men, born 1946-85, no college: 2005-06: 0.41, Change 2005-06 to 2009-10: -0.12 (change is statistically significant at P<0.05)<br>Adults born before 1946: 2005-06: 0.23, Change 2005-06 to 2009-10: -0.01	
Todd, 2017	National: USA<br>Local: n/a	National Health and Nutrition Examination Survey. Includes working age adults between 25-65 (n=12,129), and a secondary sample of 15-24 year olds (23-32 year olds in	Serial cross-sectional. Used multivariate linear regression models and ordinary least squares. Adjusted for age, household income relative to poverty, household size, and indicators for gender, ethnicity, marital intake, data collected during weekend, and	Exposure: Commence-ment of GR (time)<br>Time point 1: 2005-2006<br>Time point 2: 2007-2008<br>Time point 3: 2009-2010<br>Time point 4: 2013-2014	Dietary intake for one 24 h period is collected using the Automated Multiple Pass Method: Percentage energy from fast foods and total/away from home snacks.	**Percentage energy from fast foods:**<br><u>Conditional differences with their standard errors, and percentage change in estimated difference from unconditional difference in % energy from fast foods:</u><br>2007-08: β −0·95, SE: 1·07<br>2009-10: β - 1·83, SE: 0·99, percentage change: - 39<br>2011-12: β 0·42, SE: 0·95<br>2013-14 β 1·85, SE: 0·92 (estimate is statistically significant P<0·05)	Selection total: 4<br>Comparability total: 2<br>Outcomes total: 2<br>**Total score: 8**

		2013-2014) (n=5197).	for the older cohort, education as controls.			Constant: β 23·51, SE: 0.03 (estimate is statistically significant P<0·01)<br>**Total Snacks:**<br>2005-06: mean 2·28, SE: 0·04<br>2007-08: mean 2·23, SE: 0·03<br>2009-10: mean 2·24, SE: 0·05<br>2011-12: mean 2·23, SE: 0·08<br>2013-14: mean 2·24, SE: 0·08<br>**FAFH snacks:**<br>2005-06: mean 0·40, SE: 0·02<br>2007-08: mean 0·35, SE: 0·02<br>2009-10: mean 0·34, SE: 0·02 (Mean value was significantly different from that in 2005–06: P <0·05)<br>2011-12: mean 0·45, SE:0·03 (Mean value was significantly different from that in 2009–10: P <0·05)<br>2013-14: mean 0·38, SE: 0·03	
Yang et al, 2019	National: USA<br>Local: n/a	Consumer Expenditure Survey (CES). Sample size not stated.	The Bai and Perron test applied to examine trend changes. Time-Varying AIDS used to estimate the protein demand system. Iterated Seemingly Unrelated Regression (ITSUR) method used to estimate time-varying demand systems with centred prices. TV-AIDS used to calculate expenditure and price elasticities for protein sources are	Exposure: Commence-ment of GR (time) (before/after 2009)<br>Time: January 1998 and December 2016.	Estimated average weekly expenditures for each household by protein source category and multiplied these expenditures by the number of weeks in each month to obtain average monthly household expenditure. Expenditure ($) on protein products: beef, pork, poultry, fish	Bai & Perron's test indicated a structural break in protein source expenditure near October 2009, indicative that protein expenditure patterns were affected by the Great Recession.<br>**Beef**<br>Time trend estimate from Time-Varying AIDS estimation across U.S. household income quintiles.<br>Average household: −0.0002 (0.0000) (significant at 1%)<br><u>Statistical comparison of own price elasticities of demand for protein sources across different U.S. Income Strata, Pre- and Post-October 2009.</u><br>Average household:<br>pre-2009: −0.6041 (0.1494),	Selection total: 2<br>Comparability total: 1<br>Outcomes total: 2<br>**Total score: 5**

			calculated before and after the break date.		& seafood, eggs, dairy, dried beans and other meat.	post-2009: −0.5421 (0.1733)<br>(significant at 1% level)<br>Income quintile 1:<br>pre-2009: −0.7606 (0.3032),<br>post-2009: −0.7263 (0.3457)<br>Income quintile 2:<br>pre-2009: −0.4560 (0.3212),<br>post-2009: −0.4012 (0.3618)<br>Income quintile 3:<br>pre-2009: −0.9018 (0.2318),<br>post-2009: −0.8867 (0.2654)<br>Income quintile 4:<br>pre-2009: −0.6912 (0.3147),<br>post-2009: −0.6434 (0.3711)<br>Income quintile 5:<br>pre-2009: −0.4100 (0.2614),<br>post-2009: −0.2960 (0.3116)<br>(significant at 5% level)<br>Statistical comparison of expenditure elasticities of demand for protein sources across different U.S. income strata, pre- and post-October 2009.<br>Average household:<br>pre-2009: 1.4060 (0.0728),<br>post-2009: 1.4555 (0.0817)<br>(time difference significant at 1% level)<br>Income quintile 1:<br>pre-2009: 1.3101 (0.0683),<br>post-2009: 1.3449 (0.0760)<br>(time difference significant at 1% level)<br>Income quintile 2:<br>pre-2009: 1.4369 (0.0858),<br>post-2009: 1.4745 (0.0932)<br>(time difference significant at 1% level)<br>Income quintile 3:<br>pre-2009: 1.2293 (0.0718),	

						post-2009: 1.2575 (0.0806)<br>(significant at 1% level)<br>Income quintile 4:<br>pre-2009: 1.5825 (0.1060),<br>post-2009: 1.6535 (0.1189)<br>(time difference significant at 1% level)<br>Income quintile 5:<br>pre-2009: 1.5698 (0.0909),<br>post-2009: 1.6486 (0.1035)<br>(time difference significant at 1% level)<br>**Pork**<br>Time trend estimate from Time-Varying AIDS estimation across U.S. household income quintiles.<br>Average household: −0.0001 (0.0000)<br><u>Statistical comparison of own price elasticities of demand for protein sources across different U.S. Income Strata, Pre- and Post-October 2009.</u><br>Average household:<br>pre-2009: −0.7140 (0.2076),<br>post-2009: −0.6890 (0.2211)<br>(time difference significant at 10% level)<br>Income quintile 1:<br>pre-2009: −0.6760 (0.4027),<br>post-2009: −0.6372 (0.4381)<br>Income quintile 2:<br>pre-2009: −1.1869 (0.4249),<br>post-2009: −1.1948 (0.4593)<br>Income quintile 3:<br>pre-2009: −1.3935 (0.3665),<br>post-2009: −1.4310 (0.4011)<br>Income quintile 4:<br>pre-2009: −0.6596 (0.4272),<br>post-2009: −0.6441 (0.4408)	

						Income quintile 5:<br>pre-2009: 0.0751 (0.4123),<br>post-2009: −0.1484 (0.4386)<br>(time difference significant at 1% level)<br>Statistical comparison of expenditure elasticities of demand for protein sources across different U.S. income strata, pre- and post-October 2009.<br>Average household:<br>pre-2009: 1.3650 (0.0676),<br>post-2009: 1.3932 (0.0728)<br>(time difference significant at 1% level)<br>Income quintile 1:<br>pre-2009: 1.2193 (0.0620),<br>post-2009: 1.2405 (0.0680)<br>(time difference significant at 1% level)<br>Income quintile 2:<br>pre-2009: 1.3000 (0.0768),<br>post-2009: 1.3278 (0.0839)<br>(time difference significant at 1% level)<br>Income quintile 3:<br>pre-2009: 1.1723 (0.0765),<br>post-2009: 1.1895 (0.0841)<br>(time difference significant at 5% level)<br>Income quintile 4:<br>pre-2009: 1.2316 (0.0938),<br>post-2009: 1.2409 (0.0976)<br>(time difference significant at 5% level)<br>Income quintile 5:<br>pre-2009: 1.1462 (0.0908),<br>post-2009: 1.1562 (0.0970)<br>**Poultry**<br>Time trend estimate from Time-Varying AIDS estimation across U.S. household income quintiles.<br>Average household: −0.0001 (0.0001)	

						<u>Statistical comparison of own price elasticities of demand for protein sources across different U.S. Income Strata, Pre- and Post-October 2009.</u><br>Average household:<br>pre-2009: −0.7641 (0.3076),<br>post-2009: −0.7701 (0.3004)<br>Income quintile 1:<br>pre-2009: −0.1358 (0.5678),<br>post-2009: −0.1883 (0.5342)<br>Income quintile 2:<br>pre-2009: −1.4817 (0.5544),<br>post-2009: −1.4548 (0.5215)<br>Income quintile 3:<br>pre-2009: −0.1674 (0.5484),<br>post-2009: −0.1851 (0.5323)<br>Income quintile 4:<br>pre-2009: −0.9748 (0.5758),<br>post-2009: −0.9781 (0.5759)<br>Income quintile 5:<br>pre-2009: −0.8643 (0.4926),<br>post-2009: −0.8673 (0.4942)<br><u>Statistical comparison of expenditure elasticities of demand for protein sources across different U.S. income strata, pre- and post-October 2009.</u><br>Average household:<br>pre-2009: 0.9774 (0.0782),<br>post-2009: 0.9779 (0.0763)<br>Income quintile 1:<br>pre-2009: 0.9471 (0.0676),<br>post-2009: 0.9503 (0.0636)<br>Income quintile 2:<br>pre-2009: 1.0904 (0.0848),<br>post-2009: 1.0852 (0.0799)<br>Income quintile 3:	

						pre-2009: 1.2497 (0.0886),<br>post-2009: 1.2430 (0.0862)<br>(significant at 1% level)<br>Income quintile 4:<br>pre-2009: 0.9029 (0.1006),<br>post-2009: 0.9030 (0.1004)<br>Income quintile 5:<br>pre-2009: 0.8755 (0.0901),<br>post-2009: 0.8754 (0.0902)<br>**Fish and seafood**<br>Time trend estimate from Time-Varying AIDS estimation across U.S. household income quintiles.<br>Average household: 0.0001 (0.0000)<br>(significant at 10%)<br><u>Statistical comparison of own price elasticities of demand for protein sources across different U.S. Income Strata, Pre- and Post-October 2009.</u><br>Average household:<br>pre-2009: −1.6979 (0.1884),<br>post-2009: −1.7217 (0.1949)<br>(time difference significant at 1% level)<br>Income quintile 1:<br>pre-2009: −1.5358 (0.4892),<br>post-2009: −1.5438 (0.4882)<br>Income quintile 2:<br>pre-2009: −1.7316 (0.4272),<br>post-2009: −1.7620 (0.4465)<br>Income quintile 3:<br>pre-2009: −2.0454 (0.3689),<br>post-2009: −2.0847 (0.3836)<br>(time difference significant at 1% level)<br>Income quintile 4:<br>pre-2009: −1.8858 (0.3658),<br>post-2009: −1.9411 (0.3886)	

						(time difference significant at 5% level)<br>Income quintile 5:<br>pre-2009: −1.4240 (0.3158),<br>post-2009: -1.4365 (0.3256)<br>Statistical comparison of expenditure elasticities of demand for protein sources across different U.S. income strata, pre- and post-October 2009.<br>Average household:<br>pre-2009: 1.0899 (0.0940),<br>post-2009: 1.0927 (0.0970)<br>Income quintile 1:<br>pre-2009: 1.3160 (0.1189),<br>post-2009: 1.3136 (0.1179)<br>(time difference significant at 1% level)<br>Income quintile 2:<br>pre-2009: 0.9174 (0.1167),<br>post-2009: 0.9136 (0.1221)<br>Income quintile 3:<br>pre-2009: 1.2136 (0.1154),<br>post-2009: 1.2212 (0.1195)<br>(time difference significant at 10% level)<br>Income quintile 4:<br>pre-2009: 1.0206 (0.1336),<br>post-2009: 1.0219 (0.1419)<br>Income quintile 5:<br>pre-2009: 1.0571 (0.1164),<br>post-2009: 1.0588 (0.1198)<br>**Eggs**<br>Time trend estimate from Time-Varying AIDS estimation across U.S. household income quintiles.<br>Average household: 0.0001 (0.0000) (significant at 1%)	

						Statistical comparison of own price elasticities of demand for protein sources across different U.S. Income Strata, Pre- and Post-October 2009.<br>Average household:<br>pre-2009: −0.1657 (0.0598),<br>post-2009: −0.3660 (0.0454)<br>(time difference significant at 1% level)<br>Income quintile 1:<br>pre-2009: −0.0681 (0.1242),<br>post-2009: −0.2561 (0.0995)<br>(time difference significant at 1% level)<br>Income quintile 2:<br>pre-2009: −0.3828 (0.1150),<br>post-2009: −0.5173 (0.0903)<br>(time difference significant at 1% level)<br>Income quintile 3:<br>pre-2009: −0.0679 (0.1331),<br>post-2009: −0.2808 (0.1027)<br>(time difference significant at 1% level)<br>Income quintile 4:<br>pre-2009: −0.0232 (0.1220),<br>post-2009: −0.2732 (0.0909)<br>(time difference significant at 1% level)<br>Income quintile 5:<br>pre-2009: −0.2898 (0.1160),<br>post-2009: −0.4888 (0.0833)<br>(time difference significant at 1% level)<br>Statistical comparison of expenditure elasticities of demand for protein sources across different U.S. income strata, pre- and post-October 2009.<br>Average household:<br>pre-2009: 0.9406 (0.0762),<br>post-2009: 0.9549 (0.0579)<br>Income quintile 1:	

						pre-2009: 0.7447 (0.0747),<br>post-2009: 0.7958 (0.0598)<br>(time difference significant at 1% level)<br>Income quintile 2:<br>pre-2009: 0.6984 (0.0848),<br>post-2009: 0.7637 (0.0664)<br>(time difference significant at 1% level)<br>Income quintile 3:<br>pre-2009: 0.9371 (0.1111),<br>post-2009: 0.9515 (0.0858)<br>Income quintile 4:<br>pre-2009: 0.8624 (0.1079),<br>post-2009: 0.8976 (0.0803)<br>Income quintile 5:<br>pre-2009: 0.7619 (0.1078),<br>post-2009: 0.8291 (0.0774)<br>(time difference significant at 5% level)<br>**Dairy products**<br>Time trend estimate from Time-Varying AIDS estimation across U.S. household income quintiles.<br>Average household: 0.0001 (0.0000) (significant at 1%)<br><u>Statistical comparison of own price elasticities of demand for protein sources across different U.S. Income Strata, Pre- and Post-October 2009.</u><br>Average household:<br>pre-2009: 0.0640 (0.0869),<br>post-2009: 0.0306 (0.0846)<br>(time difference significant at 1% level)<br>Income quintile 1:<br>pre-2009: 0.0908 (0.2120),<br>post-2009: 0.0668 (0.2080)<br>(time difference significant at 1% level)<br>Income quintile 2:	

						pre-2009: −0.3613 (0.1810),<br>post-2009: −0.3871 (0.1769)<br>(time difference significant at 1% level)<br>Income quintile 3:<br>pre-2009: −0.3235 (0.1761),<br>post-2009: −0.3502 (0.1698)<br>(time difference significant at 1% level)<br>Income quintile 4:<br>pre-2009: 0.1264 (0.1605),<br>post-2009: 0.1004 (0.1577)<br>(time difference significant at 1% level)<br>Income quintile 5:<br>pre-2009: 0.2733 (0.1428),<br>post-2009: 0.2362 (0.1392)<br>(time difference significant at 1% level)<br><u>Statistical comparison of expenditure elasticities of demand for protein sources across different U.S. income strata, pre- and post-October 2009.</u><br>Average household:<br>pre-2009: 0.6028 (0.0371),<br>post-2009: 0.6194 (0.0355)<br>(significant at 1% level)<br>Income quintile 1:<br>pre-2009: 0.6774 (0.0428),<br>post-2009: 0.6882 (0.0413)<br>(time difference significant at 1% level)<br>Income quintile 2:<br>pre-2009: 0.6085 (0.0454),<br>post-2009: 0.6242 (0.0436)<br>(time difference significant at 1% level)<br>Income quintile 3:<br>pre-2009: 0.6819 (0.0471),<br>post-2009: 0.6979 (0.0447)<br>(significant at 1% level)<br>Income quintile 4:	

						pre-2009: 0.6181 (0.0468),<br>post-2009: 0.6309 (0.0452)<br>(time difference significant at 1% level)<br>Income quintile 5:<br>pre-2009: 0.6295 (0.0433),<br>post-2009: 0.6455 (0.0414)<br>(time difference significant at 1% level)<br>**Dried beans**<br>Time trend estimate from Time-Varying AIDS estimation across U.S. household income quintiles.<br>Average household: 0.0000 (0.0000)<br>Statistical comparison of own price elasticities of demand for protein sources across different U.S. Income Strata, Pre- and Post-October 2009.<br>Average household:<br>pre-2009: 0.0922 (0.5580),<br>post-2009: −0.3399 (0.3369)<br>(time difference significant at 10% level)<br>Income quintile 1:<br>pre-2009: 1.7225 (1.1388),<br>post-2009: 0.8141 (0.7593)<br>(significant at 5% level)<br>Income quintile 2:<br>pre-2009: −1.3724 (1.0043),<br>post-2009: −1.2381 (0.6463)<br>Income quintile 3:<br>pre-2009: −1.0460 (1.0277),<br>post-2009: −1.0277 (0.6535)<br>Income quintile 4:<br>pre-2009: −0.2507 (1.1989),<br>post-2009: −0.5834 (0.6665)<br>Income quintile 5:<br>pre-2009: 1.2870 (1.1446),	

						post-2009: 0.2059 (0.6036)<br>(time difference significant at 5% level)<br>Statistical comparison of expenditure elasticities of demand for protein sources across different U.S. income strata, pre- and post-October 2009.<br>Average household:<br>pre-2009: 0.6096 (0.3401),<br>post-2009: 0.7642 (0.2055)<br>Income quintile 1:<br>pre-2009: 1.3432 (0.3264),<br>post-2009: 1.2286 (0.2174)<br>Income quintile 2:<br>pre-2009: −0.1312 (0.3484),<br>post-2009: 0.2698 (0.2249)<br>(time difference significant at 1% level)<br>Income quintile 3:<br>pre-2009: 0.6377 (0.3940),<br>post-2009: 0.7694 (0.2508)<br>Income quintile 4:<br>pre-2009: 0.9585 (0.5122),<br>post-2009: 0.9769 (0.2848)<br>Income quintile 5:<br>pre-2009: 1.0682 (0.4903),<br>post-2009: 1.0360 (0.2585)<br>**Other meat**<br>Time trend estimate from Time-Varying AIDS estimation across U.S. household income quintiles.<br>Average household: 0.0000 (0.0000)<br>Statistical comparison of own price elasticities of demand for protein sources across different U.S. Income Strata, Pre- and Post-October 2009.<br>Average household:<br>pre-2009: −1.1329 (0.1851),	

						post-2009: −1.1229 (0.1714)<br>Income quintile 1:<br>pre-2009: −1.0183 (0.4053),<br>post-2009: −1.0181 (0.3962)<br>Income quintile 2:<br>pre-2009: −1.1625 (0.4210),<br>post-2009: −1.1608 (0.4149)<br>Income quintile 3:<br>pre-2009: −1.0085 (0.3379),<br>post-2009: −1.0074 (0.3086)<br>Income quintile 4:<br>pre-2009: −1.1367 (0.3979),<br>post-2009: −1.1215 (0.3559)<br>Income quintile 5:<br>pre-2009: −1.2979 (0.3386),<br>post-2009: −1.2713 (0.3086)<br><u>Statistical comparison of expenditure elasticities of demand for protein sources across different U.S. income strata, pre- and post-October 2009.</u><br>Average household:<br>pre-2009: 0.8462 (0.0703),<br>post-2009: 0.8568 (0.0655)<br>(time difference significant at 5% level)<br>Income quintile 1:<br>pre-2009: 0.8861 (0.0764),<br>post-2009: 0.8882 (0.0750)<br>Income quintile 2:<br>pre-2009: 0.9458 (0.0906),<br>post-2009: 0.9465 (0.0895)<br>Income quintile 3:<br>pre-2009: 0.7464 (0.0875),<br>post-2009: 0.7661 (0.0807)<br>(time difference significant at 1% level)<br>Income quintile 4:<br>pre-2009: 0.8189 (0.1014),	

						post-2009: 0.8370 (0.0913)<br>(time difference significant at 10% level)<br>Income quintile 5:<br>pre-2009: 1.0239 (0.0969),<br>post-2009: 1.0218 (0.0881)	
Macronutrient intake							
Bonaccio et al, 2014<br>Ref:	National: Italy<br>Local: the Molise region	Moli-Sani study. Adults aged over 35 (mean age 54.4); percentage male: 47.30%. Randomly recruited.<br>Total n=21,001.	Serial cross-sectional study of participants recruited before and after the recession. Means and p-values of grams/day and % contribution adjusted for age and sex.	Exposure: Commence-ment of GR (time)<br>Time point 1: Recruited in 2005-2006 (n=6999)<br>Time point 2: Recruited in 2007-2010 (n=14,002)	Grams/day of carbohydrates, saturated fats and fibre (means + SD), carbohydrate, protein and fat contribution to energy intake (%, means + SD)	**Carbohydrates**:<br>Grams/day: 2005/6: 261.8 (95.1)<br>2007-2010: 258.5 (86.5)<br>p-value <0.0001<br>% contribution:<br>2005/6: 47.6, 2007-2010: 46.9<br>p-value <0.0001<br>**Protein:**<br>% contribution:<br>2005/6: 15.5, 2007-2010: 15.8<br>p-value <0.0001<br>**Saturated fats:**<br>Grams/day: 2005/6: 27.7 (11.0)<br>2007-2010: 28.1 (9.8)<br>p-value <0.0001<br>**Fats:**<br>% contribution:<br>2005/6: 31.8, 2007-2010:32.3<br>p-value <0.0001<br>**Fibre:**<br>Grams/day: 2005/6: 21.8 (7.6)<br>2007-2010: 20.0 (6.5)<br>p-value <0.0001	Selection total: 3<br>Comparability total: 2<br>Outcomes total: 2<br>**Total score: 7**
Çirakli & Yildirim, 2019	National: Turkey<br>Local: n/a	OECD data.	Used ARDL bounds testing and cointegration analysis including OLS: unit root tests, Augmented Dickey-	Exposure: Commence-ment of GR (time), using real GDP, unemploy-	Used OECD data on annual per capita sugar consumption (kg)	Result of ADF Unit Root Test:<br>level values t: 0.49, p 0.817; first difference values: t: -6.31 (significant at 1% level), p: 0.000<br>Result of ARDL correction model:	Selection total: 2<br>Comparability total: 1<br>Outcomes total: 1

			Fuller Test, Unrestricted Error Correction Model using OLS and Wald test, estimation of long-term coefficients and creation of Error Correction Model.	ment rates, inflation rate as indicators of crises.<br>Time: 1974-2015 (42 time points), covering economic crises in 1994, 2001, 2009).		Change D 2009: coefficient: 0.188, t: 3.369, p: 0.002<br>(significant positive impact on sugar consumption).	**Total score: 4**
Florkowski 2012	National: Poland<br>Local: n/a	The study is based on data obtained from annual Glowny Urzad Statystyczny surveys of Polish households.	Serial cross-sectional. Households average yearly expenditure.	Exposure: Commence-ment of GR (time)<br>Time point 1: 2004<br>Time point 2: 2005<br>Time point 3: 2006<br>Time point 4: 2007<br>Time point 5: 2008	Average expenditure on sugar (in zloty).	2006:<br>All households, n= 17878, average expenditure: 16.03<br>Households above minimum income, n= 8884, average expenditure: 16.14<br>Households at or below minimum income, n= 8994, average expenditure: 15.92<br>2007:<br>All households, n= 15554, average expenditure: 15.44<br>Households above minimum income, n= 8098, average expenditure: 15.77<br>Households at or below minimum income, n= 7456, average expenditure: 15.08<br>2008:<br>All households, n= 15163, average expenditure: 13.90<br>Households above minimum income, n= 8277, average expenditure: 14.17<br>Households at or below minimum income, n= 6886, average expenditure: 13.57	Selection total: 2<br>Comparability total: 0<br>Outcomes total: 1<br>**Total score: 3**

Griffith et al, 2016a	National: UK<br>Local: n/a	Kantar Worldpanel data for UK households. n=14,694.	Longitudinal study. HEI scores/calorie shares/grams per 100g and percentage change.	Exposure: Commence-ment of GR (time)<br>Time point 1: 2005-2007,<br>Time point 2: 2010-12	Healthy Eating (HEI) score for sodium and saturated fats. Share of calories from protein, saturated fat, unsaturated fat, sugar, non-sugar carbohydrates. Grams per 100g for salt and fibre. Outcome data of food purchases from all types of stores using an electronic hand-held scanner in the home.	**HEI score – sodium:**<br>Max score: 10<br>Mean in 2005-7: 6.42<br>Change to 2010-12: 0.93<br>Percentage change to 2010-12: 14.5<br>**HEI score – saturated fats:**<br>Max score: 10<br>Mean in 2005-7: 2.70<br>Change to 2010-12: 0.27<br>Percentage change to 2010-12: 10.0<br>**Share of calories from protein:**<br>2005-7: 14.88<br>2010-12: 14.76<br>Change to 2010-12: - 0.12,<br>Percentage change: - 0.81<br>**Share of calories from saturated fat:**<br>2005-7: 14.83<br>2010-12: 14.59<br>Change to 2010-12: - 0.23,<br>Percentage change: - 1.57<br>**Share of calories from unsaturated fat:**<br>2005-7: 22.64<br>2010-12:22.79<br>Change to 2010-12: 0.15,<br>Percentage change: 0.67<br>**Share of calories from sugar:**<br>2005-7: 22.73<br>2010-12:22.82<br>Change to 2010-12: 0.09,<br>Percentage change: 0.41<br>**Share of calories from non-sugar carbohydrates:**<br>2005-7: 24.92<br>2010-12: 25.03<br>Change to 2010-12: 0.11,	Selection total: 3<br>Comparability total: 2<br>Outcomes total: 2<br>**Total score: 7**

						Percentage change: 0.43<br>**Grams per 100g – salt:**<br>2005-7: 0.50<br>2010-12: 0.49<br>Change to 2010-12: - 0.00,<br>Percentage change: - 0.10<br>**Grams per 100g – fibre:**<br>2005-7: 1.12<br>2010-12: 1.19<br>Change to 2010-12: 0.07,<br>Percentage change: 6.32	
Griffith et al, 2013	National: UK<br>Local: n/a	Kantar Worldpanel data.<br>N=15,850.	Longitudinal. Regressed variables on three time-period dummies and controlled for month and household fixed, estimating regression separately by household.	Exposure: Commence-ment of GR (time)<br>Time point 1: 2005-2007<br>Time point 2: 2008-2009<br>Time point 3:	Change in saturated fat, sugar, salt and protein (g per 100g).<br>Participants record spending on all grocery purchases brought into the home via an electronic hand-held scanner in the home.	**Change in saturated fat:**<br>Single non-pensioners:<br>2008-09: 0.08, 2010-12: 0.09<br>Single pensioners:<br>2008-09: 0.11, 2010-12: 0.12<br>Couple non-pensioners:<br>2008-09: 0.05, 2010-12: 0.07<br>Couple pensioners:<br>2008-09:0.08, 2010-12: 0.12<br>Multi-adult households:<br>2008-09: 0.04, 2010-12: 0.07<br>Single parents:<br>2008-09: 0.04, 2010-12: 0.08<br>2+ adults, young children:<br>2008-09: 0.03, 2010-12: 0.05<br>2+ adults, older children:<br>2008-09: 0.02, 2010-12: 0.03<br>All households:<br>2008-09: 0.06, 2010-12: 0.08<br>All changes are statistically different from zero at the 99% level.<br>**Change in sugar:**<br>Single non-pensioners:<br>2008-09: 0.08, 2010-12: 0.09,<br>Single pensioners:	Selection total: 3<br>Comparability total: 2<br>Outcomes total: 2<br>**Total score: 7**

						2008-09: 0.11, 2010-12: 0.12,<br>Couple non-pensioners:<br>2008-09: 0.05, 2010-12: 0.07,<br>Couple pensioners:<br>2008-09:0.08, 2010-12: 0.12,<br>Multi-adult households:<br>2008-09: 0.04, 2010-12: 0.07<br>Single parents:<br>2008-09: 0.04, 2010-12: 0.08<br>2+ adults, young children:<br>2008-09: 0.03, 2010-12: 0.05<br>2+ adults, older children:<br>2008-09:0.02, 2010-12: 0.03<br>All households:<br>2008-09: 0.06, 2010-12: 0.08<br>All changes are statistically different from zero at the 99% level, apart from '2+ adults, older children (2008–09 and 2010–12)'.<br>**Change in salt:**<br>Single non-pensioners:<br>2008-09: 0.17, 2010-12: 0.20<br>Single pensioners:<br>2008-09: 0.33, 2010-12: 0.35<br>Couple non-pensioners:<br>2008-09: 0.16, 2010-12: 0.29<br>Couple pensioners:<br>2008-09: 0.21, 2010-12: 0.30<br>Multi-adult households:<br>2008-09: 0.16, 2010-12: 0.18<br>Single parents:<br>2008-09: 0.13, 2010-12: 0.26<br>2+ adults, young children:<br>2008-09: 0.29, 2010-12: 0.44<br>2+ adults, older children:<br>2008-09: 0.01, 2010-12: 0.00	

						All households:<br>2008-09: 0.20, 2010-12: 0.27<br>All changes are statistically different from zero at the 99% level.<br>**Change in protein:**<br>Single non-pensioners:<br>2008-09: 0.09, 2010-12: 0.12,<br>Single pensioners:<br>2008-09: 0.08, 2010-12: 0.08,<br>Couple non-pensioners:<br>2008-09: 0.04, 2010-12: 0.05,<br>Couple pensioners:<br>2008-09:0.09, 2010-12:0.09,<br>Multi-adult households:<br>2008-09: 0.05, 2010-12:0.11<br>Single parents:<br>2008-09: 0.12, 2010-12: 0.21<br>2+ adults, young children:<br>2008-09: 0.10, 2010-12: 0.15<br>2+ adults, older children:<br>2008-09: 0.14, 2010-12: 0.20<br>All households:<br>2008-09: 0.08, 2010-12:0.11<br>All changes are statistically different from zero at the 99% level.	
Hasan, 2019	National: Bangladesh<br>Local: n/a	Bangladesh Household Income and Expenditure Survey.<br>Repeated cross-sectional study using two-stage stratified random sampling.	Serial cross-sectional design.<br>The study used difference-in-difference framework and OLS models including district fixed effects and employing clustered standard errors (weighted).	Exposure: Commence-ment of GR (time).<br>Time point 1: 2005 (n=4,978), time point 2: 2010 (n=6,744).	Calorie intake from protein (per capita per day), constructed using calorie content of food items.	2010 coefficient: 8.31 (5.38) (not significant)	Selection total: 3<br>Comparability total: 2<br>Outcomes total: 2<br>**Total score: 7**

		Analysis was done for those who buy rice (compared to autarkic households and rice sellers, but there was no significant difference between these types).<br>n=11,722					
Marcotte-Chenard	National: USA<br>Local: n/a	National Health and Nutrition Examination Survey. Adults aged between 20-85 (average age 49), 48.1% male.<br>N=38,541	Serial cross-sectional study.<br>Factorial ANOVAs (post hoc test and contrast) conducted to compare 1999-2006 intervals to 2006-2007 intervals.	Exposure: Commence-ment of GR (time)<br>Time point 1: 1999-2006<br>Time point 2: 2007-2008.	24-hr dietary recall used to calculate average daily protein, carbohydrate, fats, sodium and sugar intake in men and women.	**Protein:**<br>Men:<br>1999-2006: 93 ± 46, 2007-2008: 89 ± 45 g/day; P = 0.0001.<br>Women:<br>1999-2006: 68 ± 33, 2007-2008: 66 ± 32 g/day; P = 0.017.<br>**Carbohydrates:**<br>Men:<br>1999-2006: 289 ± 137, 2007-2008: 279.0 ± 128 g/day; P = 0.0001.<br>Women:<br>1999-2006: 229 ± 107, 2007-2008: 213 ± 88 g/day; P = 0.0001.<br>**Fats:**<br>Men:<br>Not significant – not reported.<br>Women:<br>1999-2006: 66 ± 36, 2007-2008: 63 ± 31 g/day; P = 0.0001.<br>**Sugar:**<br>Men:	Selection total: 3<br>Comparability total: 1<br>Outcomes total: 2<br>**Total score: 6**

						2001-2006: 137.7 ± 92.7, 2007-2008: 127.7 ± 92.7 g/day; P = 0.0001.<br>Women:<br>2001-2006: 110.9 ± 70.6, 2007-2008: 101.4 ± 65.8g/day; P = 0.0001.<br>**Sodium:**<br>Men:<br>1999-2006: 3718 ± 1923, 2007-2008: 3602 ± 1930 mg/day; P = 0.002<br>Women:<br>1999-2006: 2831 ± 1410, 2007-2008: 2668 ± 1415 mg/day; P = 0.0001.	
Martin-Prevel et al, 2012	National: Burkina Faso<br>Local: Ouaga-dougou	Households randomly selected from Ouagadougou census. Average age of household head: 42, household head 86.8% (2007) and 87.8% (2008) male.	Serial cross-sectional. Changes in food-related indicators between 2007 and 2008 were analyzed using chi-square tests for proportions.	Exposure: Commence-ment of GR (time)<br>Time Point 1: July 2007 (n=3017)<br>Time Point 2: July 2008 (n=3002)	24-hour food expenditure. Obtain a food basket price for sugar per day calculating by summing mean price per kilogram and prices multiplied by daily sugar consumption.	2007 to 2008: 3% decrease in sugar.	Selection total: 3<br>Comparability total: 2<br>Outcomes total: 2<br>**Total score: 7**
Mohseni-Cheraglou, 2016	Global: 63 countries (and 100 recessions).	Reinhart and Rogoff's dataset on financial crises; FAOSTAT data 2010. 93 observations.	Investigating whether growth rates of different variables are affected by financial crises.	Exposure: value of a currency falling by 15% or more against the US dollar or banking distress including closures, mergers and	Protein Intake per Capita Per Day (g)	Average change in growth rate (t-test): -3.2 ($p < 0.01$)<br>Crises with recessions, average change in growth rate (t-test): -4.0 ($p < 0.01$)<br>Crises without recessions, average change in growth rate (t-test): -2.3 ($p < 0.01$)<br>Calories reduced in economic crises with and without recessions.	Selection total: 2<br>Comparability total: 0<br>Outcomes total: 1<br>**Total score: 3**

				government takeovers. Data from 1981-2007.			
Shabnam et al., 2016	National: Pakistan<br>Local: n/a	Household Integrated Economic Survey (HIES), a nationally representative survey of rural and urban areas (14 big cities and 81 districts in each of the country's four provinces).<br>Mean age: 45, Female-headed households 7.5% in 2005 and 8.4% in 2010.<br>N= 14,863 and 15,191	Serial cross-sectional. Food budget shares of households across the years and quartiles of the expenditure distribution.<br>Quantile regression on demand equation including per capita monthly expenditure, price and household demographic characteristics.	Exposure: Commence-ment of GR (time)<br>Time point 1: 2005-6 (n=14,863)<br>Time point 2: 2010-11<br>n= 15,191	The data on household food consumption covered a period of 14 days and 30-days call period.<br>Price elasticity of carbohydrates, fats and proteins; sugar and sugar preparations budget share (kg) (% of expenditure).	Price elasticity for carbohydrates:<br>2005-2006: -0.003<br>2010-2011: -0.143<br>Price elasticity for fats:<br>2005-2006: –0.004<br>2010-2011: –0.302<br>Price elasticity for proteins:<br>2005-2006: –0.001<br>2010-2011: -0.183<br>Sugar and sugar preparations budget share (kg) (% of expenditure):<br>2005 - 2006<br>All: 7.17, Q1: 8.35, Q3: 5.73<br>2010-2011:<br>All: 9.65, Q1: 11.33, Q3: 7.73<br>Log price sugar & sugar preparations:<br>Estimate: 0.112 (0.006)<br>θ = 0.10: 0.125 (SE 0.018) ($p < 0.01$);<br>θ = 0.50: 0.166 (SE 0.018) ($p < 0.01$);<br>θ = 0.90: 0.105 (SE 0.028) ($p < 0.01$), p = 0.0107<br>Log price sugar & sugar preparations* Y2010: θ = 0.10: –0.095 (SE 0.024) ($p < 0.01$);<br>θ = 0.50: –0.123 (SE 0.021) ($p < 0.01$);<br>θ = 0.90: –0.068 (SE 0.029) ($p < 0.01$), p = 0.0634	Selection total: 3<br>Comparability total: 2<br>Outcomes total: 2<br>**Total score: 7**
Smed et al., 2017	National: Denmark<br>Local: n/a	GfK Panel Services Scandinavia of households of working age.	Longitudinal.<br>Fixed methods econometric methods to control for unobserved	Exposure: Consumer Confidence Interval as a proxy for	Constructed consumption per individual in households by dividing each	**Total fat:**<br>CCI ($\beta1$) coefficient: 3·82, P value: 0·0000	Selection total: 4<br>Comparability total: 2

		N=3440	heterogeneity. Unemployment, single, location and number of children included in model. If increasing CCI is associated with increasing consumption, an economic downturn is associated with decreasing consumption and vice versa.	economic downturn. Time: January 2008 to December 2012.	household's consumption data with weights constructed from gender- and age-dependent daily energy intake. Monthly purchases at brand level concatenated into macronutrients: total fat, saturated fat, added sugar, fibre, carbohydrates and proteins (grams/person/month).	CCI (β1) controlled for energy consumption: coefficient: 3·61, P value: 0·0000<br>**Saturated fat**<br>CCI (β1): coefficient: 1·90, P value: 0·0000<br>CCI (β1) controlled for energy consumption: coefficient: 1·80 P value: 0·0000<br>**Added sugar**<br>CCI (β1) coefficient: −2·49, P value: 0·7960<br>CCI (β1) controlled for energy consumption: coefficient: −6·27 P value: 0·0180<br>**Fibre**<br>CCI (β1) coefficient: 0·09, P value: 0·6660<br>CCI (β1) controlled for energy consumption: coefficient: 0·05 P value: 0·8020<br>**Carbohydrates**<br>CCI (β1) coefficient: −2·78, P value: 0·7830<br>CCI (β1) controlled for energy consumption: coefficient: −7·05, P value: 0·0000<br>**Proteins**<br>CCI (β1) coefficient: 3·37, P value: 0·0000<br>CCI (β1) controlled for energy consumption: coefficient: 3·20, P value: 0·0000	Outcomes total: 2<br>**Total score: 8**
Todd, 2014	National: USA<br>Local: n/a	National Health and Nutrition	Serial cross-sectional. multivariate linear regression models	Exposure: Commence-	Dietary intake for one 24 h period is collected using	**Percentage calories from fat**	Selection total: 3

		Examination Survey.<br>Adults born between 1946-1985 so between the ages of 20 – 64 during the study period. 48-49% male.<br>N=9,839	were used to estimate the conditional changes in outcome variables. β estimated via weighted ordinary least squares with SE accounting for the complex sample design. Model (conditioning) includes age, household size, and indicators for gender, ethnicity, marital intake, data collected during weekend, and for the older cohort, education as controls.	ment of GR (time)<br>Time point 1: 2005-2006<br>N=3,014<br>Time point 2: 2007-2008<br>N=3,294<br>Time point 3: 2009-2010<br>N=3,531	the Automated Multiple Pass Method:<br>Share of calories from fat, saturated fat and cholesterol (mg) and fibre (g) consumption.	<u>Unconditional and conditional differences in mean outcomes between 2005-06 and 2009- 10</u><br>Unconditional: -0.96<br>Conditional upon age: - 1.26 (difference from unconditional is statistically significant with p<0.01)<br>Conditional upon age, other demographics: -1.12<br>Conditional upon age, other demographics, and income: -1.15<br><u>Conditional changes by subgroups:</u><br>Born 1946-85, some college or more: 2005-06: 34.36, Change 2005-06 to 2009-10: -1.15 (change is statistically significant at P<0.05)<br>Born 1946-85, no college education: 2005-06: 32.81, Change 2005-06 to 2009-10: -1.15 (change is statistically significant at P<0.05)<br>Men, born 1946-85, no college: 2005-06: 32.64, Change 2005-06 to 2009-10: -1.40 (change is statistically significant at P<0.05)<br>Adults born before 1946:<br>2005-06: 34.01, Change 2005-06 to 2009-10: 0.09 (estimate is different from that for the group with at least some college education, with p<0.10)<br>**Percentage calories from saturated fat**<br><u>Unconditional and conditional differences in mean outcomes between 2005-06 and 2009- 10 % calories from sat fat:</u><br>Unconditional: -0.64	Comparability total: 2<br>Outcomes total: 1<br>**Total score: 6**

						Conditional upon age: -0.71 (difference from unconditional is statistically significant with p<0.01)<br>Conditional upon age, other demographics: -0.67<br>Conditional upon age, other demographics, and income: -0.67<br><u>Conditional changes by subgroups:</u><br>Born 1946-85, some college or more: 2005-06: 11.47, Change 2005-06 to 2009-10: -0.76 (change is statistically significant at P<0.01)<br>Born 1946-85, no college education: 2005-06: 11.02, Change 2005-06 to 2009-10: -0.57 (change is statistically significant at P<0.01)<br>Men, born 1946-85, no college: 2005-06: 10.91, change 2005-06 to 2009-10: -0.50 (change is statistically significant at P<0.1)<br>Adults born before 1946:<br>2005-06: 11.39, Change 2005-06 to 2009-10: - 0.37 (change is statistically significant at P<0.05)<br>**Cholesterol consumption:**<br><u>Unconditional and conditional differences in mean outcomes between 2005-06 and 2009- 10 cholesterol:</u><br>Unconditional: -24.01<br>Conditional upon age: -24.54<br>Conditional upon age, other demographics: -24.38<br>Conditional upon age, other demographics, and income: -24.10<br><u>Conditional changes by subgroup:</u>	

						Born 1946-85, some college or more: 2005-06: 313.96, Change 2005-06 to 2009-10: -33.70 (change is statistically significant at P<0.01)<br>Born 1946-85, no college education: 2005-06: 297.84, Change 2005-06 to 2009-10: -11.54<br>Men, born 1946-85, no college: 2005-06: 357.04, Change 2005-06 to 2009-10: -8.11<br>Adults born before 1946: 2005-06: 257.57, change 2005-06 to 2009-10: - 14.01 (change is statistically significant at P<0.1) (estimate is different from that for the group with at least some college education, with p<0.10)<br>**Fibre consumption:**<br><u>Unconditional and conditional differences in mean outcomes between 2005-06 and 2009- 10 in fibre:</u><br>Unconditional: 1.40<br>Conditional upon age: 1.23 (difference from unconditional is statistically significant with p<0.05)<br>Conditional upon age, other demographics: 1.20<br>Conditional upon age, other demographics, and income: 1.16<br>Difference in variable mean between full model (conditional upon age, other demographics and income) and unconditional is statistically significant with p<0.1<br><u>Conditional changes by subgroup:</u>	

						Born 1946-85, some college or more:<br>2005-06: 16.74, Change 2005-06 to 2009-10: 1.60 (change is statistically significant at P<0.05)<br>Born 1946-85, no college education:<br>2005-06: 15, Change 2005-06 to 2009-10: 0.68<br>Men, born 1946-85, no college:<br>2005-06: 17.14, Change 2005-06 to 2009-10: 0.44<br>Adults born before 1946:<br>2005-06: 15.34, Change 2005-06 to 2009-10: 0.71	
Todd, 2017	National: USA<br>Local: n/a	National Health and Nutrition Examination Survey. Includes working age adults between 25-65 (n=12,129), and a secondary sample of 15-24 year olds (23-32 year olds in 2013-2014) (n=5197).	Serial cross-sectional. Used multivariate linear regression models and ordinary least squares. Adjusted for age, household income relative to poverty, household size, and indicators for gender, ethnicity, marital intake, data collected during weekend, and for the older cohort, education as controls.	Exposure: Commence-ment of GR (time)<br>Time point 1: 2005-2006<br>Time point 2: 2007-2008<br>Time point 3: 2009-2010<br>Time point 4: 2013-2014	Percent energy from fat and saturated fat; cholesterol (mg) and fibre (g) consumption. From 1-day dietary recall.	**Percent energy from fat:**<br>Conditional differences with their standard errors, and percentage change in estimated difference from unconditional difference in % energy from fat:<br>2007-08: β −0.16, SE: 0.47<br>2009-10: β −1.05, SE: 0.47, (estimate is statistically significant p<0.05) percentage change: 11<br>2011-12: β −1.11, SE: 0.48 (estimate is statistically significant p<0.05), percentage change: - 14<br>2013-14 β −0.25, SE: 0.45<br>% energy from FAFH: β 0.03, SE: 0.00 (estimate is statistically significant p<0.01)<br>% energy from fast foods: β 0.03, SE: 0.01 (estimate is statistically significant p<0.01)<br>Constant: β 28.71, SE: 0.87 (estimate is statistically significant p<0.01)<br>**Percent energy from saturated fat:**	Selection total: 4<br>Comparability total: 2<br>Outcomes total: 2<br>**Total score: 8**

						Conditional differences with their standard errors, and percentage change in estimated difference from unconditional difference in % energy from saturated fat:<br>2007-08: β −0.18, SE: 0.18<br>2009-10: β −0.66, SE: 0.18 (estimate is statistically significant $p<0.01$), percentage change: 11<br>2011-12: β −0.79, SE: 0.19 (estimate is statistically significant p<0·01), percentage change: 2<br>2013-14 β −0.56, SE: 0.18 (estimate is statistically significant $p<0.01$) percentage change: -4<br>% energy from FAFH: β 0.01, SE: 0.00 (estimate is statistically significant $p<0.01$)<br>% energy from fast foods: β 0.02, SE: 0.00 (estimate is statistically significant $p<0.01$)<br>Constant: β 10.61, SE: 0.43 (estimate is statistically significant $p<0.01$)<br>**Cholesterol consumption (mg)**<br>Conditional differences with their standard errors, and percentage change in estimated difference from unconditional difference in cholesterol:<br>2007-08: β 0.86, SE: 9.96<br>2009-10: β −29.58, SE: 8.21 (estimate is statistically significant $p<0.01$), percentage change: 7<br>2011-12: β −29.44, SE: 8.79 (estimate is statistically significant $p<0.01$), percentage change: -1	

						2013-14 β −19.97, SE: 8.20 (estimate is statistically significant $p<0{\cdot}05$)<br>percentage change: 7<br>% energy from FAFH: β 0.96, SE: 0.13 (estimate is statistically significant $p<0.01$)<br>% energy from fast foods: β −1.09, SE: 0.13 (estimate is statistically significant $p<0.01$)<br>Constant: β 219.01, SE: 18.20 (estimate is statistically significant $p<0.01$)<br>**Fibre consumption (g)**<br>Conditional differences with their standard errors, and percentage change in estimated difference from unconditional difference in fibre:<br>2007-08: β −0.41, SE: 0.72<br>2009-10: β 1.00, SE: 0.56<br>percentage change: 24<br>2011-12: β 2.08, SE: 0.60 (estimate is statistically significant $p<0.01$)<br>percentage change: 6<br>2013-14 β 1.08, SE: 0.50 (estimate is statistically significant $p<0.05$)<br>% energy from FAFH: β −0.02, SE: 0.01 (estimate is statistically significant $p<0.01$)<br>% energy from fast foods: β −0.03, SE: 0.01 (estimate is statistically significant $p<0.01$)<br>Constant: β 14.05, SE: 1.17 (estimate is statistically significant $p<0.01$)	
