# Supplementary material for: Impacts of the 2008 Great Recession on dietary intake: a systematic review and meta-analysis
Source: Int J Behav Nutr Phys Act. 2021 Apr 29;18:57. doi: 10.1186/s12966-021-01125-8 (PMC8084260; doi:10.1186/s12966-021-01125-8)

Additional File 4: Additional Harvest Plots

Figure A1: Harvest plot of studies assessing sources of carbohydrates. Each bar represents a single study.

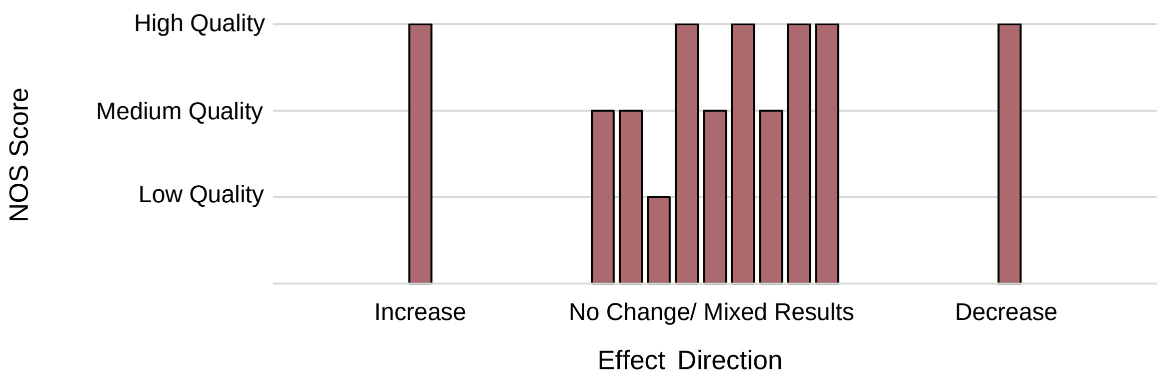

Figure A2: Harvest plot of studies assessing dairy intake. Each bar represents a single study.

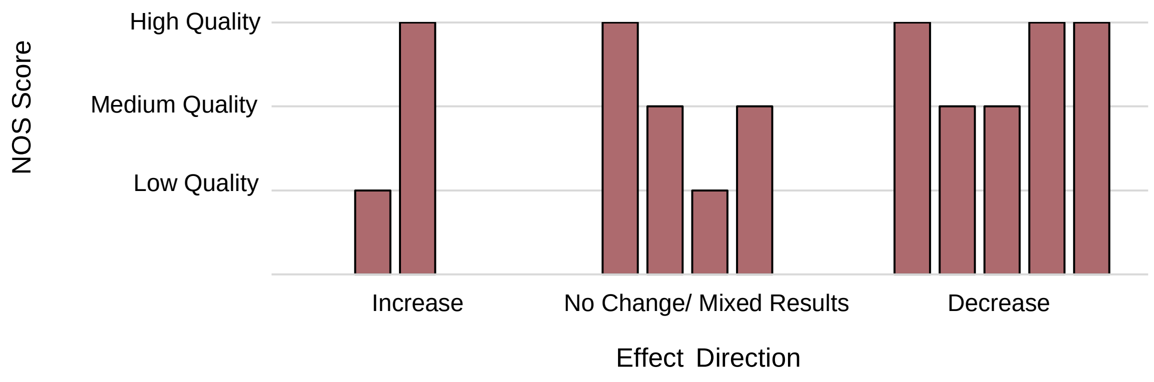

Figure A3: Harvest plot for studies assessing fats and oils intake. Each bar represents a single study.

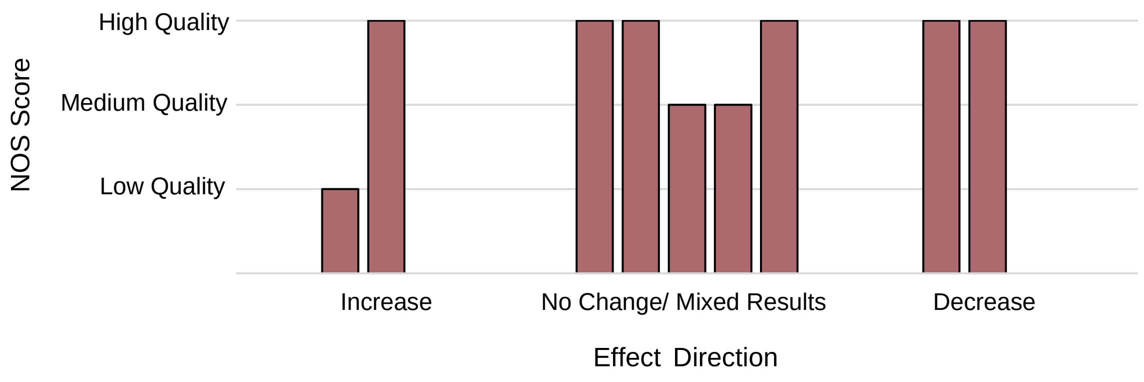

Figure A4: Harvest plot for studies assessing meat intake. Each bar represents a single study.

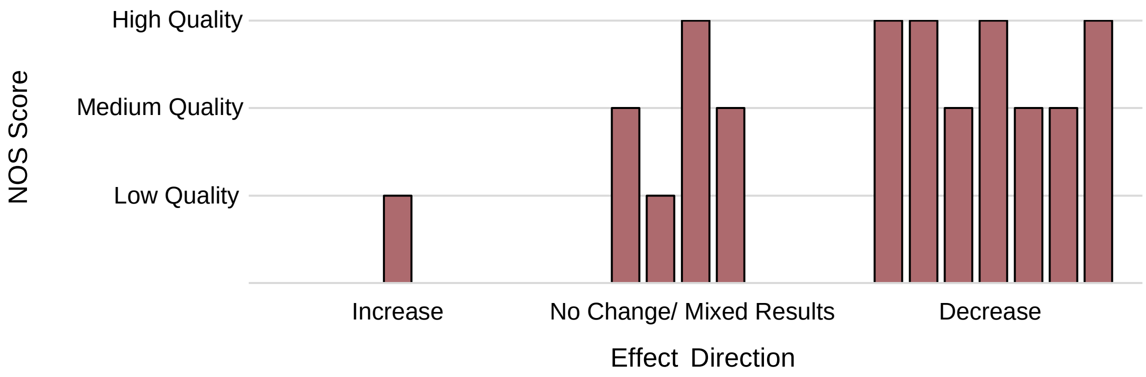

Figure A5: Harvest plot for studies assessing fish intake. Each bar represents a single study.

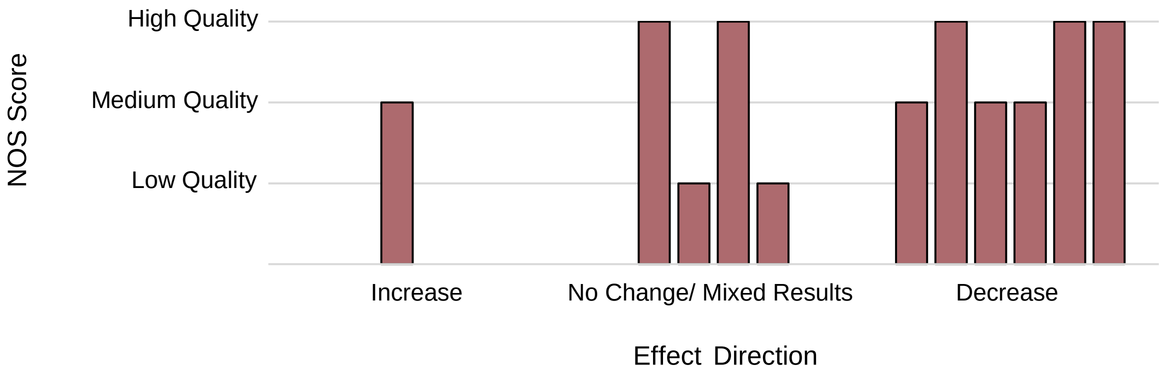

Figure A6: Harvest plot for studies assessing other proteins including legumes and eggs. Each bar represents a single study.

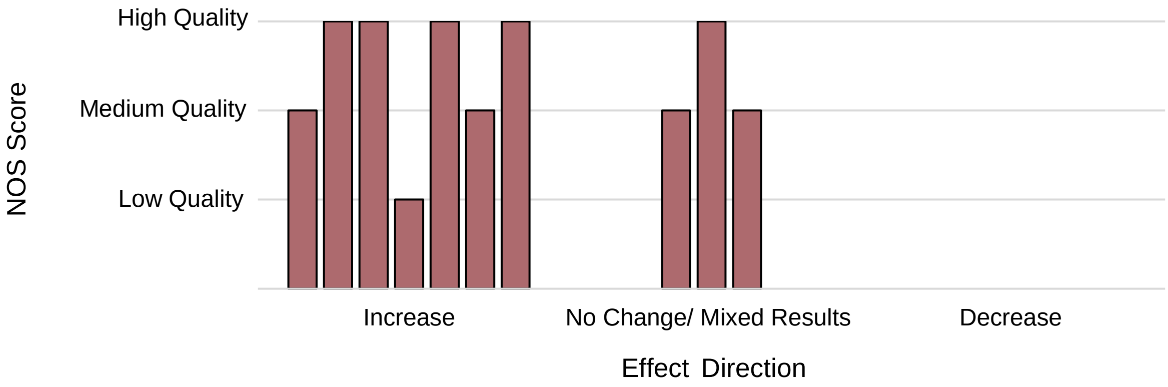

Figure A7: Harvest plot for studies assessing fast food intake. Each bar represents a single study.

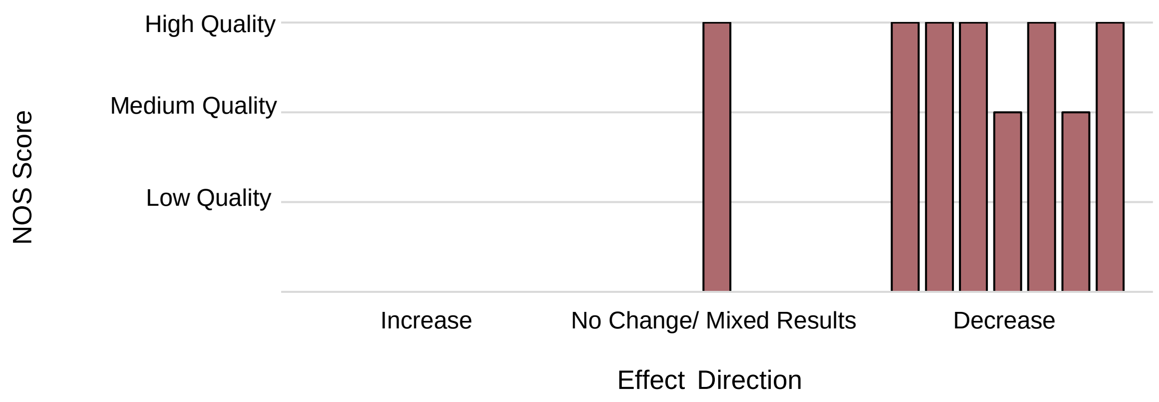

Figure A8: Harvest plot for studies assessing snack intake. Each bar represents a single study.

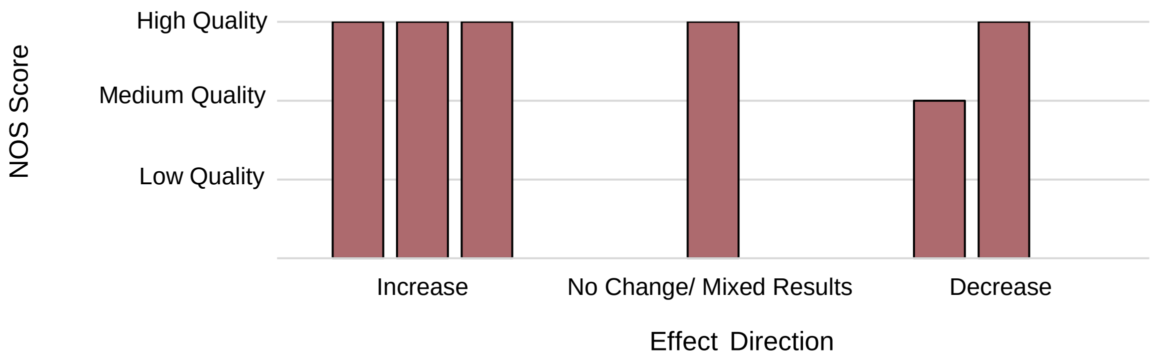

Figure A9: Harvest plot for studies assessing sugar products intake. Each bar represents a single study.

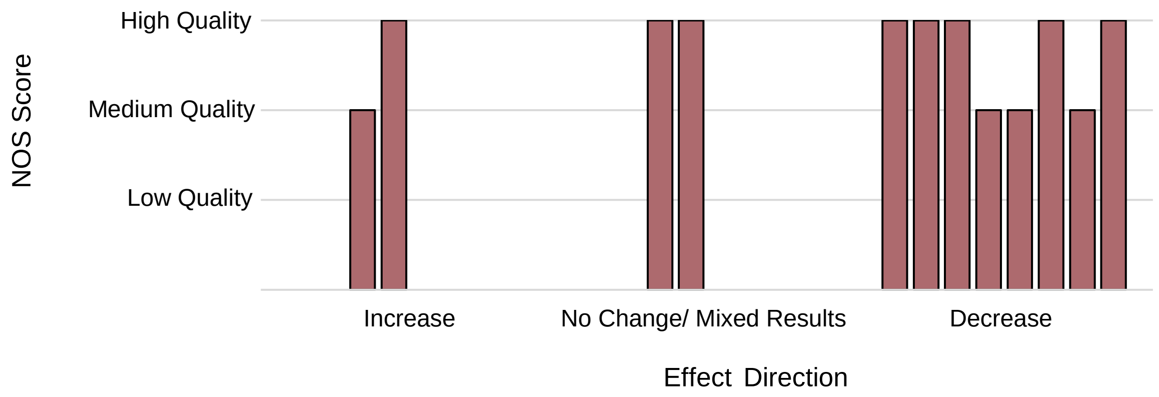

Figure A10: Harvest plot for studies assessing non-alcoholic beverage intake. Each bar represents a single study.

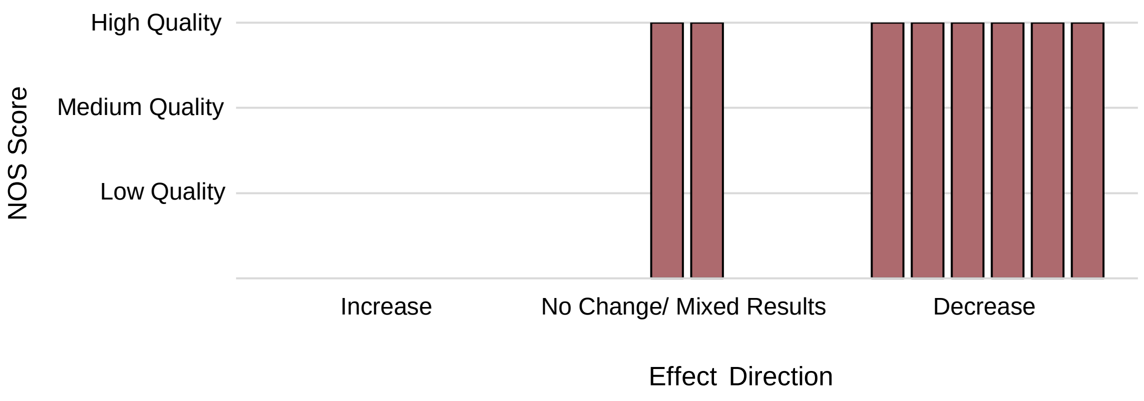

Figure A11: Harvest plot for studies assessing carbohydrate intake. Each bar represents a single study.

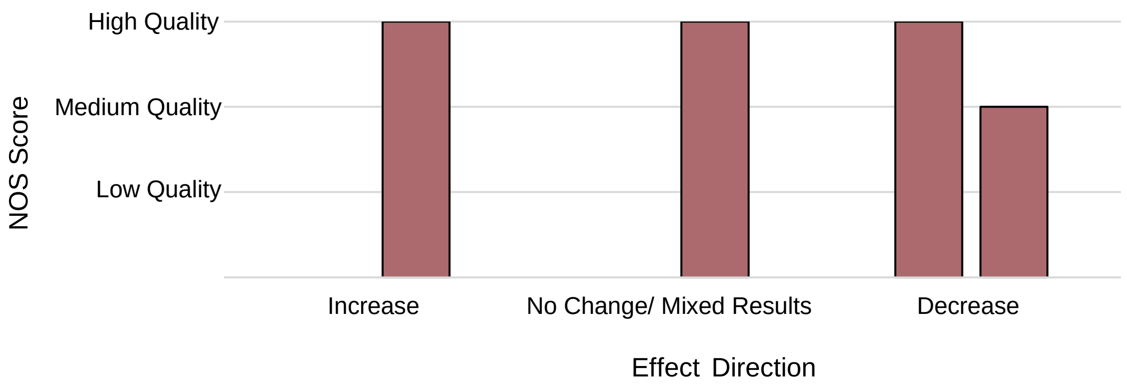

Figure A12: Harvest plot for studies assessing sugar intake. Each bar represents a single study.

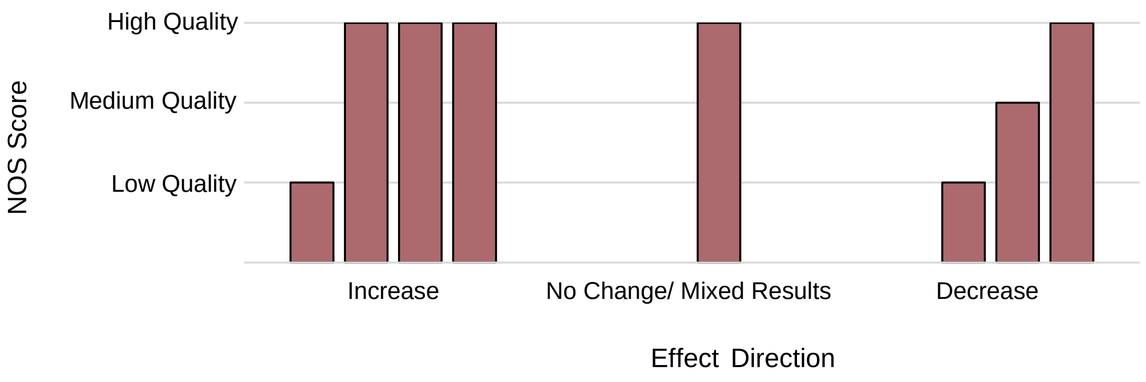

Figure A13: Harvest plot for studies assessing protein intake. Each bar represents a single study.

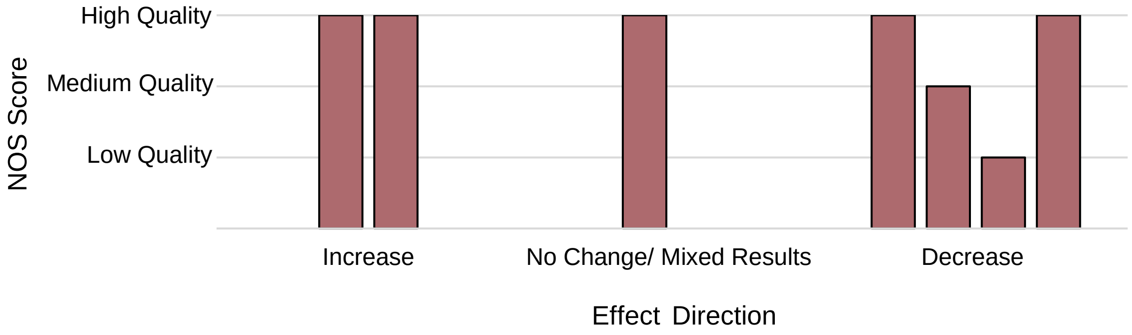

Figure A14: Harvest plot for total fat intake. Each bar represents a single study.

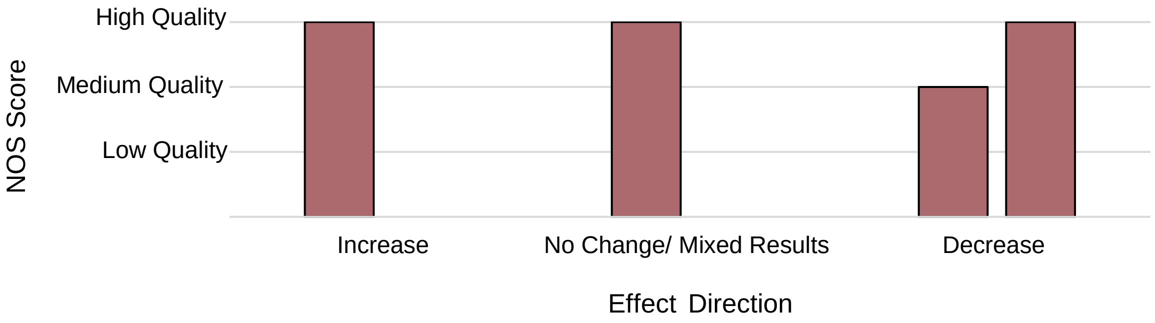

Figure A15: Harvest plot for studies assessing saturated fat intake. Each bar represents a single study.

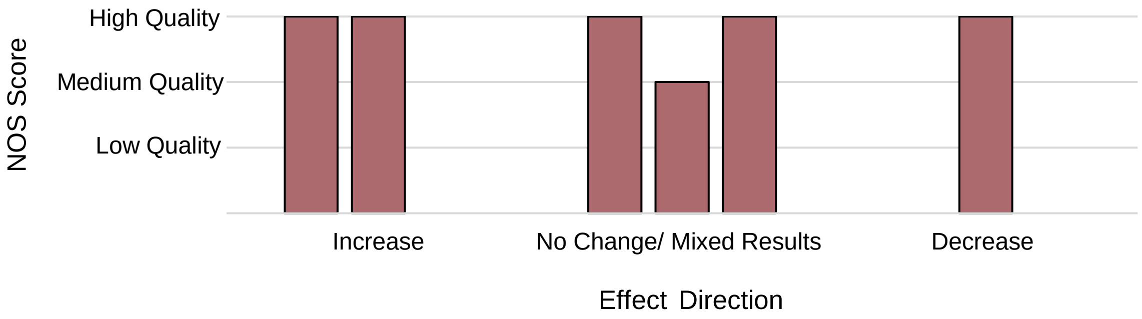

Figure A16: Harvest plot for studies assessing fibre intake. Each bar represents a single study.

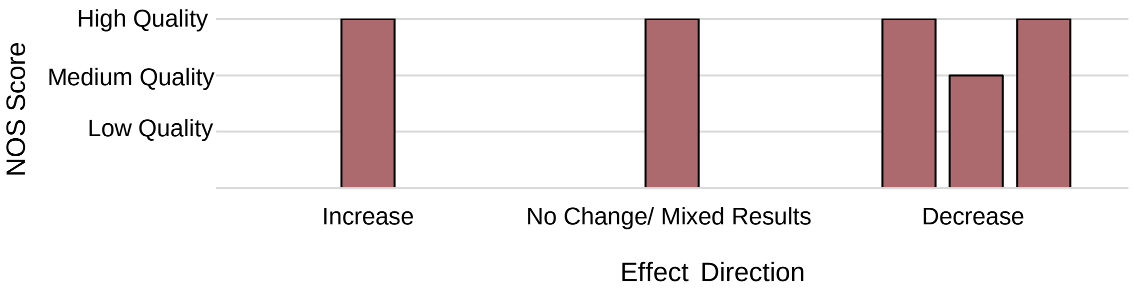

Supplement: Supplementary file 4 — Additional file 4. [file 12966_2021_1125_MOESM4_ESM.pdf]
